# *ent*-Kaurene, *ent*-kauran-16-ol, and shunted diterpenoids from an engineered strain of *Aspergillus oryzae*

**DOI:** 10.1039/d6ra05338f

**Published:** 2026-08-26

**Authors:** Ahmed Arafa, Yuechen Jiang, Jennifer Gerke, Jakob Franke, Russell J. Cox

**Affiliations:** a Institute for Organic Chemistry and BMWZ, Leibniz University Hannover Schneiderberg 38 30167 Hannover Germany russell.cox@oci.uni-hannover.de; b Institute of Botany, Leibniz University Hannover Herrenhäuser Straße 2 30419 Hannover Germany; c Pharmacognosy Department, Faculty of Pharmacy, Tanta University Tanta 31527 Egypt

## Abstract

*Aspergillus oryzae* was engineered to produce high levels of geranylgeranyldiphosphate (GGPP) and *ent*-kaurene, *via ent*-copalyl diphosphate, as precursors for future metabolic engineering projects *via* the expression of *ggs2* and *cps/ks* genes from *Gibberella fujikuroi* under the control of strong constitutive promoters. GGPP was shunted to three new metabolites that were fully identified by NMR and HRMS. *ent*-Copalyl diphosphate was shunted to a further seven new compounds that were also identified by NMR and HRMS. The titre of *ent*-kaurene was significantly increased by heterologous expression of a truncated hydroxymethylglutaryl CoA reductase gene from *Avena strigosa* (*AstHMGR*), while replacement of the dual terpene cyclase encoding gene *cps/ks* with the individual cyclase genes, *ptmT2* from *Streptomyces platensis* and *Bjks* from *Bradyrhizobium japonicum*, resulted mainly in accumulation of *ent*-copalyl diphosphate-derived shunt products. Finally, *ent*-kauran-16-ol was biosynthesised by coexpression of *ggs2* with the bacterial cyclases *ptmT2* and *ptmT3*.

## Abstract 2

*Aspergillus oryzae* was engineered to synthesise *ent*-kaurene and *ent*-kauran-16-ol in titres above 250 mg L^−1^. Ten shunt metabolites were also isolated and identified.

## Graphical abstract

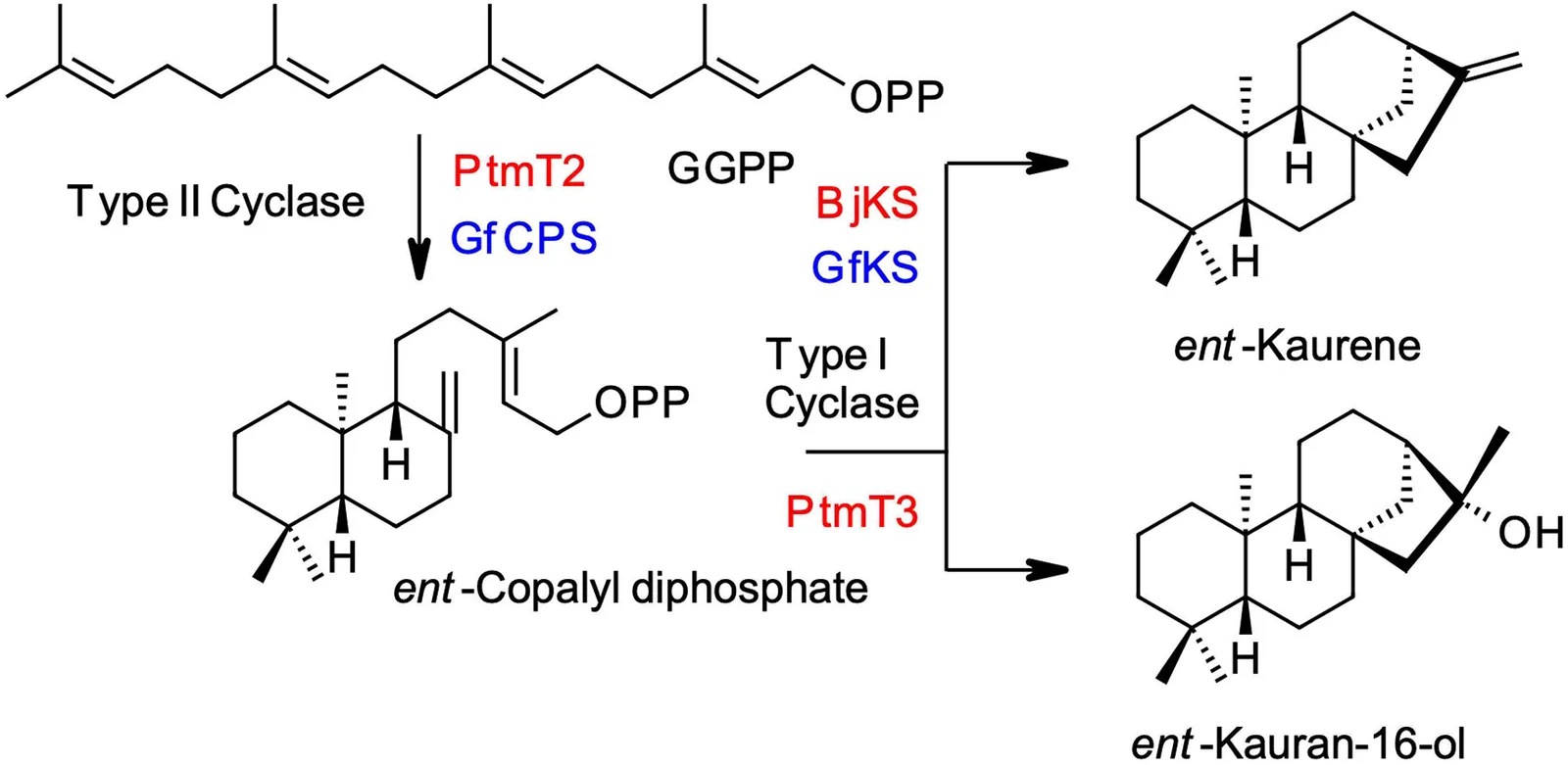

## Introduction

Heterologous expression of biosynthetic genes in suitable host organisms offers an extremely quick, efficient and sustainable method for the synthesis of specialised metabolites (SMs).^1^ It also offers unprecedented opportunities for biosynthetic engineering so that new SMs and small libraries of congeners can be easily created.^2^ We have focussed efforts on the exploitation of a modular expression system that uses the filamentous fungus *Aspergillus oryzae* as the host organism. *A. oryzae* offers the advantages of a clean SM background. This is advantageous in comparison to *Aspergillus nidulans*, for example, that produces many of its own SMs in competition with desired compounds.^3^*A. oryzae* also has the advantage of rapid growth, and the existence of a highly effective toolkit for its genetic manipulation.^4^ Many classes of SMs have been successfully produced in *A. oryzae* by our group and others, including: ergot alkaloids;^5^ indole diterpenoids;^6^ sesquiterpenoids;^7^ diterpenoids;^8,9^ meroterpenoids;^10^ peptides^11^ and hybrid polyketide peptide metabolites;^12^ and both fungal^13^ and bacterial^14^ polyketides. We wished to extend this synthetic flexibility to include SMs derived from *ent*-kaurene 1 that make up one of the largest classes of diterpenoids found in plants, fungi and bacteria.^15^

Compounds derived from *ent*-kaurene 1 and its congeners include the gibberellins such as GA_4_2 that are important plant hormones involved in many aspects of plant growth and development (Fig. 1).^16^ Other key plant metabolites include compounds such as steviol glycosides^17^3 from *Stevia rebaudiana* that are important low-calorie sweeteners widely used in food preparation, and kauralexins^18^ such as 4 that are important components of plant defence. Enmein 5 from *Isodon japonica* is a potent immunosuppressive agent,^19^ while oridonin 6 from *Rabdosia rubescens* is a potent anti-tumour agent.^20,21^ The potent antibacterial compound platensimycin 7 from *Streptomyces platensis* is biosynthesised from the closely related *ent*-kauran-16-ol 8.^22^

**Fig. 1:**
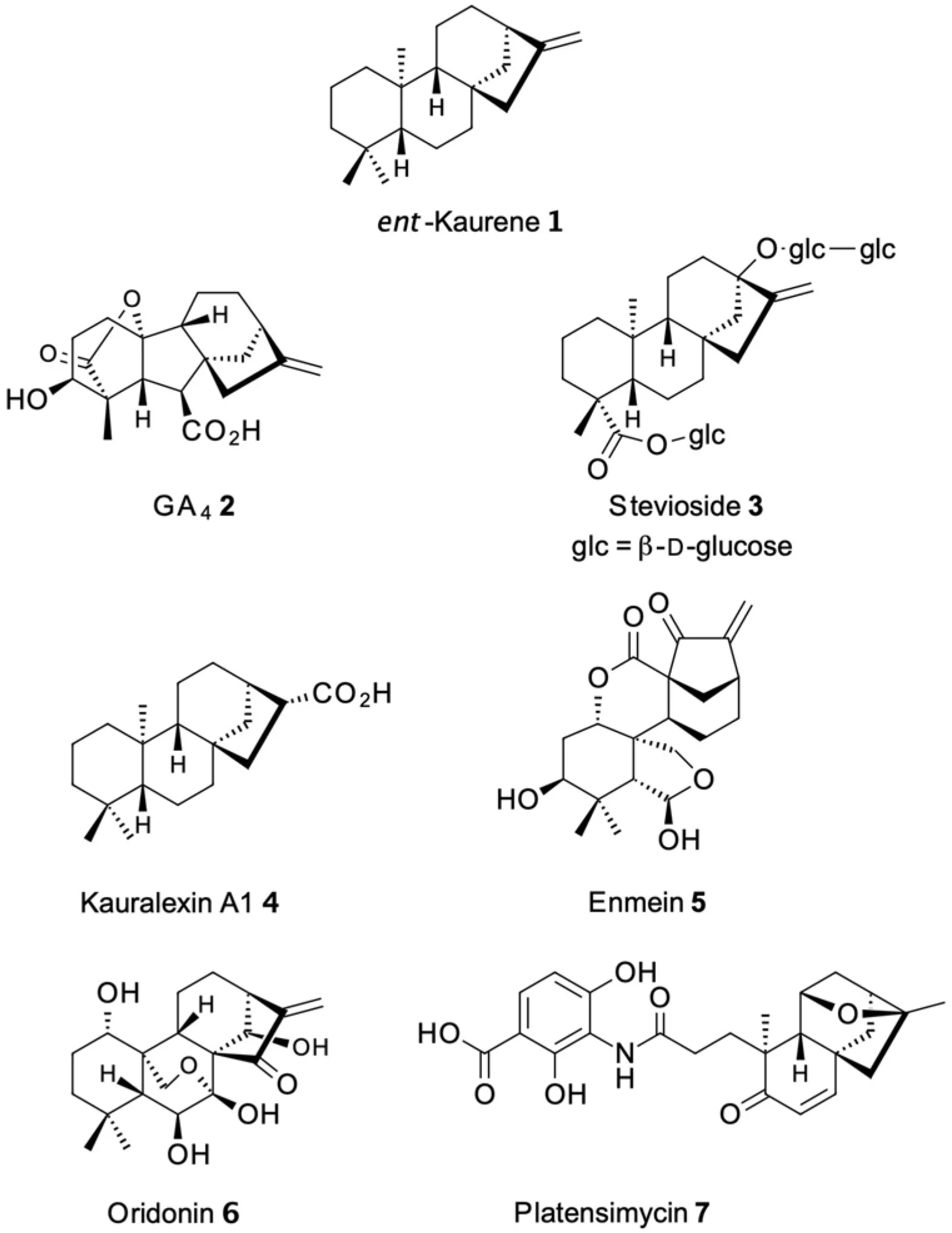
*ent*-Kaurene 1 and key bioactive derivatives.

The creation of a strain of *A. oryzae* that produces *ent*-kaurene 1 or *ent*-kauran-16-ol 8 is the first necessary step prior to synthesis of diverse and more complex diterpenoids. Here we describe the creation and analysis of such strains and analysis of typical shunt metabolites from pathway intermediates.

## Results and discussion

*ent*-Kaurene 1 and *ent*-kauran-16-ol 8 are derived from geranylgeranyl diphosphate 9 (GGPP). This compound is first cyclised to *ent*-copalyl diphosphate 10 by a class II diterpene cyclase known as CPS,^23^ and then cyclised again to *ent*-kaurene 1 or *ent*-kauran-16-ol 8 by a class I diterpene cyclase (Scheme 1).^24^ GGPP 9 is not normally constitutively produced in *A. oryzae*.^25^ We therefore constructed an expression system (pTYGS·*argB*·*ggs2*) featuring the *Gibberella fujikuroi ggs2* gene that encodes GGPP synthase, downstream of the constitutive alcohol dehydrogenase promoter (*P*_adh_) using the modular *A. oryzae* expression system described by Lazarus and co-workers.^4^ GGS2 synthesises GGPP from farnesyl diphosphate (FPP, 11) and dimethylallyl diphosphate (DMAPP, 12). The constructed plasmid also contained a functional copy of *argB*, allowing its selection in the auxotrophic *A. oryzae* strain NSAR1.^26^

**Scheme 1:**
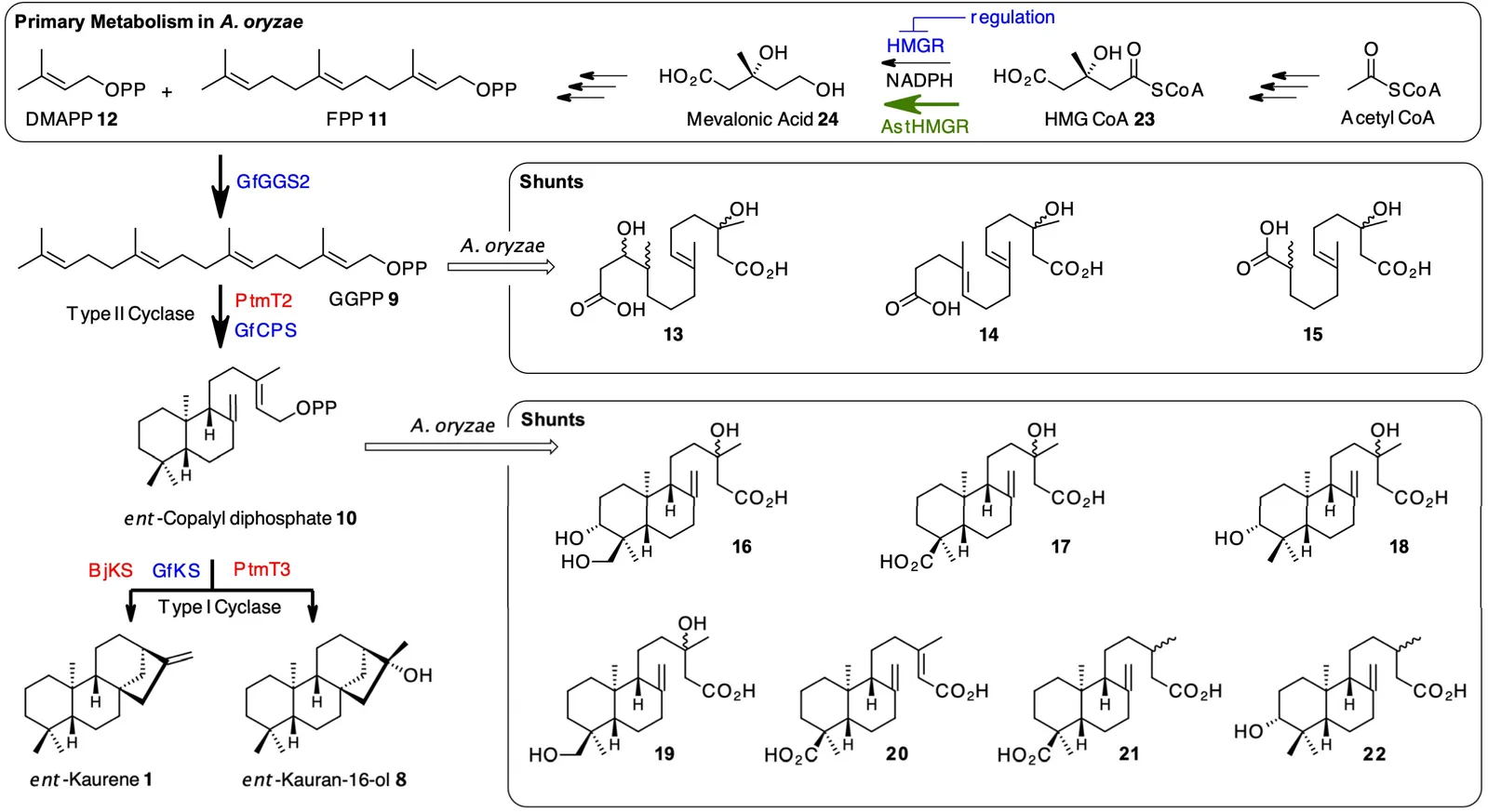
Metabolic pathways and compounds described in this study. Proteins from fungi (GfGGS2, GfCPS, GFKS) are shown in blue; proteins from bacteria (PtmT2, PtmT3, BjKS) are shown in red; and proteins from plants (AstHMGR) are shown in green. *G. fujikuroi* CPS/KS is a bifunctional single enzyme.

Transformation of *A. oryzae* NSAR1 with pTYGS·*argB*·*ggs2* led to the isolation of several transformants that were grown in liquid media for 7 days and then extracted with EtOAc. The concentrated extracts were examined by LCMS and GCMS for the production of new metabolites (Fig. 2C). In comparison with untransformed *A. oryzae* NSAR1, these transformants produced three new compounds 13–15 with measured masses consistent with diterpene-derived structures. Based on their abundance, preparative isolation was performed to enable structural elucidation.

**Fig. 2:**
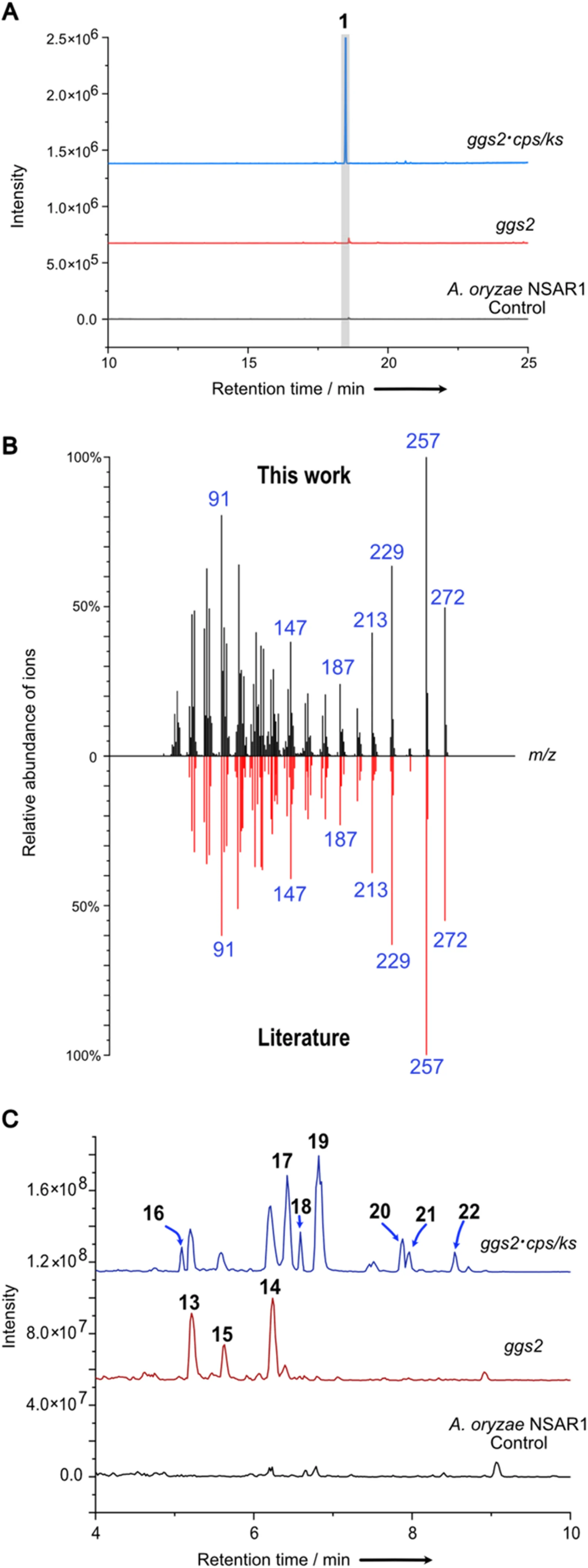
Chromatographic analysis of *A. oryzae* strain expressing *ggs2* and *cps/ks*. (A) GCMS analysis details; (B) GCMS analysis (EIMS) of *ent*-kaurene 1 from panel (A) (black) compared with literature standard (red);^27^ (C) LCMS analysis of *A. oryzae* extracts from the indicated strains. Base peak chromatograms in ES^−^ mode.

High-resolution mass spectrometry (HRMS) and nuclear magnetic resonance (NMR) measurements guided the structural elucidation of the three metabolites (see SI for full NMR and MS characterisation). The stereocentres in compounds 13–15 were not unambiguously determined. The geometry of the alkenes was assumed based on the known geometry of GGPP itself. Based on the loss of the pyrophosphate group, oxidative truncation of the carbon backbone, and the presence of multiple oxygen functionalities, these compounds appear to originate from GGPP 9 through the activity of endogenous *A. oryzae* oxidases or spontaneous oxidation. This appears likely under the conditions set up here where the concentration of GGPP 9 is artificially raised through over-expression of *ggs2.*

Next, the bifunctional *cps/ks* terpene cyclase gene was cloned from *G. fujikuroi* and introduced downstream of the constitutive glyceraldehyde-3-phosphate dehydrogenase promoter (*P*_gpdA_) to create the vector pTYGS·*argB*·*ggs2*·*cps/ks*. Transformation of *A. oryzae* NSAR1 with this vector, and selection as before, led to the isolation of several new transformants. These transformants were grown and organic extracts were examined by GCMS for the detection of non-polar metabolites, and LCMS for the detection of polar metabolites. GCMS analysis clearly indicated the presence of the expected *ent*-kaurene 1 (*ca.* 35 mg L^−1^) by comparison to standard material (Fig. 2A and B). *ent*-Kaurene 1 was detected exclusively in extracts derived from the mycelial fraction, whereas no corresponding peak was observed in the culture supernatant, indicating that this highly non-polar diterpene is not secreted by *A. oryzae*.

The LCMS analysis showed the presence of at least seven new polar compounds 16–22 in the transformants that were not present in the untransformed NSAR1 strain (Fig. 2C). The measured masses of these components suggested they could be GGPP 9 derivatives.

These seven compounds were purified by mass-directed HPLC and their structures fully elucidated by NMR and HRMS (see SI for details). All seven compounds are clearly derived from *ent*-copalyl diphosphate 10, since they are not observed to be produced in the absence of the cyclase. We therefore assumed the absolute stereochemistry shown, and have no evidence to suggest that enantiomeric products may have been formed.

To evaluate alternative terpene cyclases for *ent*-kaurene 1 formation, bacterial enzymes were examined. Bacteria require two distinct enzymes to generate the fully cyclized diterpene backbone in contrast to the bifunctional fungal CPS/KS. The platensimycin 7 pathway of *Streptomyces platensis* is initiated by the enzyme PtmT2, which converts GGPP 9 to *ent*-copalyl diphosphate 10. The *ptmT2* gene was optimised for *A. oryzae* expression by exchange of rare codons and reduction of overall GC content to 58% and cloned downstream of *P*_gpdA_ in the pTYGS-*argB-ggs2* vector. This vector was introduced into *A. oryzae* in order to assess the enzyme ability to generate pathway intermediates in the heterologous host, as reflected by the formation of the shunt metabolites described above. Metabolite analysis revealed strongly increased accumulation of shunt products 16–22, approximately 5–10-fold (by comparison of metabolites LCMS peak area, Fig. 3, see SI for details) across independent transformants, indicating high catalytic activity of PtmT2.

**Fig. 3:**
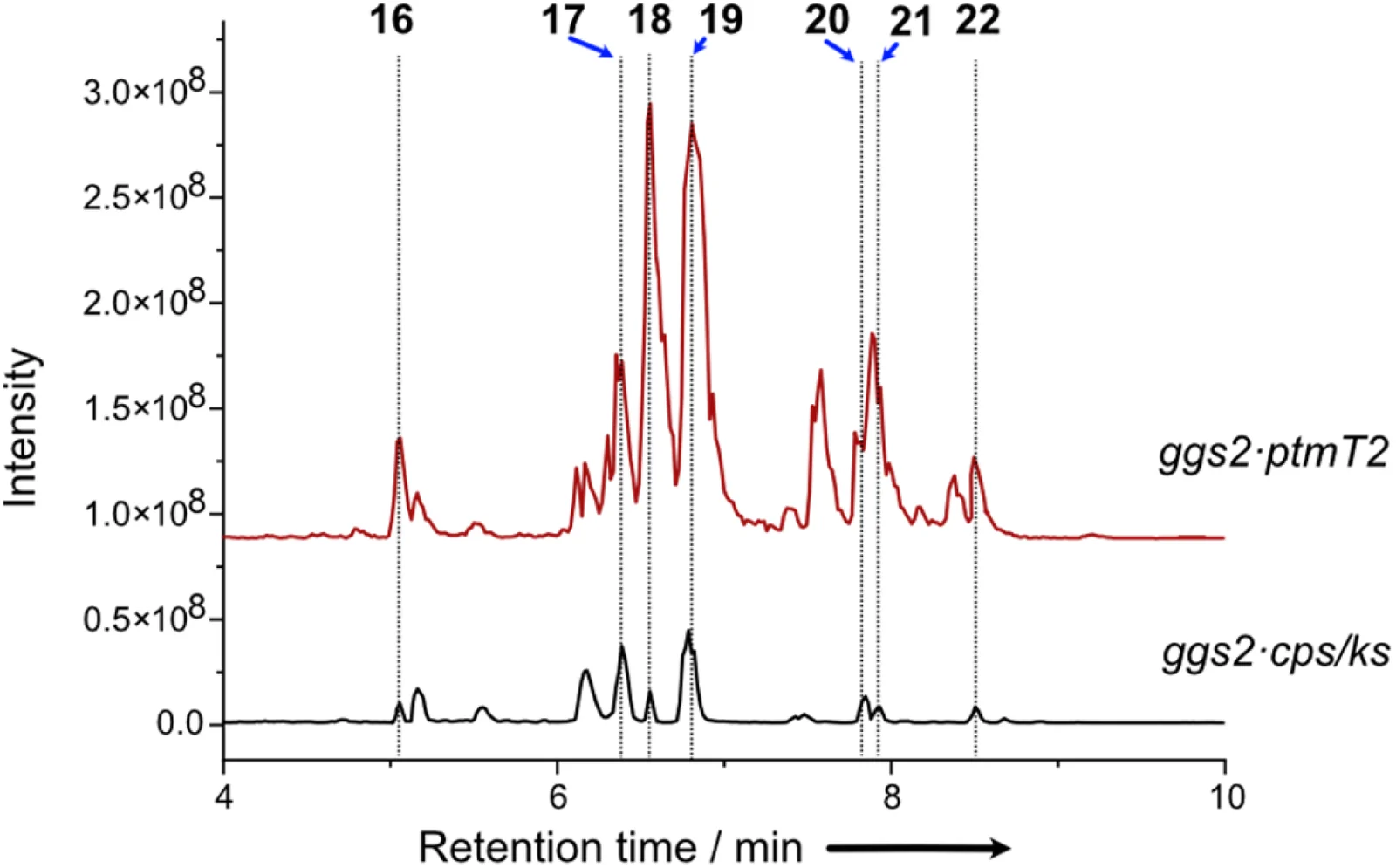
Comparison of production of shunts 16–22 from *ent*-copalyl diphosphate 10 produced by PtmT2 (red) or CPS/KS (black). Base peak LCMS chromatograms in ES^−^ mode.

Because *S. platensis* naturally produces *ent*-kauran-16-ol 8 rather than *ent*-kaurene 1, a dedicated kaurene synthase was required. The *Bjks* gene (optimised to 60% GC) from the bacterium *Bradyrhizobium japonicum* was therefore cloned downstream of *P*_gpdA_ in the pTYGS·*adeA* vector and coexpressed together with pTYGS·*argB*·*ggs2*·*ptmT2*. This resulted in the expected formation of *ent*-kaurene 1, confirming that BjKS is functional in *A. oryzae*. However, relatively high levels of *ent*-copalyl-derived shunt metabolites 16–22 were still observed, indicating that while BjKS is active, PtmT2 remains the dominant enzyme in the PtmT2/BjKS synthetic module.

Next, we introduced a system to increase throughput in the pathway to GGPP 9. The rate-limiting step in terpene biosynthesis in eukaryotes is usually performed by hydroxymethylglutaryl (HMG) CoA reductase (HMGR, Scheme 1) that converts HMG CoA 23 to mevalonic acid 24. This step is normally highly regulated, but a truncated HMGR (tHMGR) from the plant *Avena strigosa* (black oat) is known to lack feedback inhibition and is highly active.^28^ We therefore introduced *AstHMGR* downstream of the strong enolase promoter (*P*_eno_) to create the vector pTYGS·*argB*·*ggs2*·*cps/ks*·*AstHMGR*.

This was used to transform *A. oryzae* NSAR1, and several transformants were analysed for metabolite production *vs.* untransformed *A. oryzae*. Analysis of the transformants by GCMS indicated the production of *ent*-kaurene 1 as before. Quantitative GCMS measurements indicated an at-least 7-fold increase in titre of this metabolite after expression of *AstHMGR* (Fig. 4). Purification from mycelia indicated a titre of *ent*-kaurene 1 of 253 ± 38 mg L^−1^.

**Fig. 4:**
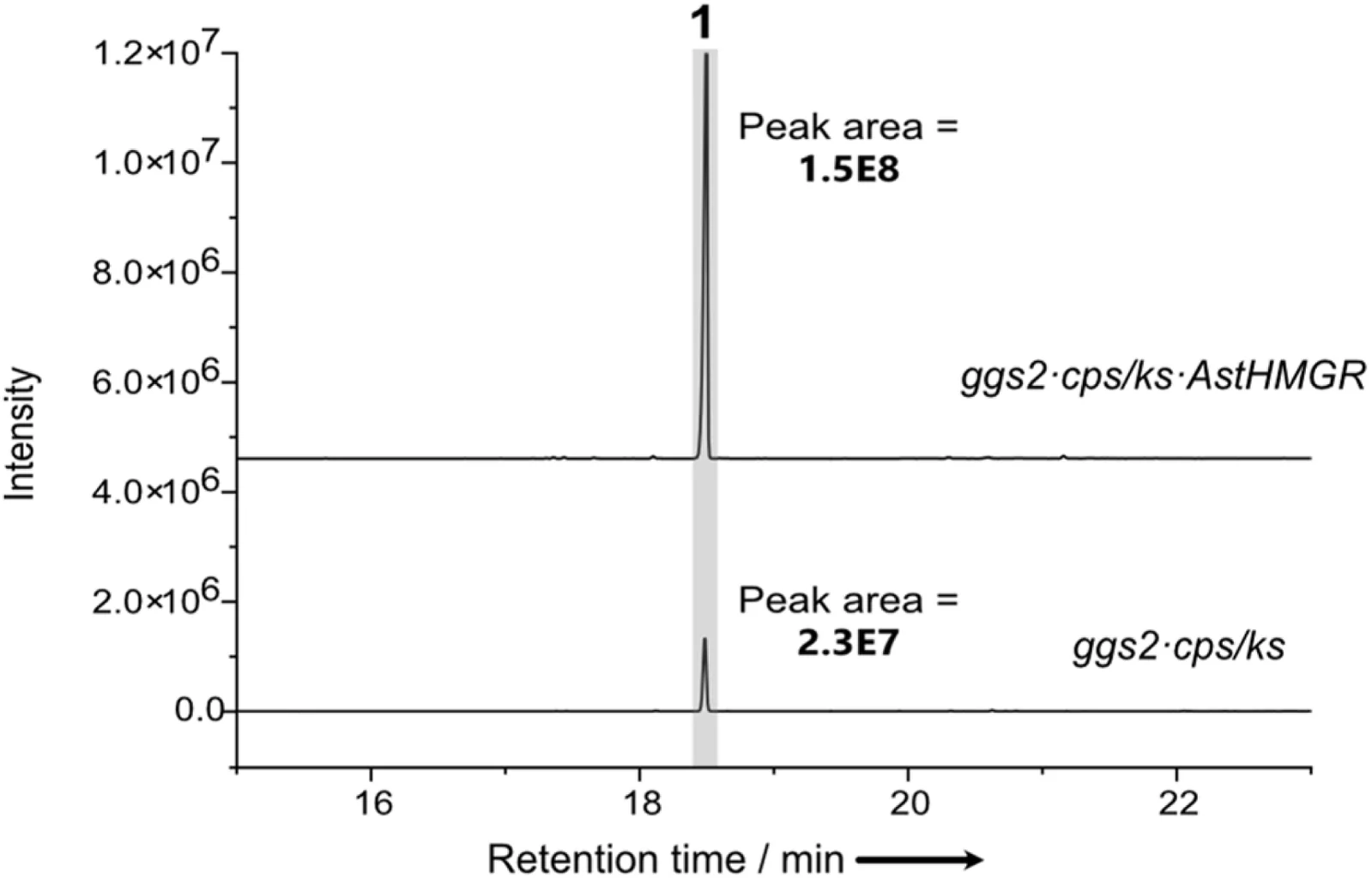
Increased titre of *ent*-kaurene 1 upon coexpression of *AstHMGR*.

Finally, we set up the biosynthesis of *ent*-kauran-16-ol 8. This compound is synthesised from *ent*-copalyl diphosphate 10 in *S. platensis* by the type I terpene cyclase PtmT3. The gene *ptmT3* (optimised to 63% GC and rare codons exchanged) was introduced downstream of *P*_gpdA_ in pTYGS·*adeA*, to create pTYGS·*adeA*·*ptmT3* and coexpression of this with pTYGS·*argB*·*ggs2*·*ptmT2* in *A. oryzae* NSAR1 resulted in successful formation of *ent*-kauran-16-ol 8 (GCMS, Fig. 5A). GCMS data of the detected compound matched very well with the published fragmentation pattern of *ent*-kauran-16-ol 8 supporting the compound's identity (Fig. 5B).

**Fig. 5:**
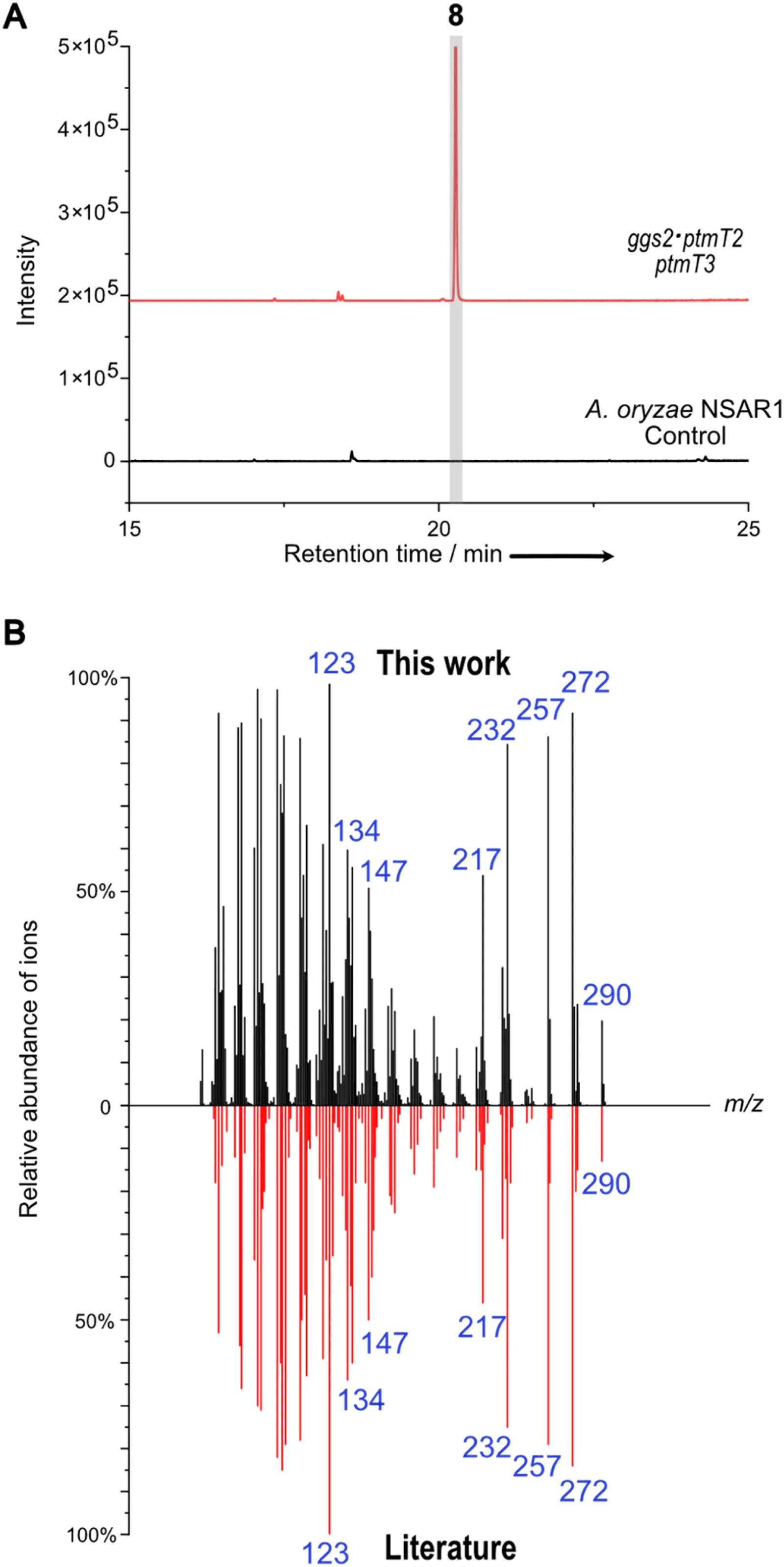
Analysis of *A. oryzae* NSAR1 *ggs2*·*ptmT2*·*ptmT3* producing *ent*-kauran-16-ol 8. (A) GCMS analysis; (B) GCMS analysis (EIMS) of *ent*-kauran-16-ol 8 from panel (A) (black) compared with literature standard (red).^29^

## Conclusion

Expression of *G. fujikuroi ggs2* and *cps/ks* was successful in *A. oryzae*, producing the expected *ent*-kaurene 1 and ten fully characterised shunt metabolites. The titre of *ent*-kaurene 1 was dramatically increased to *ca.* 250 mg L^−1^ by overcoming HMGR repression by the coexpression of *AstHMGR*. Alternatively, expression of *ggs2*, *ptmT2* and *ptmT3* led to the synthesis of *ent*-kauran-16-ol 8. It is clear that the host organism *A. oryzae* can shunt accumulated pathway intermediates such as GGPP 9, and *ent*-copalyl diphosphate 10 to unwanted metabolites such as 13–15 and 16–22.

These compounds appear to be formed as a result of unnatural accumulation to high concentrations in attenuated pathways. This is supported by our observation in another study that these shunt compounds become much less prominent when downstream pathway steps are included, for example towards gibberellins.^16^

Never-the-less, the observation of the production of these shunts gives a useful indication that early pathway steps are functional, and their characterisation will aid future engineering campaigns towards more complex diterpenoids.

It is noteworthy that the filamentous fungus *A. oryzae* acted as an effective host for the combination of biosynthetic genes from three different kingdoms: truncated *AstHMGR* from a plant; *ggs2* and *cps/ks* from a fungus; and codon-optimised *ptmT2*, *ptmT3* and *Bjks* from bacteria. No other engineering work was required to provide the desired *ent*-kaurene 1 or *ent*-kauran-16-ol 8 in good titres. Future work will focus on the development of this strain as a platform for the total biosynthesis of *ent*-kaurene- and *ent*-kauran-16-ol-derived SMs.

## Author contributions

All experimental work was performed by AA and YJ. Compound characterisation was performed by AA and YJ, and AA, YJ and RJC solved the structures. RJC, JG and JF devised and supervised the project. The MS was prepared by RJC and AA, and polished by all authors.

## Conflicts of interest

RJC is joint Editor-in-Chief of *RSC Advances*.

## Supplementary Material

RA-016-D6RA05338F-s001

## Data Availability

The data supporting this article have been included as part of the supplementary information (SI) including NMR spectra, LCMS data and further experimental details. Supplementary information: all experimental and characterisation details. See DOI: https://doi.org/10.1039/d6ra05338f.
