# Supplementary material for: ent-Kaurene, ent-kauran-16-ol, and shunted diterpenoids from an engineered strain of Aspergillus oryzae
Source: RSC Adv. 2026 Aug 26;16(48):63932–7. doi: 10.1039/d6ra05338f (PMC13508058; doi:10.1039/d6ra05338f)
Supplement: Supplementary file 1 [file RA-016-D6RA05338F-s001.pdf]

Supplementary Information (SI) for RSC Advances.
This journal is © The Royal Society of Chemistry 2026

Ent-Kaurene, *Ent*-Kauran-16-ol, and Shunted Diterpenoid Metabolites from an Engineered Strain of *Aspergillus oryzae*

Ahmed Arafa, Yuechen Jiang, Jennifer Gerke, Jakob Franke and Russell J. Cox

Electronic Supplementary Information

**1.**	**Construction of Vectors**	**2**
**1.1**	**Gene sources**	**2**
**1.2**	**Cloning Procedures and Vector Construction**	**4**
**2.**	**Transformation and Selection of *A. oryzae***	**4**
**3.**	**LCMS and GCMS Analysis**	**5**
**3.1**	**Extraction Procedure**	**5**
3.1.1	Extraction from fungal cultures	5
3.1.2	Extraction of nonpolar diterpenes from fungal biomass	5
**3.2**	**Analytical LCMS**	**5**
**3.3**	**GCMS Analysis**	**5**
**3.4**	**Quantification of 16 – 22 production by CPS/KS *vs* PtmT2**	**6**
**4.**	**Purification and Chemical Characterisation of Compounds**	**7**
**4.1**	**Preparative LCMS Instrument and Programme**	**7**
**4.2**	**NMR Instrument Details**	**7**
**4.3**	**HRMS**	**7**
**4.4**	**NMR Characterisation of Isolated Compounds**	**8**
4.4.1	6-*E*-3,12-Dihydroxy-3,7,11-trimethyltetradec-6-enedioic acid **13**	8
4.4.2	4-*E*,8-*E*-12-Hydroxy-4,8,12-trimethyltetradeca-4,8-dienedioic acid **14**	12
4.4.3	6-*E*-10-Hydroxy-2,6,10-trimethyldodec-6-enedioic acid **15**	15
4.4.4	3α,13,18-Trihydroxy-*ent*-labd-8(17)-en-15-oic acid **16**	18
4.4.5	13-Hydroxy-*ent*-labd-8(17)-en-15,18-dioic acid **17**	22
4.4.6	3α,13-Dihydroxy-*ent*-labd-8(17)-en-15-oic acid **18**	26
4.4.7	*ent*-13,18-Dihydroxy-*ent*-labd-8(17)-en-15-oic acid **19**	30
4.4.8	*ent*-Labd-8(17),13-dien-15,18-dioic acid **20**	34
4.4.9	*ent*-Labd-8(17)-en-15,18-dioic acid **21**	38
4.4.10	3α-Hydroxy-*ent*-labd-8(17)-en-15-oic acid **22**	42
**4.5**	**HRMS data of Isolated Compounds**	**46**

1. Construction of Vectors

1.1 Gene sources

All genes used in this study, except *AstHMGR*, were obtained as synthetic genes from Twist Bioscience (Berlin, Germany) or GeneWiz (Azenta Life Sciences, Leipzig, Germany). The synthesized sequences contained overhangs required for yeast homologous recombination and are listed in Table S1. Primers used for *AstHMGR* amplification and cloning, as well as sequencing primers, are listed in Table S2.

Table S1. Gene sequences used in this study. Overhangs for cloning are shown in bold and underlined.

**Gene name and information**	**Nucleotide Sequence (**Overhang sequence underlined)
ggs2<br>• From *Gibberella fujikuroi*<br>• Overhang for yeast homologous recombination cloning downstream of P_{adh} promotor	**TCTTTCAACACAAGATCCCAAAGTCAAAGG**ATGGCTGAACAACAGATCTCCAACCTTCTTTCAATGTTTGATGCTTCTCACGCAAGCCAGAAGTTGGAG<br>ATTACGGTTCAGATGATGGATACCTACCATTACAGAGAAACTCCTCCAGACTCTTCCTCTTCAGAAGGCGGTTCCTTATCTCGCTATGATGAGCGACGG<br>GTCTCCCTTCCGCTCTCTCACAATGCAGCCTCCCCAGACATAGTCTCCCAGTTATGCTTCTCAACAGCTATGAGCTCGGAGCTCAATCACAGGTGGAAG<br>TCACAGCGCCTCAAGGTTGCTGACTCTCCCTACAACTACATCCTGACTCTTCCATCTAAAGGTATTCGTGGGGCTTTCATTGACTCACTGAATGTCTGG<br>CTCGAGGTCCCCGAAGACGAGACCTCGGTGATCAAAGAGGTGATTGGCATGCTCCACAACTCGTCTCTCATAATCGATGACTTCCAAGACAACTCCCCA<br>CTTCGGCGGGGCAAGCCATCTACACATACTGTCTTCGGTCCAGCACAAGCAATCAACACAGCAACATATGTCATCGTCAAGGCCATCGAGAAAATACAG<br>GATATCGTCGGTCACGATGCATTGGCAGATGTAACTGGCACTATAACCACAATCTTCCAGGGTCAGGCAATGGATCTGTGGTGGACTGCTAATGCCATT<br>GTTCCGTCTATCCAAGAATATCTCCTGATGGTCAATGACAAGACTGGTGCCCTGTTCAGGTTATCGCTTGAACTACTGGCGCTGAACTCTGAAGCATCC<br>ATCAGTGACAGCGCGCTTGAATCTCTCAGCAGCGCTGTCTCACTGCTCGGGCAGTATTTCCAGATAAGAGATGATTACATGAATCTCATTGACAACAAG<br>TATACTGATCAGAAAGGATTTTGCGAGGATCTGGACGAGGGGAAATACTCGTTGACTCTAATCCATGCTCTGCAGACCGACTCCAGCGACCTTCTCACC<br>AACATCTTATCGATGAGAAGAGTCCAAGGAAAACTTACGGCGCAGCAAAAGATGCTGGTTTTGGAAGTGATGAAAACAAACGGCAGCTTAGACTGGACG<br>AGCAAACTCCTTGGCATGCTGCACACAAGAGTTGTAGCCGAGATTGAGAGCCTGGAAGTTAGCACGAAGAGGGATAACCATGCATTGAGGGCTTTAGTG<br>GAACGCCTGAAGCTGGAAACCTAG**CGCGCCAGGGAACATGTTCATAACGCATAG**
cps/ks<br>• From *Gibberella fujikuroi*<br>• Overhang for yeast homologous recombination cloning downstream of P_{gpdA} promotor	**CAGCTACCCCGCTTGAGCAGACATCACCGG**ATGCCTGGCAAAATCGAGAACGGCACCCCCAAAGACCTCAAGACAGGAAATGACTTTGTATCAGCAGCA<br>AAGTCGCTGCTGGACCGCGCCTTCAAGAGCCATCATAGCTACTATGGTCTATGTTCCACCAGCTGTCAGGTATATGACACTGCTTGGGTGGCCATGATA<br>CCCAAGACGAGAGACAATGTCAAGCAATGGCTGTTTCCTGAGTGCTTCCACTATCTTCTGAAGACTCAGGCCGCTGATGGTAGTTGGGGTAGTCTCCCC<br>ACGACCCAGACTGCTGGTATTCTAGACACTGCTTCAGCTGTGCTTGCACTCTTATGTCACGCACAAGAGCCTCTGCAGATACTGGACGTCTCGCCAGAC<br>GAGATGGGTCTCAGGATCGAGCACGGGGTAACTTCTTTGAAGCGACAGCTAGCTGTTTGGAACGATGTTGAAGATACCAATCACATTGGTGTCGAGTTT<br>ATTATTCCAGCTTTACTTTCCATGCTTGAGAAAGAGCTCGACGTGCCATCTTTTGAGTTTCCTTGCAGAAGTATCTTGGAGAGGATGCACGGAGAGAAG<br>CTCGGCCATTTCGATTTGGAGCAAGTATATGGCAAGCCGTCTTCACTACTTCACTCCCTTGAAGCCTTTCTTGGAAAGCTTGACTTTGATCGCCTTTCT<br>CACCATCTTTATCATGGCTCTATGATGGCTTCACCTTCTTCCACTGCAGCCTACCTCATTGGTGCAACCAAGTGGGACGACGAGGCTGAAGATTACCTG<br>AGGCACGTTATGAGAAATGGTGCTGGTCACGGGAATGGTGGCATCTCAGGCACCTTTCCTACAACACATTTTGAGTGTAGCTGGATCATAGCGACACTC<br>CTGAAGGGTGGCTTCACTTTGAAACAAATCGATGGTGATGGCTTGCGCGGCTTGTCAACTATACTTCTCGAAGCGTTGAGGGACGAGAATGGAGTAATT<br>GGCTTTGCACCCCGTACTGCTGATGTTGATGACACTGCAAAAGCTCTTCTGGCATTGAGCCTTGTCAATCAACCTGTCAGCCCAGATATCATGATCAAG<br>GTCTTTGAAGGAAAAGATCACTTCACCACGTTCGGATCTGAACGTGATCCCAGCTTGACTTCCAACCTCCATGTGCTGCTGAGTCTCCTGAAGCAGTCC<br>AATCTATCGCAATACCACCCACAAATCCTGAAGACCACGTTGTTCACTTGCCGATGGTGGTGGGGCAGCGACCATTGCGTCAAAGATAAATGGAATTTG<br>AGTCATCTATACCCAACCATGCTTCTGGTGGAAGCTTTCACCGAGGTACTTCATCTCATTGATGGTGGCGAGCTTTCTAGTCTCTTTGACGAGAGCTTC<br>AAGTGCAAGATCGGCCTGTCAATCTTCCAGGCTGTACTACGTATCATCCTCACCCAAGATAACGACGGATCTTGGAGAGGATATCGCGAGCAAACATGC<br>TACGCCATTCTAGCTCTTGTACAGGCACGCCATGTATGTTTCTTCACTCACATGGTGGACAGGCTACAGAGTTGCGTCGACCGTGGCTTTTCGTGGCTC<br>AAGTCCTGCAGCTTTCATTCTCAGGACCTCACTTGGACGTCAAAGACAGCATATGAGGTTGGGTTTGTTGCTGAAGCGTACAAGCTAGCAGCCCTTCAG<br>TCAGCAAGCCTAGAGGTGCCCGCCGCTACTATTGGCCACAGCGTCACCTCGGCGGTTCCATCATCGGACTTGGAGAAGTACATGAGGCTTGTGCGCAAG<br>ACTGCTTTGTTCTCGCCACTGGACGAGTGGGGACTCATGGCGTCAATCATTGAGTCCTCGTTCTTCGTGCCCCTTCTACAAGCTCAGCGCGTTGAGATT<br>TATCCGAGGGACAACATCAAGGTCGACGAGGACAAATACCTGAGCATCATTCCTTTTACTTGGGTCGGATGCAACAACAGATCCCGCACATTTGCATCT<br>AACAGATGGCTCTACGATATGATGTACCTGTCCCTCCTGGGCTACCAGACCGATGAGTACATGGAAGCTGTCGCTGGGCCGGTATTCGGTGATGTCTCT<br>CTGCTTCACCAGACCATCGACAAGGTCATAGACAACACAATGGGAAACTTAGCCAGGGCCAATGGTACCGTGCACAGTGGCAATGGTCATCAGCATGAG<br>TCACCCAACATTGGACAAGTTGAGGATACTCTCACTCGCTTCACCAACAGCGTGCTCAACCACAAAGATGTTCTGAATTCAAGCTCTTCTGACCAAGAC<br>ACTCTTCGCCGAGAATTCAGAACGTTCATGCATGCCCATATCACACAGATAGAGGACAACAGTCGCTTCTCCAAGCAGGCCTCCAGCGACGCTTTCTCT<br>TCCCCAGAACAATCGTACTTCCAATGGGTCAACAGTACAGGAGGCAGTCACGTCGCCTGCGCGTACTCATTCGCTTTCTCCAACTGCCTAATGTCAGCG<br>AATCTGCTCCAAGGCAAGGACGCATTTCCCTCCGGCACTCAGAAATATCTCATTTCATCCGTTATGCGTCACGCTACAAATATGTGTCGTATGTACAAC<br>GACTTTGGGTCCATAGCTCGCGATAACGCTGAGCGGAATGTGAACTCGATCCACTTCCCCGAGTTCACGTTATGTAACGGAACGTCACAGAATCTCGAT<br>GAAAGGAAAGAGAGACTTTTAAAGATAGCTACGTATGAGCAAGGTTATCTTGATCGTGCTCTCGAAGCTCTGGAACGACAGTCTCGTGATGATGCAGGT<br>GATCGTGCTGGGTCTAAGGATATGAGGAAGCTCAAGATTGTAAAACTGTTCTGTGATGTCACCGACTTGTATGATCAGCTTTACGTGATCAAGGACCTT<br>TCAAGCAGCATGAAGTGA**CGCGCCGGTCATGATTGATGATATGGACAT**
ptmT2<br>• From *Streptomyces platensis*<br>• Optimized for *Aspergillus* expression<br>• Overhang for yeast homologous recombination cloning downstream of P_{gpdA} promotor	**CAGCTACCCCGCTTGAGCAGACATCACCGG**ATGCTGGAAGTACCAGCACAACCTACTCCAGCTCCACGTGAAGCTGAAGCTGCTGCACTGCTGGCTGCT<br>ACCGTTGCAGACCCGTGGGGTCTGGTTGCTCCAAGCGTATACGATACCGCTCGTCTGGTAAGCCTGGCACCGTGGCTGGACGGTCATCGTGAACGCCTG<br>GGTTACCTGGTTAAAGAACAAAACCAGGACGGTTCTTGGGGTGCTCCAGATGGCTATGGTCTGGTACCGACCCTGTCTGCAGTTGAAGCACTGCTGACC<br>GAACTGGCACGTACTGATTCTGGCGCACCGCACCCGCCACATGATGATCTGGCTGCAGCTTGTGCAGGTGGTCTGGGTGCCCTGCAGGATGGTCTGCTG<br>GCAGGTCCAGTTCCGGATACGATTGGTGTGGAATTTGTTGCTCCTTCTCTGCTGGCGGATATCAACACCCGTCTGGCTGCCCTGACGGAACAGGCTCCA<br>GGTAAACTGGGTGCTTGGTCTGGTACCACCCTGACGTCTCCGGCACCTGACCTGGATGGTGCTCTGCTGGCAGGTGTTCGCGAAATGACCGAACAGGCA<br>CCGCTGCCGGAAAAACTGTGGCATACCCTGGAAGCAATTACGCGTGACGGTACTCGTGGTGCACGTCCACACGAAGGTGCTCCGCCACACAATGGTTCT<br>GTAGGTTGTAGCCCGGCAGCAACTGCTGCATGGCTGGGTGCTTCTCCAGATCCAGCTGCACCAGGCGTTGCATACCTGCGCGATGTACAGGCACGTTTC<br>GGTGGTCCGGTACCGTCCATCACCCCGATCGTTTACTTCGAACAGGCTTGGGTTCTGAATTCTCTGGCCGCTTCTGGTCTGCGTTATGAAGCTCCGGCA<br>GCGCTGCTGGATAGCCTGGAAGCTGGTCTGACCGATGAAGGTATTGCAGCAGCACCGGGCCTGCCGTCTGATTCTGACGATACCGCTGCGGTACTGTTC<br>GCTCTGGCTCAGCACGGTCGTACCCACCGTCCAGACTCCCTGATGCACTTTCGTCGTGACGGTTATTTCTCTTGCTTTGGCGTAGAACGTACCCCGTCT<br>ACCTCTACCAACGCTCACATCCTGGAGGCTCTGGGCCACCACGTCACTGTACGTCCAGATGACGCAGGCCGCTATGGCGCTGAAATCCGTATGATCTCT<br>GATTGGCTGCTGGATAACCAGCTGCCAGACGGCTCTTGGATGGACAAATGGCATGCATCCCCGTATTACGCGACCGCTTGTTGCGCACTGGCTCTGGCA<br>GAATTCGGTGGTCCATCTGCCCGTGCAGCAGTTGATCGTGCTGCAGCATGGGCGCTGGAAACTCAGCGTGCAGATGGTTCTTGGGGTCGTTGGCAGGGT<br>ACGACCGAAGAAACTGCGTACATGGTTCAGCTGCTGATGCGTACTCGTACTCCGGGTTCTCCTGGTACTGTGGCACGTTCTGCAGCTCGTGGTTGCGAT<br>GCACTGCTGGCCCACGATGATCCGGCGTCTTATCCAGGTCTGTGGCATGATAAAGATATCTACGCACCAGTAACCGTTATCCGTGCAGCTCGTCTGGCT<br>GCTCTGGCACTGGGTGGTGCTGAATCCGCTGCTTCTGGCGGTGCTTGA**CGCGCCGGTCATGATTGATGATATGGACAT**
ptmT3<br>• From *Streptomyces platensis*<br>• Optimized for *Aspergillus* expression<br>• Overhang for yeast homologous recombination downstream of P_{gpdA} promotor	**CAGCTACCCCGCTTGAGCAGACATCACCGG**ATGAACAACAAACTGGTGAGCCATTTACTGGATAGCGCGGACCCGGCGGTAAGGGCGAAGGCGATCGAG<br>GCTGCTGCATTAGCGCGCCAATGGGCCCGCCCGGTGCAGCAAGAACTGAGCGATAAAGTGTATGATACCCTGGCGGCGACCGTGTGCGTGATTGCGCCG<br>GAACTGGATGTGGCGGATCATGCGCTGCTGGTGGAATATAGCCTGTGGCTGTACCTGTTAGATGGACGGCTGGATGATTTTGAACACTACGGCACCCGC<br>CCTGAGGATGTGGGCCGCCGCGTCATTGCGGTACTGCGCGGAGGCCGCGCGGAAGCGCGCGCGGATGATTTTTTTGAAACGAGCCTGGCGGCGCTGGTC<br>GAAGAGCTGCGCACCCGCGATGGCTGCTGCGGCCTGCTGGAGCGTTTTGTGCTTCGACTCGTTGATGGCGTGCGCGCGGGGGTGCGTCAAGCGGTGCTG<br>AGCCGTCGCATTGCGGAAGGCGCGGAACCGCTGCCGACCATGGAAGATTTTCTGGAACTGGCGTATCGCCATGTGAACTATCGCAGCGTGGCGCTGGCG<br>CTGCTGATAACAGTGGGGGAGCGCCCGGACAGTGCGGCGCAAGAGCGACTGGATGCTGCCCTCGTCCCGGCAAGCCGTGCAGTGCGCCTGGCGAACGAT<br>CTGCGCACCTGGGCGAAAGATGCGGAAGAAGGCACCCTGAACGTGGTGCAGCTGGTGGCGGGCGATGGCACCCCGATGACCCCGCATGCGGTGCGCGAT<br>CGCATTCAAGCGTATCGCGATGAACAAGCGCATCTGCTGGATGAGCTTCATCGCAGCGCGCCGGCGAGCGCCGCGGCGCTGGAAAACAACCTGCGCGTG<br>GCGATTGATCTGTATACCGTGGATGATCTGCAGTTTGATCTGCCGGATGCGGAACAAGCGGTGGGCTAA**CGCGCCGGTCATGATTGATGATATGGACAT**

Bjks<br>• From *Bradyrhizobium japonicum*<br>• Optimized for *Aspergillus* expression<br>• Overhang for yeast homologous recombination cloning downstream of P_{gpdA} promotor	**CAGCTACCCCGCTTGAGCAGACATCACCGG**ATGATCCAGACCGAACGTGCAGTTCAACAAGTACTGGAATGGGGCCGCTCTCTGACCGGTTTCGCGGAC<br>GAACACGCAGTTGAAGCCGTACGTGGCGGCCAGTACATCCTGCAGCGTATTCACCCGTCTCTGCGCGGTACCAGCGCGCGTACCGGTCGCGATCCGCAG<br>GATGAAACCCTGATTGTGACCTTCTACCGCGAGCTGGCTCTGCTGTTCTGGCTGGACGATTGTAACGACCTGGGTCTGATCTCCCCGGAACAGCTGGCC<br>GCTGTTGAACAGGCGCTGGGCCAGGGTGTGCCGTGTGCACTGCCAGGTTTTGAAGGCTGCGCAGTTCTGCGTGCGAGCCTGGCAACTCTGGCATACGAC<br>CGTCGTGACTACGCTCAGCTGCTGGACGATACCCGCTGTTACTCCGCAGCACTGCGCGCCGGTCATGCGCAGGCTGTTGCTGCAGAGCGTTGGTCTTAC<br>GCCGAATACCTGCACAACGGCATTGACTCCATTGCCTACGCGAACGTTTTCTGTTGTCTGAGCCTGCTGTGGGGCCTGGACATGGCGACTCTGCGTGCT<br>CGTCCGGCATTTCGTCAAGTACTGCGCCTGATCTCCGCCATTGGCCGTCTGCAAAACGATCTGCATGGCTGCGACAAAGATCGTAGCGCGGGCGAAGCT<br>GATAACGCCGTAATTCTGCTGCTGCAGCGTTACCCGGCTATGCCGGTTGTTGAGTTCCTGAACGATGAACTGGCTGGCCACACTCGCATGCTGCACCGT<br>GTAATGGCGGAAGAACGTTTCCCAGCACCGTGGGGTCCGCTGATCGAAGCTATGGCAGCTATCCGCGTTCAGTACTACCGTACGTCCACCTCCCGCTAC<br>CGTAGCGACGCAGTACGCGGCGGTCAGCGTGCACCGGCTTGA**CGCGCCGGTCATGATTGATGATATGGACAT**
AstHMGR<br>• From *Avena strigosa*	ATGGCGCCCGAGAAAATGCCCGAGGAGGACGAGGAAATCGTCGCCGGGGTCGTCGCAGGGAAGATCCCCTCCTACGTGCTCGAGACCAGGCTAGGCGAC<br>TGCCGCAGGGCAGCCGGGATCCGCCGCGAGGCGCTGCGCCGGATCACCGGCAGGGAGATCGACGGCCTTCCCCTCGACGGCTTCGACTACGACTCGATT<br>CTCGGACAGTGCTGCGAGATGCCCGTCGGGTACGTGCAGCTGCCGGTCGGCGTCGCGGGGCCGCTCGTCCTCGACGGCCGCCGCATATACGTCCCGATG<br>GCCACCACGGAGGGCTGCCTAATCGCCAGCACCAACCGCGGATGCAAGGCCATTGCCGAGTCCGGAGGCGCATCCAGCGTCGTGTACCGCGACGGGATG<br>ACCCGCGCCCCCGTAGCCCGCTTCCCCTCCGCACGACGCGCCGCAGAGCTCAAGGGCTTCCTGGAGAATCCGGCCAACTACGACACCCTGTCCGTGGTC<br>TTTAACAGATCAAGCAGATTTGCAAGGCTGCAGGGGGTCAAGTGCGCCATGGCTGGGAGGAACTTGTACATGAGGTTCACCTGCAGCACCGGGGATGCC<br>ATGGGGATGAACATGGTCTCCAAGGGCGTCCAAAATGTGCTCGACTATCTGCAGGAGGACTTCCCTGACATGGACGTTGTCAGCATCTCAGGCAACTTT<br>TGTTCCGACAAGAAATCAGCTGCTGTAAACTGGATTGAAGGCCGTGGAAAGTCCGTGGTTTGTGAGGCAGTAATCAGAGAGGAAGTTGTCCACAAGGTT<br>CTCAAGACCAACGTTCAGTCACTCGTGGAGTTGAATGTGATCAAGAACCTTGCTGGCTCAGCAGTTGCTGGTGCTCTTGGGGGTTTCAACGCCCACGCA<br>AGCAACATCGTAACGGCTATCTTCATTGCCACTGGTCAGGATCCTGCACAGAATGTGGAGAGCTCACAGTGTATCACTATGTTGGAAGCTGTAAATGAT<br>GGCAGAGACCTTCACATCTCCGTTACAATGCCATCTATCGAGGTGGGCACAGTTGGTGGAGGCACGCAGCTGGCCTCACAGTCGGCCTGCTTGGACCTA<br>CTGGGCGTCAAAGGCGCCAACAGGGAATCTCCGGGGTCGAACGCTAGGCTGCTGGCCACGGTGGTGGCTGGTGCCGTCCTAGCTGGGGAGCTGTCCCTC<br>ATCTCCGCCCAAGCTGCCGGCCATCTGGTCCAGAGCCACATGAAATACAACAGATCCAGCAAGGACATGTCCAAGATCGCCTGCTGA

Table S2. Primers used in this study. Overhangs for cloning are shown in bold and underlined.

Name	Sequence	Purpose
AstHMGR-Peno-F	**CGACTGACCAATTCCGCAGCTCGTCAAAGG**ATGG CGCCCGAGAAAATGCC	For yeast homologous recombination cloning of *AstHMGR* downstream of P_{eno} promotor
AstHMGR-Peno-R	**CTGGTAGACGTCATATAATCATACGGCGCG**TCA GCAGGCGATCTTGGACA	
OAR15-F	CCAACCATGCTTCTGGTGGA	For sequencing of *cps/ks* from the middle (forward)
OAR16-R	CGTCCAAGTGAGGTCCTGA	For sequencing of *cps/ks* from the middle (reverse)
Padh-Plug-F	ATTCACCACTATTATTCCCACCCTATAATA	For sequencing and colony PCR of genes downstream of P_{adh} promotor
Padh-Plug-R	GAGACGAAACAGACTTTTTCATCGCTAAAA	
PgpdA-Plug-F	CTTTTCTTTTCTCTTTCTTTTCCCATCTTC	For sequencing and colony PCR of genes downstream of P_{gpdA} promotor
PgpdA-Plug-R	TGACCTCCTAAAACCCCAGTG	
Peno-Plug-F	CTTCTTAAATATCGTTGTAACTGTTCCTGA	For sequencing and colony PCR of genes downstream of P_{eno} promotor
Peno-Plug-R	CTAGTAGCTATAGTCTCCCAATATACTTCG	

1.2 Cloning Procedures and Vector Construction

The expression vectors pTYGS-*argB* and pTYGS-*adeA* were used for heterologous expression in *Aspergillus oryzae*. DNA constructs were assembled by yeast homologous recombination using *Saccharomyces cerevisiae* CEN.PK2 cells (Euroscarf) using a LiOAc/PEG-based transformation protocol. Yeast cells were cultivated on YPAD agar plates at 30 °C for 3 days. A single colony was used to inoculate 10 mL YPAD medium and grown overnight at 30 °C with shaking at 200 rpm. The overnight culture was transferred to 40 mL fresh YPAD medium in a 250 mL Erlenmeyer flask and incubated for an additional 4–5 h under the same conditions.

Cells were harvested by centrifugation at 3000 × *g* for 5 min and washed twice with 25 mL sterile distilled water. The resulting pellet was resuspended in sterile water and aliquoted. For transformation, frozen aliquots of competent yeast cells are thawed for 20 min on ice, then cells are harvested by centrifugation at 14000 × *g* for 2 min. The resulting yeast pellet was mixed with 240 µL PEG 3350 (50% w/v), 50 µL denatured salmon sperm DNA (2 mg/mL), 36 µL 1 M lithium acetate, and 34 µL DNA mixture containing linearized vector backbone and DNA inserts with the appropriate homologous overhangs in equimolar amounts. The transformation mixture was incubated at 42 °C for 50 min.

Following transformation, cells were collected by centrifugation, resuspended in 1 mL sterile water, and 200 µL were spread onto selective SM-Ura agar plates. Plates were incubated at 30 °C for 3–5 days. Plasmids were isolated from yeast using the Zymoprep™ Yeast Plasmid Miniprep II kit (Zymo Research) and propagated in *E. coli* for verification.

2. Transformation and Selection of *A. oryzae*

A. oryzae NSAR1 (Lazarus group, Bristol) was used as the heterologous host, and fungal transformation was performed using PEG-mediated protoplast transformation. *A. oryzae* NSAR1 was cultivated on DPY agar plates at 28 °C for 7–12 days. Mycelium from these plates was used to inoculate 50 mL GN medium in 250 mL shake flasks, which were incubated at 28 °C and 110 rpm for 18–20 h. Biomass was separated from the culture medium by filtration through Miracloth (Millipore) and incubated with 10 mL of VinoTaste® Pro solution (10 mg/mL; sterilized through a 0.45 µm filter, Roth) at room temperature with gentle shaking (5 rpm) for 3–4 h. Protoplasts were released by gentle pipetting and collected by filtration through Miracloth followed by centrifugation at 3000 × *g* for 5 min. The resulting pellet was resuspended in 1 mL of transformation solution containing 0.8 M NaCl, 10 mM $CaCl_2$, and 50 mM Tris-HCl (pH 7.5). DNA constructs (1–2 µg each construct) were mixed with 100 µL of the protoplast suspension and incubated on ice for 2 min. Subsequently, 1 mL of PEG-based transformation solution (60% [w/v] PEG 3350, 0.8 M NaCl, 10 mM $CaCl_2$, 50 mM Tris-HCl, pH 7.5) was added, followed by incubation at room temperature for 20 min.

The transformation mixture was combined with 5 mL of selective soft agar and overlaid onto selective agar plates, which were incubated at 28 °C for 4–5 days. For transformations with pTYGS-*argB*, arginine was omitted from the selective medium, whereas both arginine and adenine were omitted for co-transformations with pTYGS-*argB* and pTYGS-*adeA*. Emerging colonies were subjected to two rounds of re-selection on fresh selective agar plates (2 days each) to confirm positive transformants. Verified transformants were maintained on DPY agar plates for 7 days. Spores were subsequently used to inoculate 100 mL DPY liquid medium in 500 mL shake flasks for cultivation and metabolite production. Cultures were incubated at 28 °C and 110 rpm for 7 days.

3. LCMS and GCMS Analysis

3.1 Extraction Procedure

3.1.1 Extraction from fungal cultures

For extraction of metabolites from fungal culture broth, the fungal biomass was removed from the culture medium by vacuum filtration and the filtrate was collected. The filtrate was acidified to pH 3–4 using hydrochloric acid and extracted twice with ethyl acetate. The combined organic phases were dried over anhydrous magnesium sulfate, filtered, and concentrated to dryness under reduced pressure using a rotary evaporator. The resulting residue was dissolved in MeOH for subsequent analysis.

3.1.2 Extraction of nonpolar diterpenes from fungal biomass

For selective extraction of nonpolar diterpenes, including *ent*-kaurene and *ent*-kauran-16-ol, fungal biomass was separated from the culture medium by filtration, and the aqueous phase was discarded. The collected mycelium was blended with MeOH (200 mL) to disrupt the cells and extract intracellular metabolites. The suspension was stirred at room temperature for 1 h and subsequently filtered to remove residual solids. The filtrate was concentrated to dryness under reduced pressure using a rotary evaporator.

The residue was resuspended in a mixture of water (100 mL) and cyclohexane (150 mL) and vigorously shaken to partition nonpolar compounds into the organic phase. The cyclohexane layer was collected, dried over anhydrous magnesium sulfate, and concentrated to dryness under reduced pressure. The final residue was dissolved in cyclohexane, filtered through a cotton-plugged Pasteur pipette, and directly subjected to GCMS analysis.

3.2 Analytical LCMS

Analytical liquid chromatography mass spectrometry (LCMS) data was obtained using a Waters LCMS system, which consists of a Waters 2767 autosampler, Waters 2545 pump, a Phenomenex Kinetex column (2.6 µm, C_{18}, 100 Å, 4.6 × 100 mm) equipped with a Phenomenex Security Guard precolumn (Luna, C_5, 300 Å). The system was used at a flow rate of 1 mL/min. Detection was carried out by a diode array detector (Waters 2998) in the range 210 to 600 nm and an ELSD detector (Waters 2424) together with a mass spectrometer, Waters SQD-2 mass detector, operating simultaneously in ES^+ and ES^- modes between 150 and 1000 *m/z*. Typically, a solvent gradient was run over 15 min starting at 10 % acetonitrile/ 90 % HPLC grade water (0.05 % formic acid) and ramping to 90 % acetonitrile. Crude extracts were dissolved in MeOH at approximately 10 mg/mL, filtered through a cotton-plugged Pasteur pipette, and transferred to LCMS vials for analysis.

3.3 GCMS Analysis

GCMS measurements were performed using an HP6890N gas chromatograph coupled to a 5973N mass selective detector. Compound separation was achieved on an OPTIMA 5 MS capillary column (30 m × 0.25 mm internal diameter, 0.25 µm film thickness; Macherey-Nagel, Düren, Germany). Helium was used as the carrier gas at a constant flow rate of 1.5 mL/min. Samples (1 µL) were injected using a split ratio of 1:20. The oven temperature program started at 50 °C and was increased to 320 °C at a rate of 10 °C/min, resulting in a total run time of 32 min. Data acquisition and analysis were carried out using Agilent MSD ChemStation software (version F.01.03.2357). Published reference data were digitized using Mass Spec Calculator Professional 5.1.

3.4 Quantification of 16 – 22 production by CPS/KS *vs* PtmT2

Each extract from 100 mL culture (grown in a 250 mL flask) was dissolved in MeOH (5.00 ml) before LCMS analysis. The autosampler was set to 25 µL to ensure that the injection loop was completely filled, corresponding to a 20 µL injection in all cases. The extracted ion chromatogram corresponding to $[M - H]^-$ was integrated using MassLynx software.

compound number	LCMS ES^- EIC peak area / arbitrary units							
	cps/ks transformant 1	*cps/ks* transformant 2	*cps/ks* transformant 3	*average / E+09*	*ptmT2* transformant 1	*ptmT2* transformant 2	*ptmT2* transformant 3	*average / E+09*
**16**	5.20 E+09	6.10 E+09	4.10 E+09	**5.13**	45.0 E+09	47.0 E+09	53.0 E+09	**48.3**
**17**	31.0 E+09	29.0 E+09	21.0 E+09	**27.0**	152 E+09	162 E+09	149 E+09	**154**
**18**	7.00 E+09	25.0 E+09	29.0 E+09	**20.3**	231 E+09	268 E+09	205 E+09	**235**
**19**	35.0 E+09	37.0 E+09	34.0 E+09	**35.3**	238 E+09	272 E+09	210 E+09	**240**
**20**	6.10 E+09	6.30 E+09	5.80 E+09	**6.07**	52.1 E+09	61.3 E+09	48.1 E+09	**53.8**
**21**	3.90 E+09	4.20 E+09	3.70 E+09	**3.93**	32.1 E+09	38.4 E+09	31.2 E+09	**34.2**
**22**	3.60 E+09	3.70 E+09	3.10 E+09	**3.47**	25.2 E+09	26.1 E+09	21.6 E+09	**24.3**
**Total**				**101**				**790**

4. Purification and Chemical Characterisation of Compounds

4.1 Preparative LCMS Instrument and Programme

The preparative LCMS instrument consisted of a Waters 2767 autosampler, Waters 2545 pump, a Phenomenex Kinetex Axia column (5 µm, C_{18}, 100 Å, 21.2 × 250 mm) equipped with a Phenomenex Security Guard precolumn (Luna, C_5, 300 Å). The system was used at a flow rate of 20 mL/min. Typically, a solvent gradient was run over 15 min starting at 10 % acetonitrile (solvent B, with 0.045 % formic acid) and 90 % HPLC grade water (solvent A; with 0.05 % formic acid) and ramping to 90 % solvent B. The post-column flow was split (100 : 1) and the minority flow was made up with HPLC-grade (1 : 1 solvent A/B) to 1.0 mL/ min for simultaneous analysis by diode array (Waters 2998) in the range 210 to 600 nm, an ELSD detector (Waters 2424) together with a mass spectrometer, Waters SQD-2 mass detector, operating simultaneously in ES^+ and ES^- modes between 150 and 1000 *m/z*. Purification was done mass-directed. Compounds were fractionated and collected into glass test tubes and combined fractions were lyophilized after residual solvent was removed *in vacuo*. Compounds were stored at −20 °C. Mass data was analyzed with MassLynx™ (Waters).

4.2 NMR Instrument Details

Nuclear Magnetic Resonance (NMR) measurements were performed by the NMR department of the Institute for Organic Chemistry, Leibniz University Hannover. NMR data were recorded using either a Bruker Ultrashield 500 (^1H 500 MHz, ^{13}C 125 MHz), Bruker Ascend 600 (^1H 600 MHz, ^{13}C 150 MHz), Bruker Avance III 600 (^1H 600 MHz, ^{13}C 150 MHz), and Bruker Ascend 400 (^1H 400 MHz, ^{13}C 101 MHz) spectrometer. Chemical shifts are shown in parts per million (ppm) and referenced to solvents. The coupling constants J are given in Hz.

4.3 HRMS

High Resolution Mass Spectrometry (HRMS) was performed with a Q-TOF Premier mass spectrometer (Waters) coupled to an Acquity UPLC system (Waters) by the MS department of the Institute of Organic Chemistry, Leibniz University Hannover.

4.4 NMR Characterisation of Isolated Compounds

4.4.1 6-*E*-3,12-Dihydroxy-3,7,11-trimethyltetradec-6-enedioic acid **13**

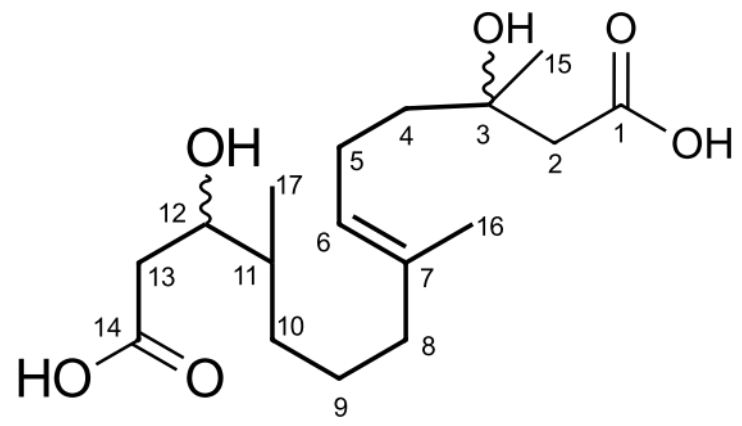

Pos.	Measured in DMSO-d6, 298K*			
	δ_C 151 MHz	δ_H (*J*/Hz) 500 MHz	COSY	HMBC (H to C)
**1**	173.1	-	-	-
**2**	46.3	a 2.28, d, (13.9)	2b	1, 3, 4, 15
		b 2.30, d, (13.9)	2a	1, 3, 4, 15
**3**	69.9	-	-	-
**4**	41.5	1.45, m, overlapped	5	-
**5**	22.1	1.99, m	4, 6	-
**6**	124.5	5.08, t (7.0)	5	4, 5, 8, 16
**7**	134.3	-	-	-
**8**	39.3	1.90, m	9a, 9b	-
**9**	25.0	a 1.25, m	8	-
		b 1.41, m	8	-
**10**	31.1	a 0.99, m	10b, 11	-
		b 1.33, m	10a, 11	-
**11**	38.2	1.44, m, overlapped	12(w), 17	-
**12**	71.0	3.70, ddd (9.4, 4.9, 3.5)	11(w), 13a, 13b	-
**13**	38.9	a 2.12, dd (14.5, 9.4)	12, 13b	12, 14
		b 2.26 m, overlapped	13a	12, 14
**14**	173.8	-	-	-
**15**	27.0	1.15, s	-	2, 3, 4
**16**	15.6	1.54, s	-	6, 7, 8
**17**	15.2	0.79, d (6.9)	11	10, 11, 12

* note that ^{1}H and ^{13}C spectra were obtained on different instruments.

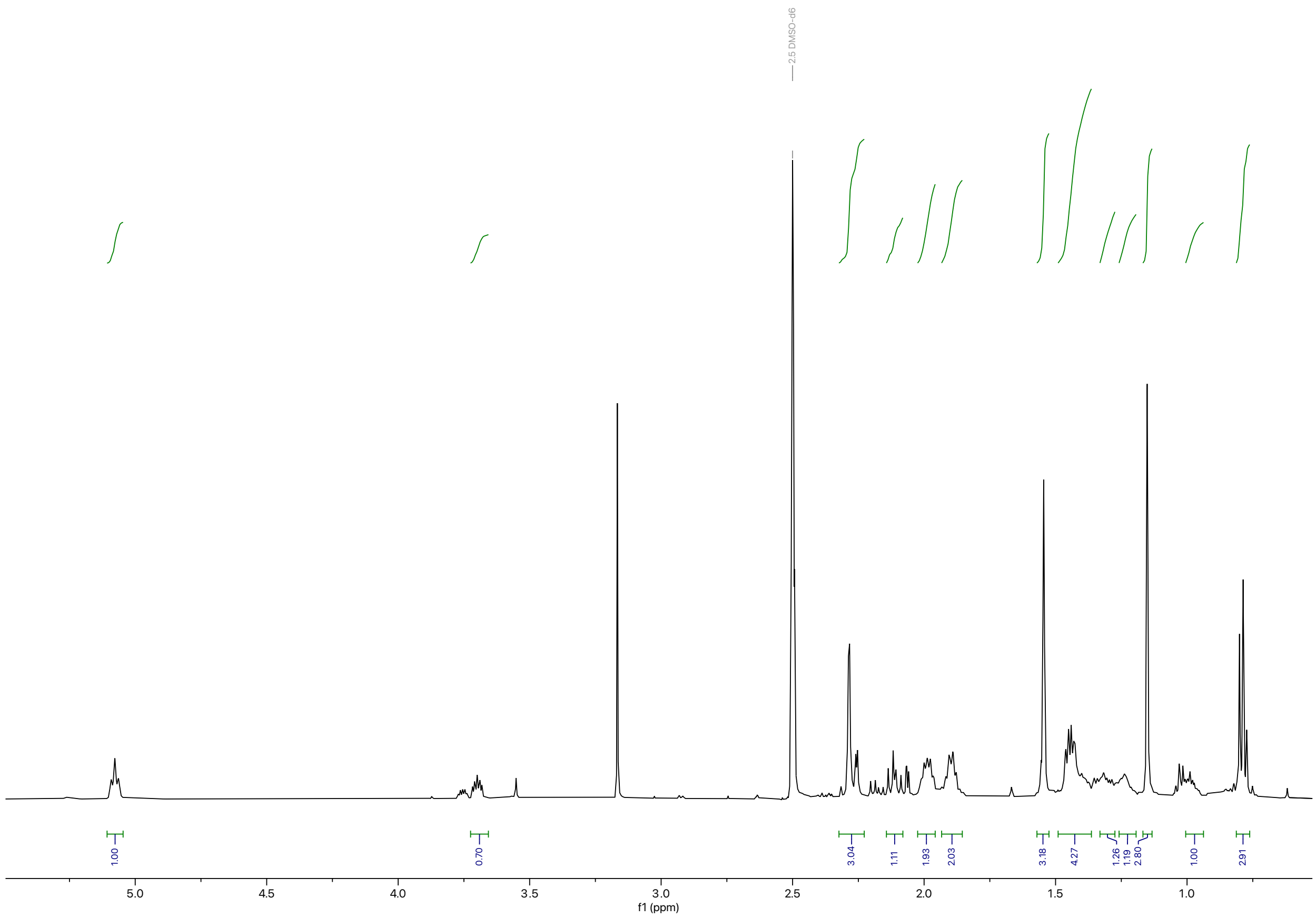
2.5 DMSO-d6
1.00
0.70
3.04
1.11
1.93
2.03
3.18
4.27
1.26
1.19
2.80
1.00
2.91
5.0
4.5
4.0
3.5
3.0
2.5
2.0
1.5
1.0
f1 (ppm)

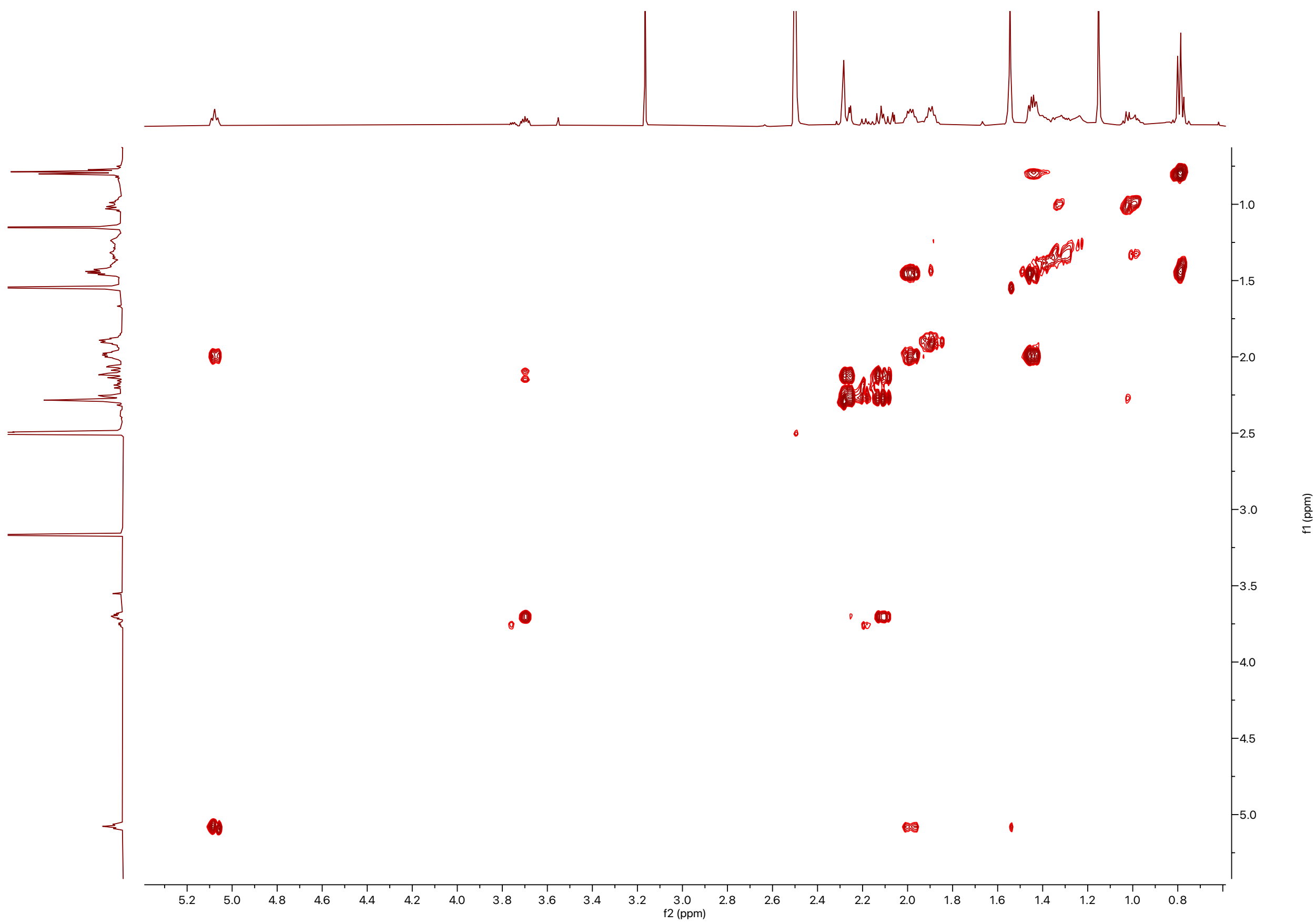
5.2
5.0
4.8
4.6
4.4
4.2
4.0
3.8
3.6
3.4
3.2
3.0
2.8
2.6
2.4
2.2
2.0
1.8
1.6
1.4
1.2
1.0
0.8
f2 (ppm)
f1 (ppm)

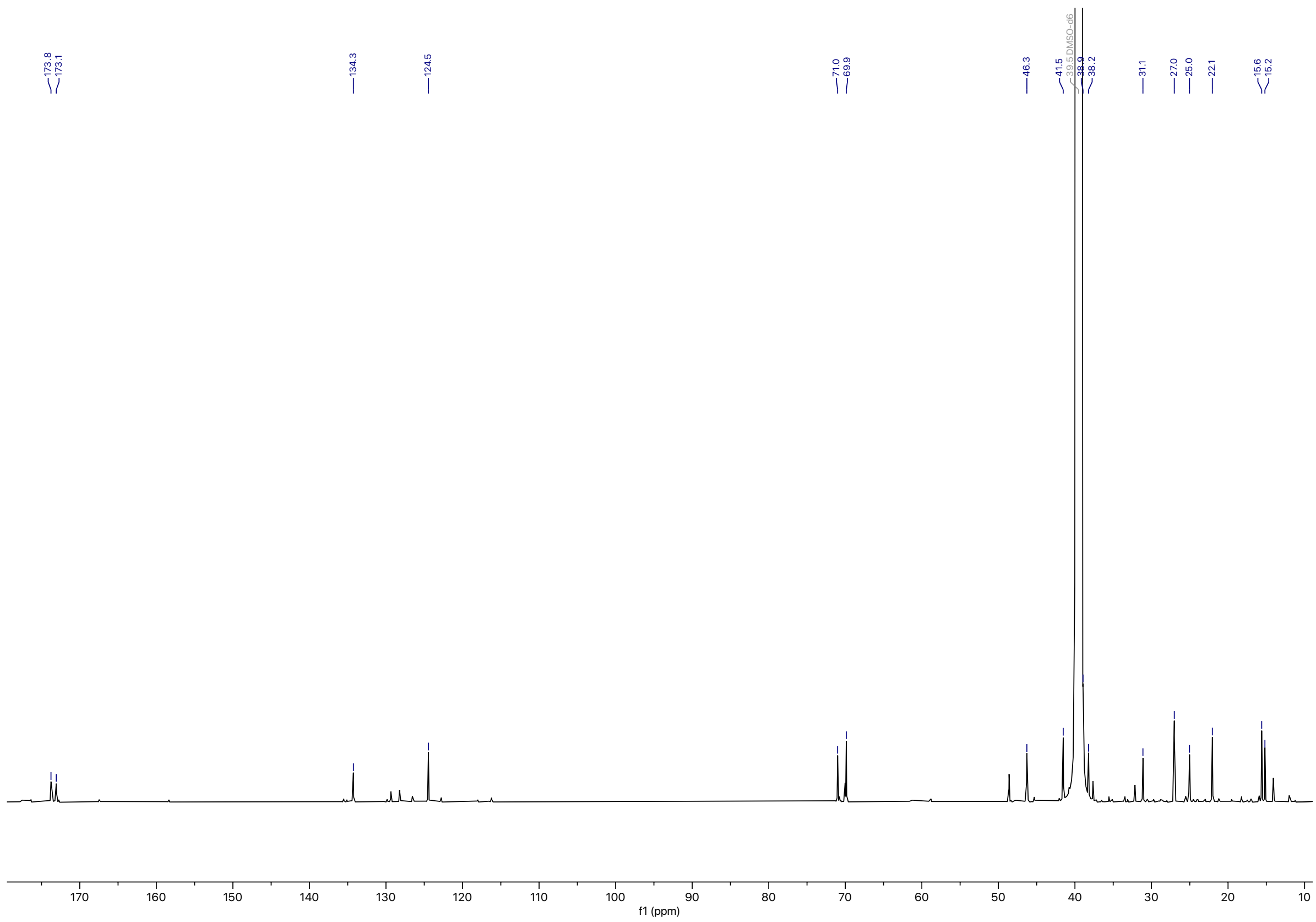
173.8
173.1
134.3
124.5
71.0
69.9
46.3
41.5
38.9
38.2
31.1
27.0
25.0
22.1
15.6
15.2
f1 (ppm)

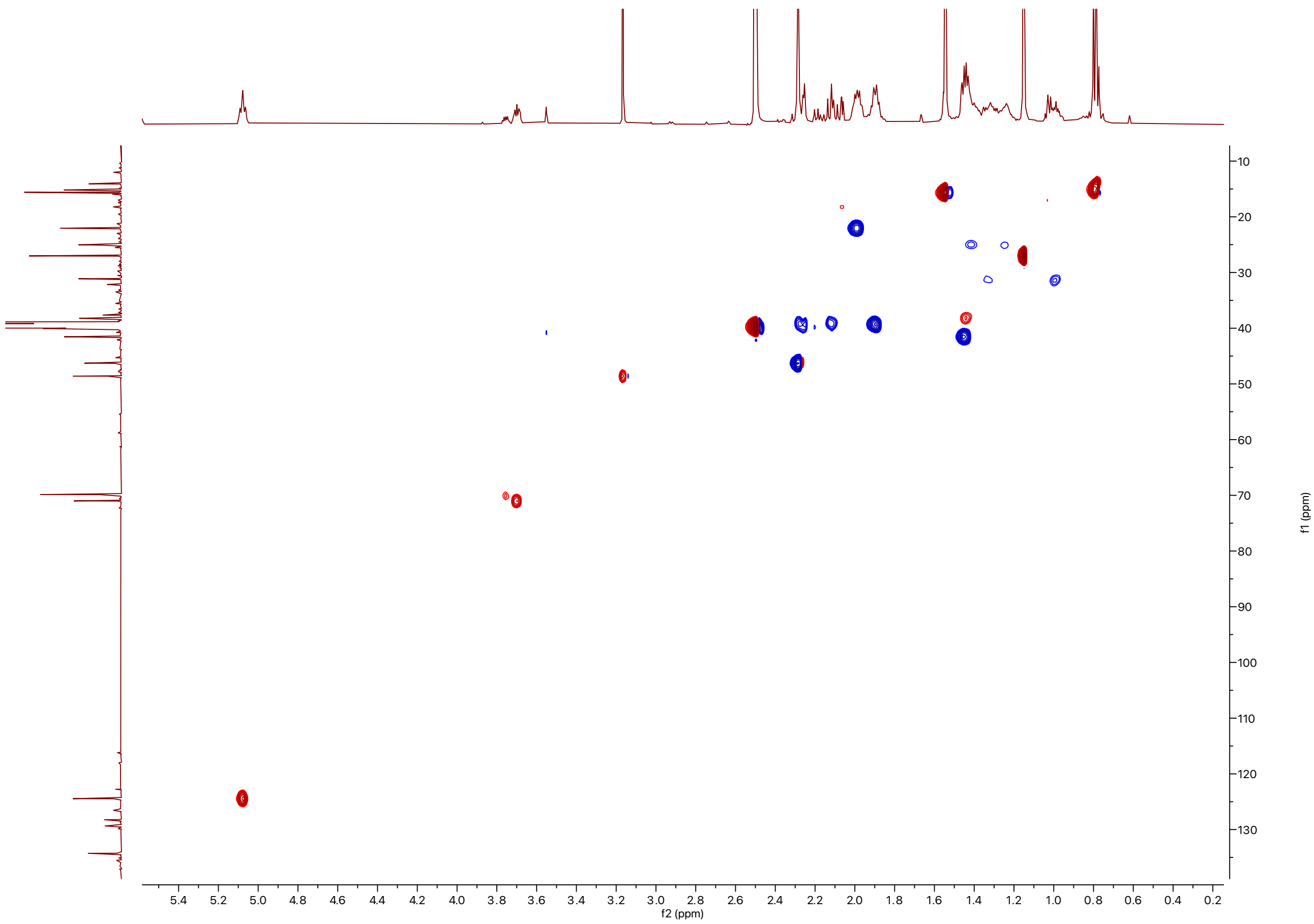
f2 (ppm)
f1 (ppm)

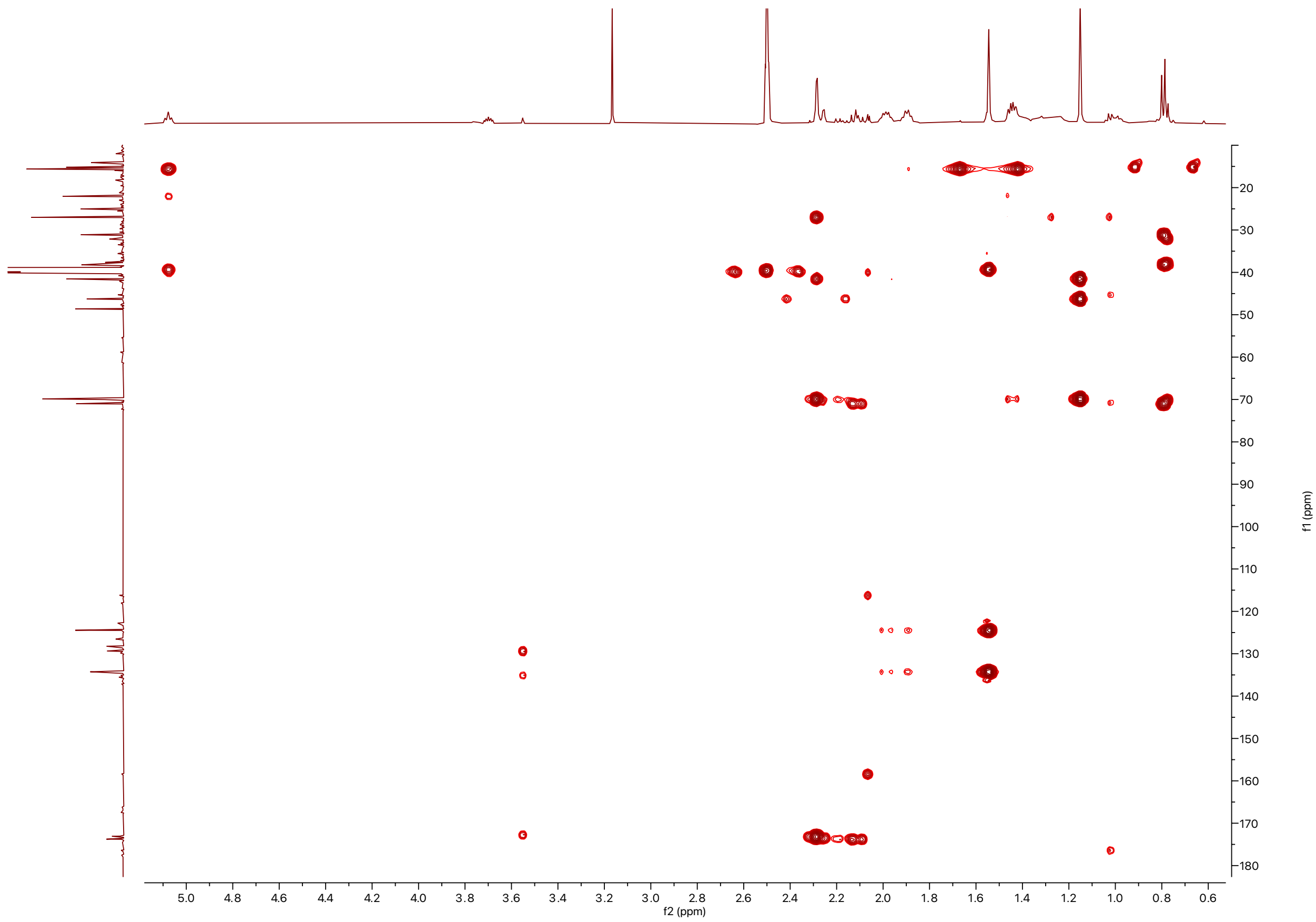
f1 (ppm)
f2 (ppm)

4.4.2 4-*E*,8-*E*-12-Hydroxy-4,8,12-trimethyltetradeca-4,8-dienedioic acid **14**

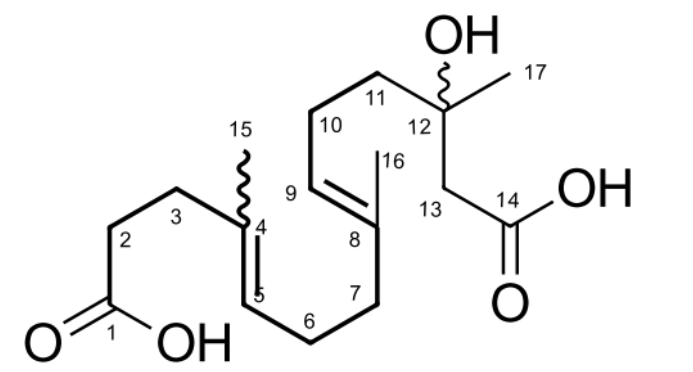

Pos.	Measured in DMSO-d$_6$, 298 K			
	δ$_C$, 126 MHz	δ$_H$ (*J*/Hz), 500 MHz	COSY	HMBC (H to C)
**1**	174.2	-	-	-
**2**	32.7	2.26, overlapped	3	-
**3**	34.2	2.16, t (7.7)	2	1, 2, 4, 5, 15
**4**	133.6	-	-	-
**5**	124.1	5.09, overlapped	6	-
**6**	26.0	2.02, overlapped	5, 7	-
**7**	39.1	1.92, t (8.1)	6	5, 6, 8, 9, 16
**8**	133.9	-	-	-
**9**	124.7	5.09, overlapped	10	-
**10**	22.1	2.00, overlapped	9, 11	-
**11**	41.5	1.45, m	10	-
**12**	69.9	-	-	-
**13**	46.3	2.29, overlapped	-	-
**14**	173.1	-	-	-
**15**	15.8	1.56, s, overlapped	-	-
**16**	15.7	1.56, s, overlapped	-	-
**17**	27.0	1.16, s	-	11, 12, 13

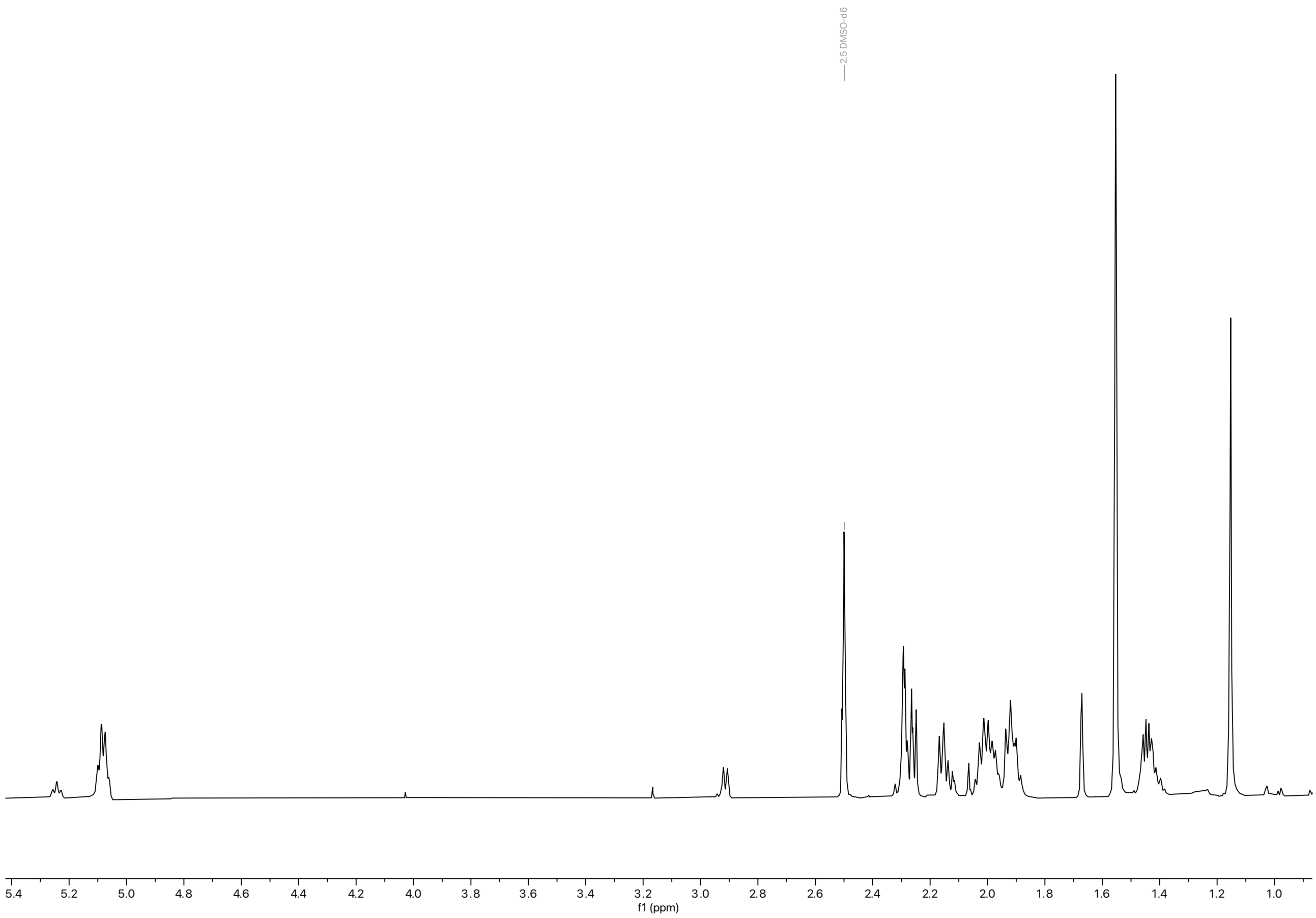

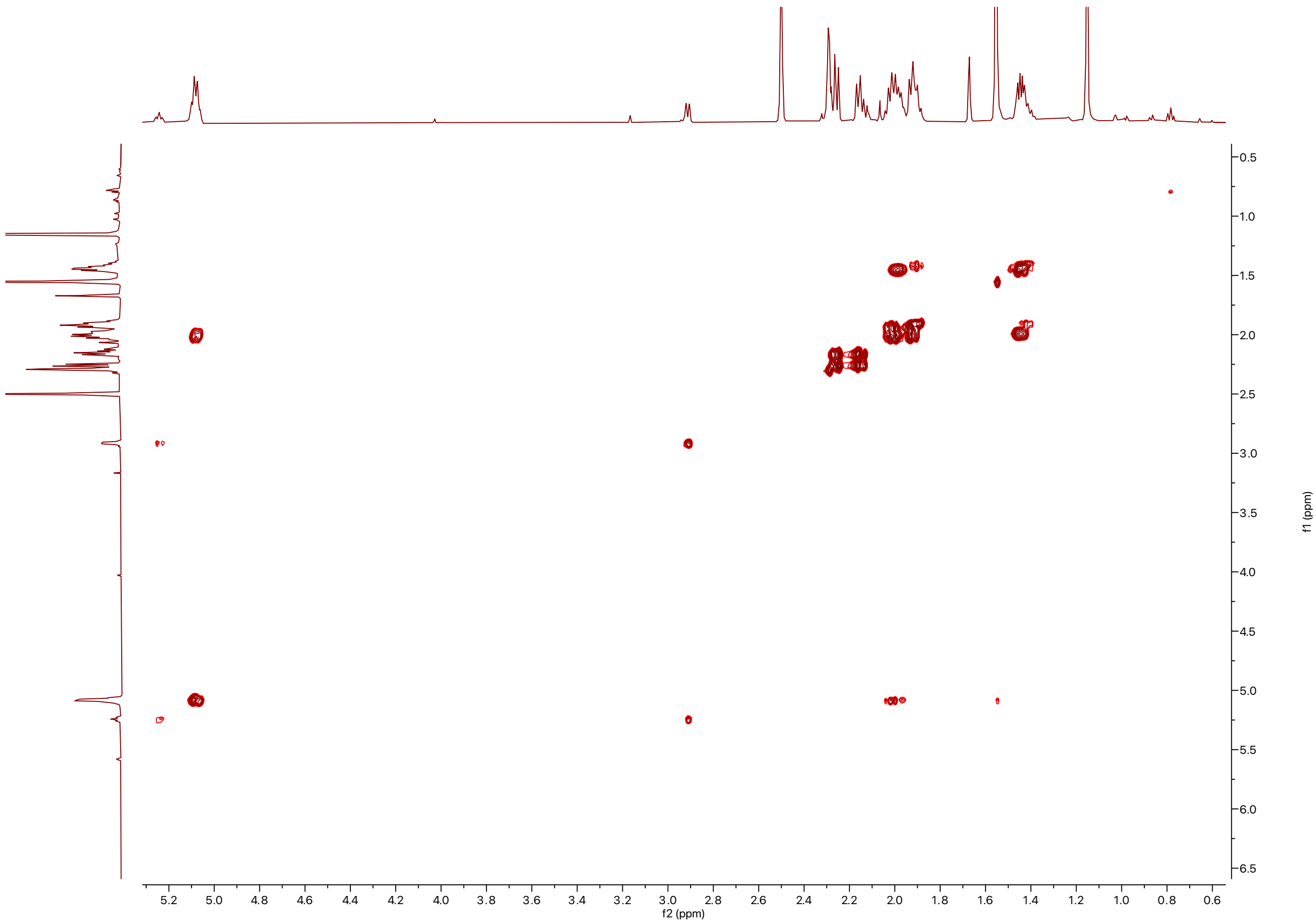
f2 (ppm)
f1 (ppm)

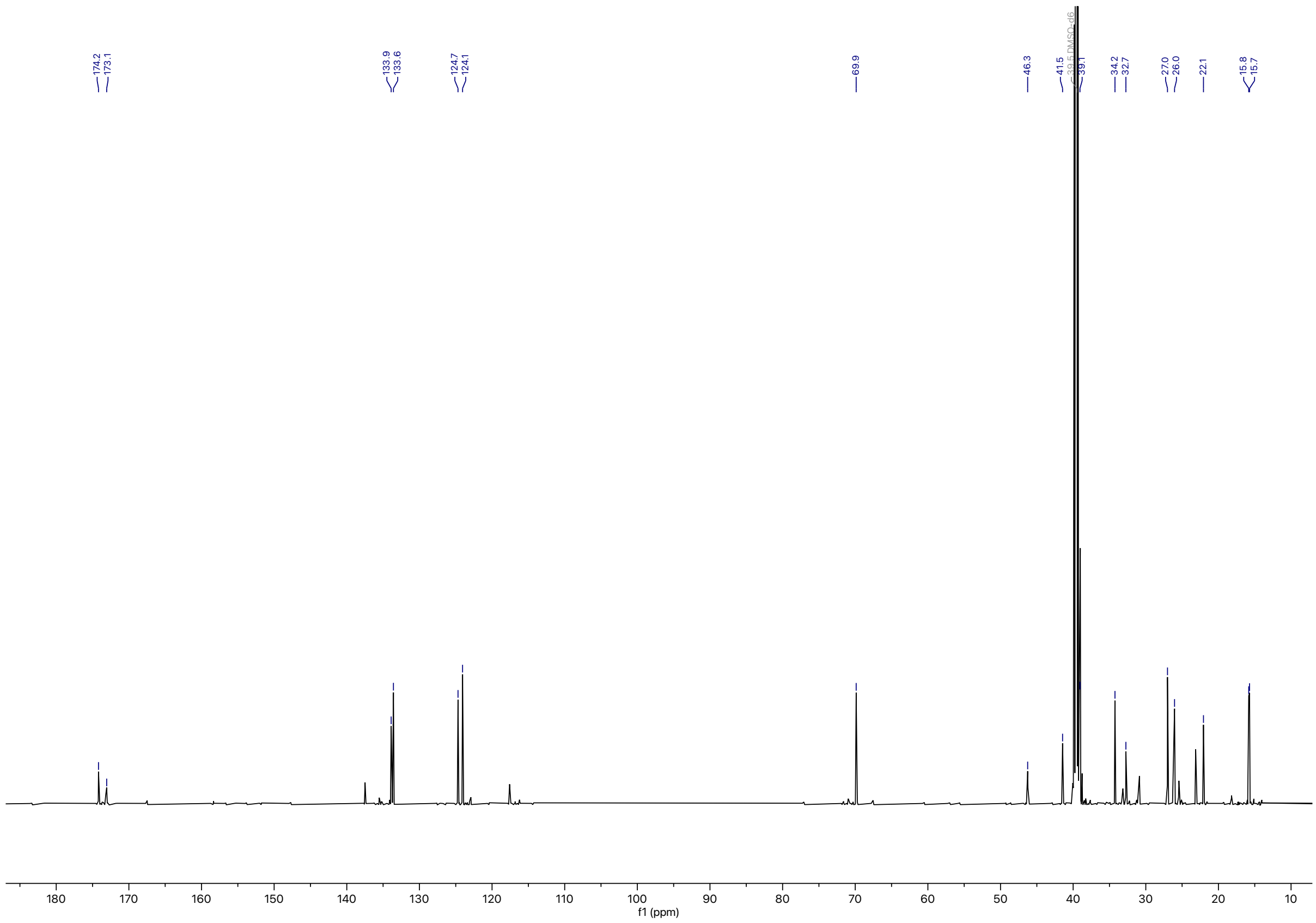
174.2
173.1
133.9
133.6
124.7
124.1
69.9
46.3
41.5
39.1
34.2
32.7
27.0
26.0
22.1
15.8
15.7
f1 (ppm)

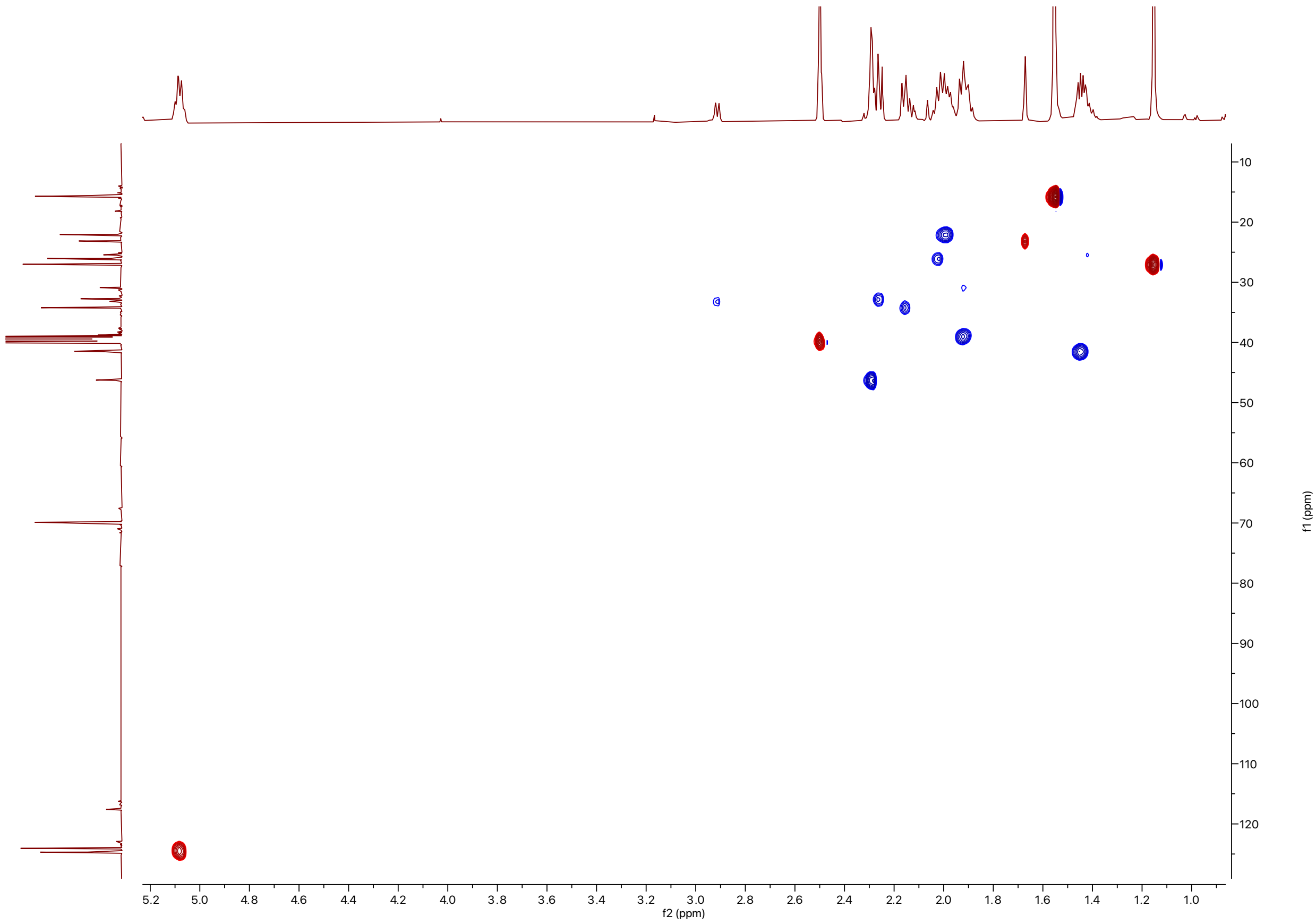
f2 (ppm)
f1 (ppm)

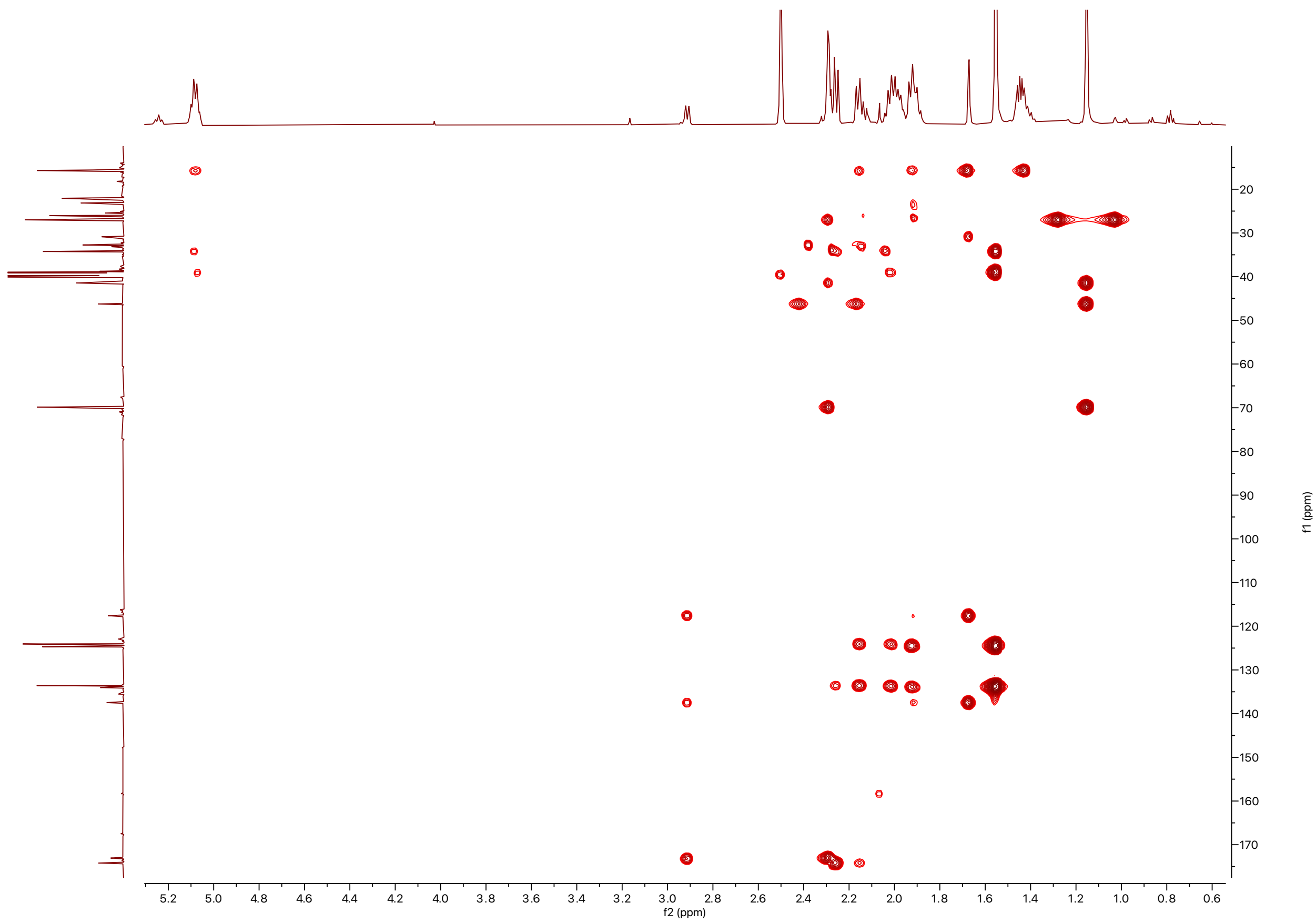
f2 (ppm)
f1 (ppm)

4.4.3 6-*E*-10-Hydroxy-2,6,10-trimethyldodec-6-enedioic acid **15**

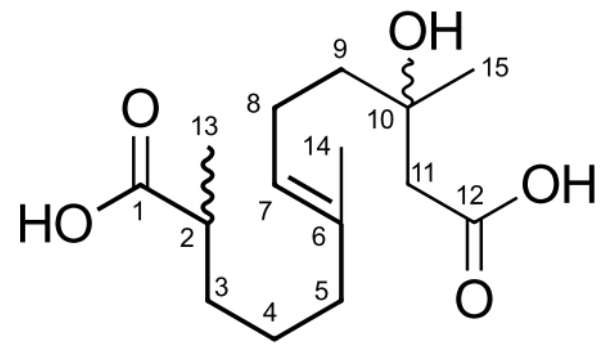

**Pos.**	**Measured in DMSO-d₆, 298 K**			
	δ_C, 126 MHz	δ_H (*J*/Hz), 500 MHz	COSY	HMBC (H to C)
**1**	177.5	-	-	-
**2**	38.6	2.31, m	13	1, 3, 13
**3**	32.9	a 1.28, m	-	1, 5
		b 1.49, m	-	2
**4**	24.9	1.33, m	-	2, 6
**5**	38.9	1.91, t (7.4)	-	3, 4, 6, 7, 14
**6**	134.0	-	-	-
**7**	124.7	5.07, m	8	5, 8(w), 14
**8**	22.1	1.98, m	7, 9	6, 7, 9
**9**	41.6	1.44, m	8	8, 10, 11, 15
**10**	69.8	-	-	-
**11**	46.3	2.26, overlapped	-	12
**12**	173.2	-	-	-
**13**	17.0	1.03, d (6.9)	2	1, 2, 3
**14**	15.5	1.53, brs	-	5, 6, 7
**15**	27.0	1.14, s	-	9, 10, 11

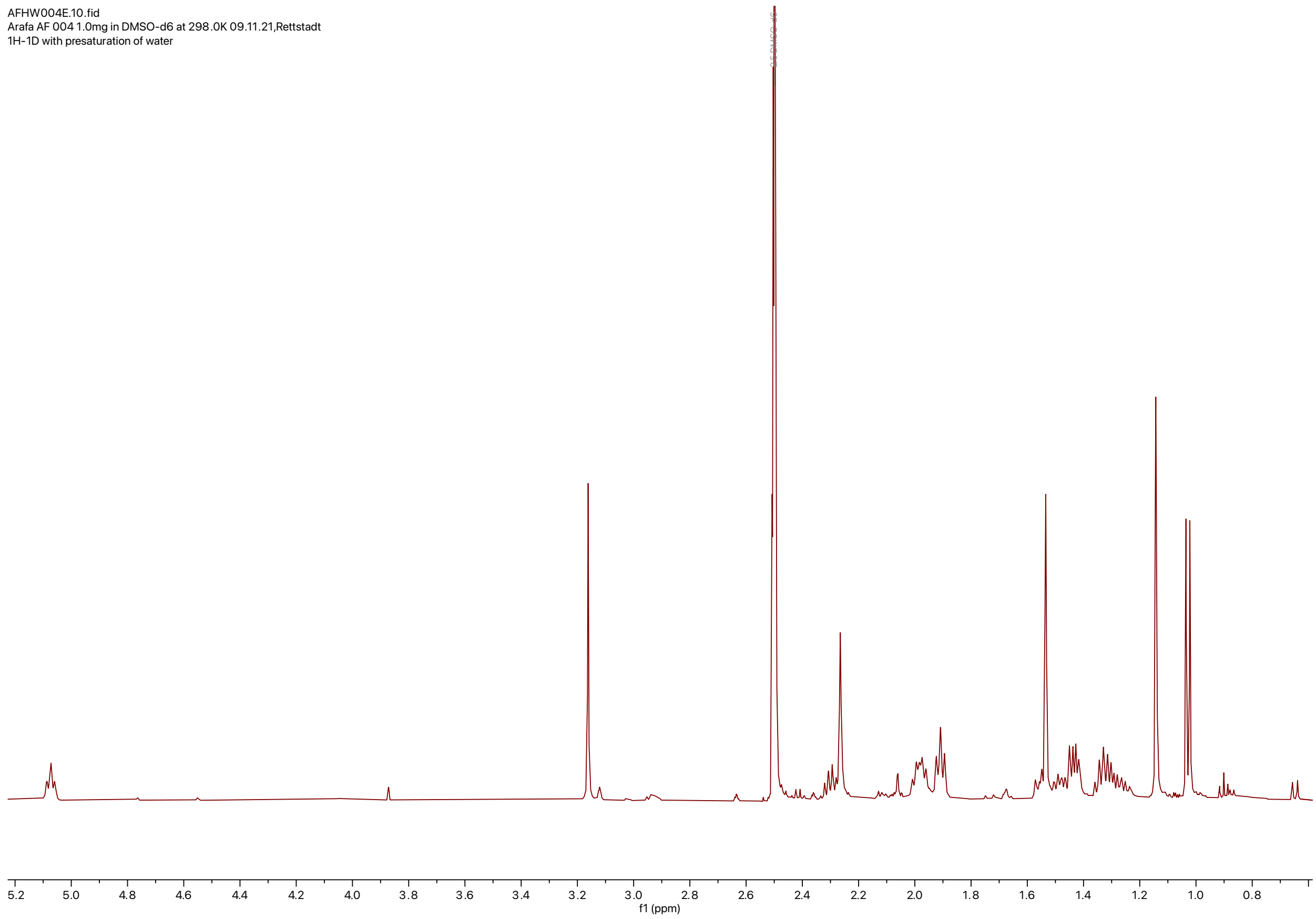

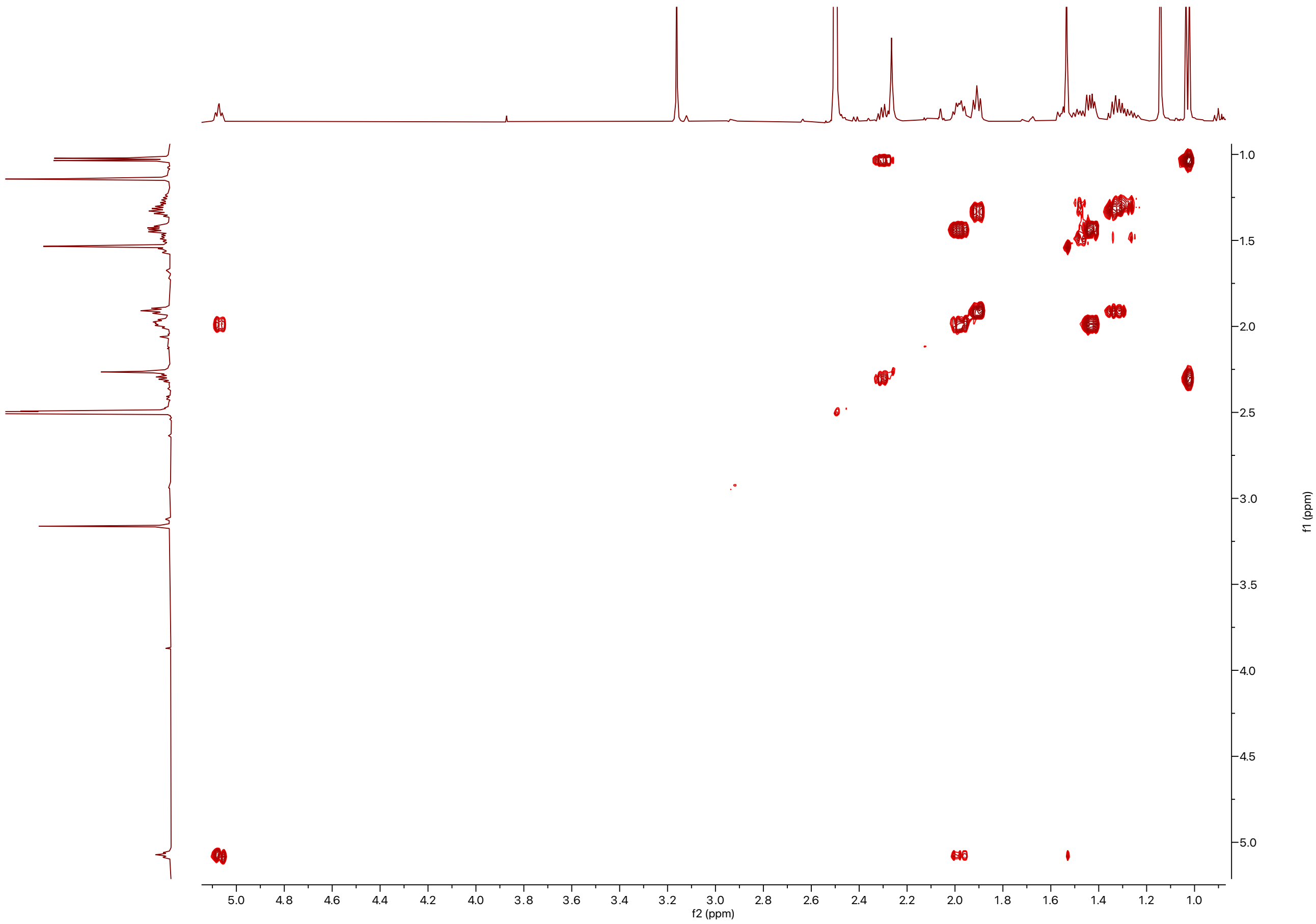
f2 (ppm)
f1 (ppm)

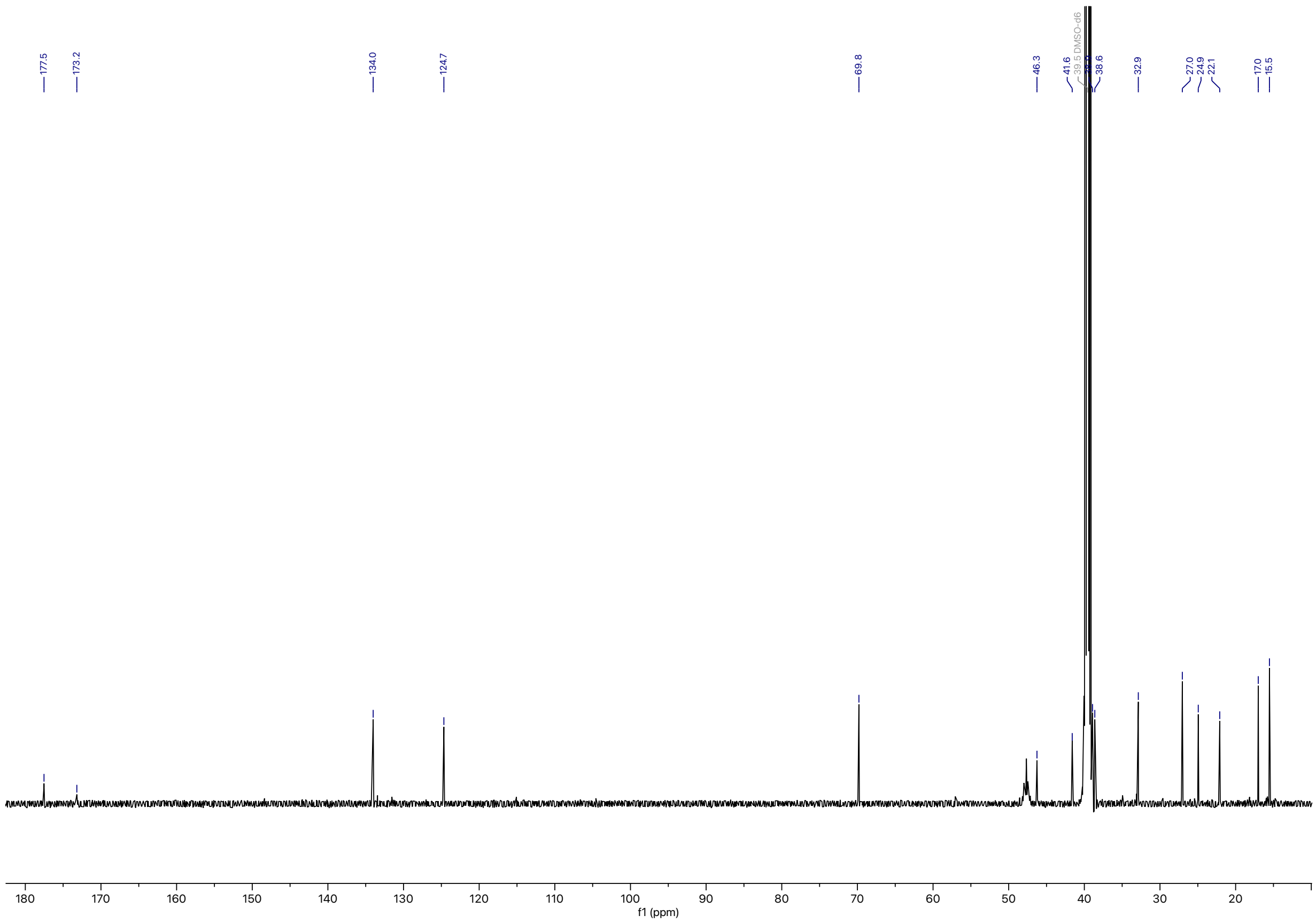
177.5
173.2
134.0
124.7
69.8
46.3
41.6
39.5 DMSO-d6
38.6
32.9
27.0
24.9
22.1
17.0
15.5
f1 (ppm)

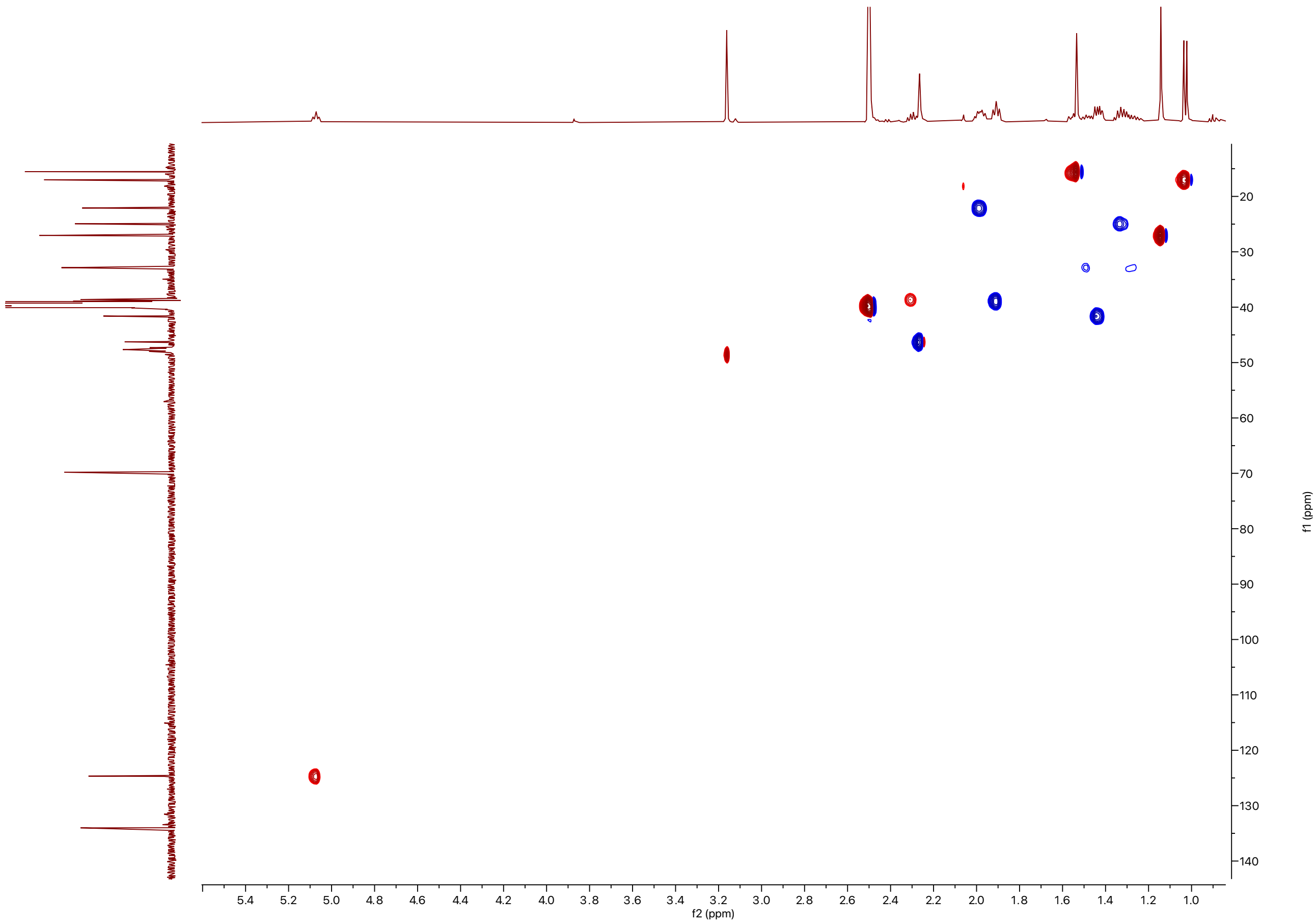
f2 (ppm)
f1 (ppm)

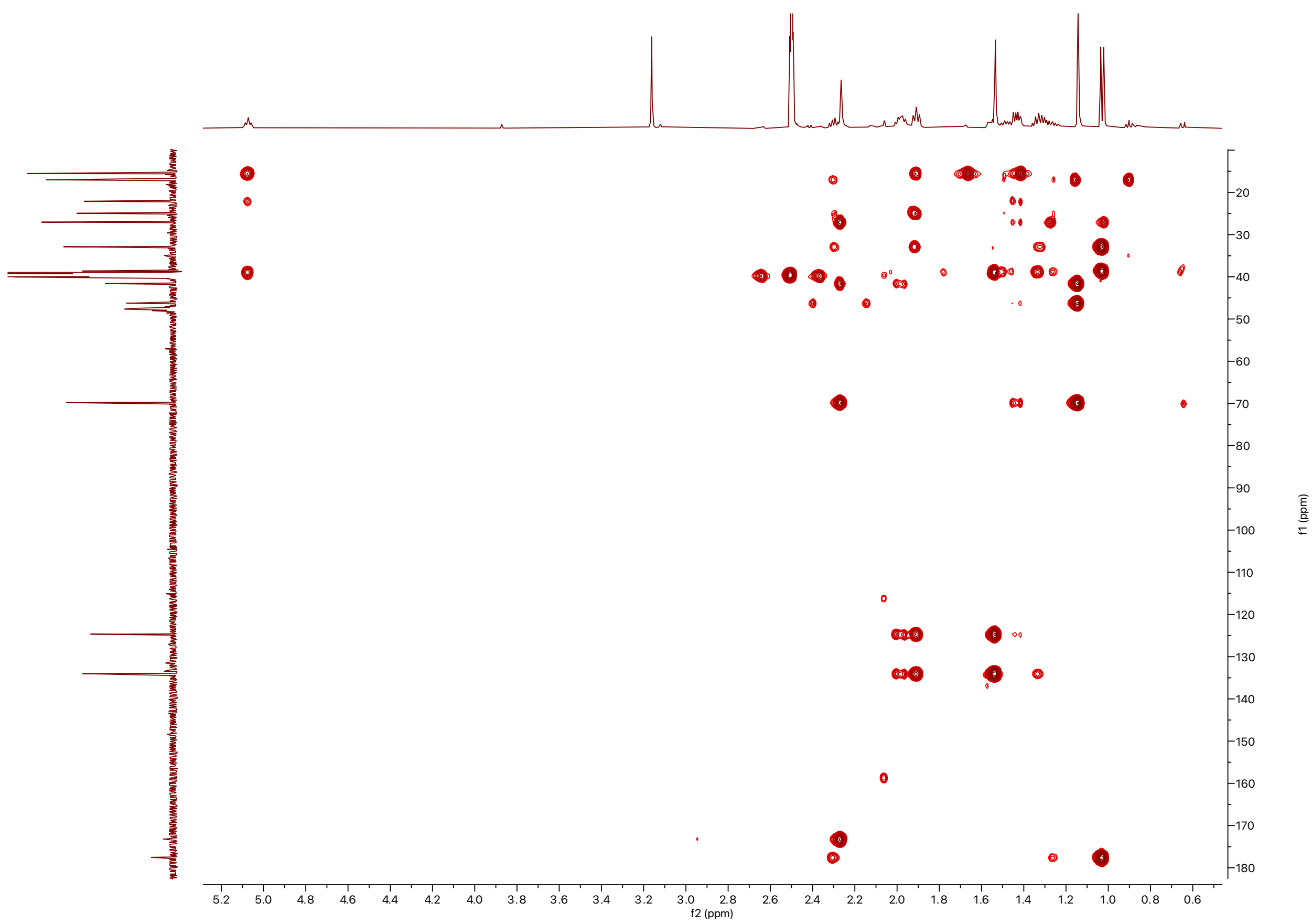
f2 (ppm)
f1 (ppm)

4.4.4 3α,13,18-Trihydroxy-*ent*-labd-8(17)-en-15-oic acid **16**

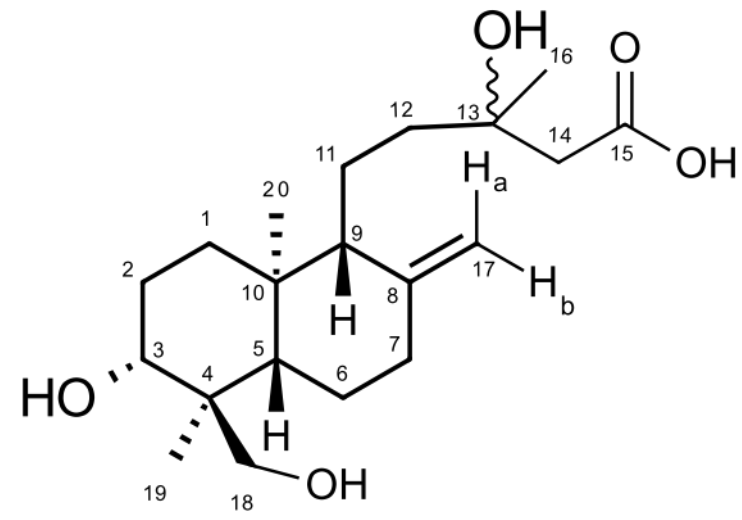

Pos.	Measured in DMSO-d$_6$, 298K			
	δ$_C$, 151 MHz	δ$_H$ (*J*/Hz), 600 MHz	COSY	HMBC (H to C)
**1**	36.6	a 1.05, ddd (13.2, 13.2, 3.6)	1b, 2	2, 3, 9, 10, 20
		b 1.70, ddd (13.2, 3.4, 3.4)	1a	3, 5, 10,
**2**	27.3	1.52, overlapped	-	-
**3**	70.2	3.47, dd (11.5, 4.8)	2	4, 18, 19
**4**	42.3	-	-	-
**5**	45.8	1.46, overlapped	-	-
**6**	23.2	a 1.22, m	5, 6b, 7a, 7b	5, 7, 10
		b 1.60, overlapped	-	-
**7**	37.6	a 1.92, ddd (13.0, 12.5, 4.1)	7b, 6a, 6b	6, 8, 17
		b 2.28, ddd (12.5, 3.0, 3.0)	7a, 6a	5, 6, 8, 9, 17
**8**	148.2	-	-	-
**9**	56.8	1.46, overlapped	-	-
**10**	39.0	-	-	-
**11**	17.4	a 1.31, m	9, 11b, 12b	8, 12, 13
		b 1.52, overlapped	-	-
**12**	40.7	a 1.12, overlapped	-	-
		b 1.60, overlapped	-	-
**13**	70.2	-		
**14**	46.4	a 2.23, overlapped	-	-
		b 2.23, overlapped	-	-
**15**	173.4	-	-	-
**16**	27.2	1.12, s	-	12, 13, 14
**17**	106.6	a 4.55, s	17b	7, 8, 9
		b 4.77, s	17a	7, 8, 9
**18**	64.3	a 3.04, d (10.6)	18b	3, 4, 5, 19
		b 3.32, d (10.6)	18a	3, 4, 5, 19
**19**	12.4	0.52, s	-	3, 4, 5, 18
**20**	14.7	0.64, s	-	1, 5, 9, 10

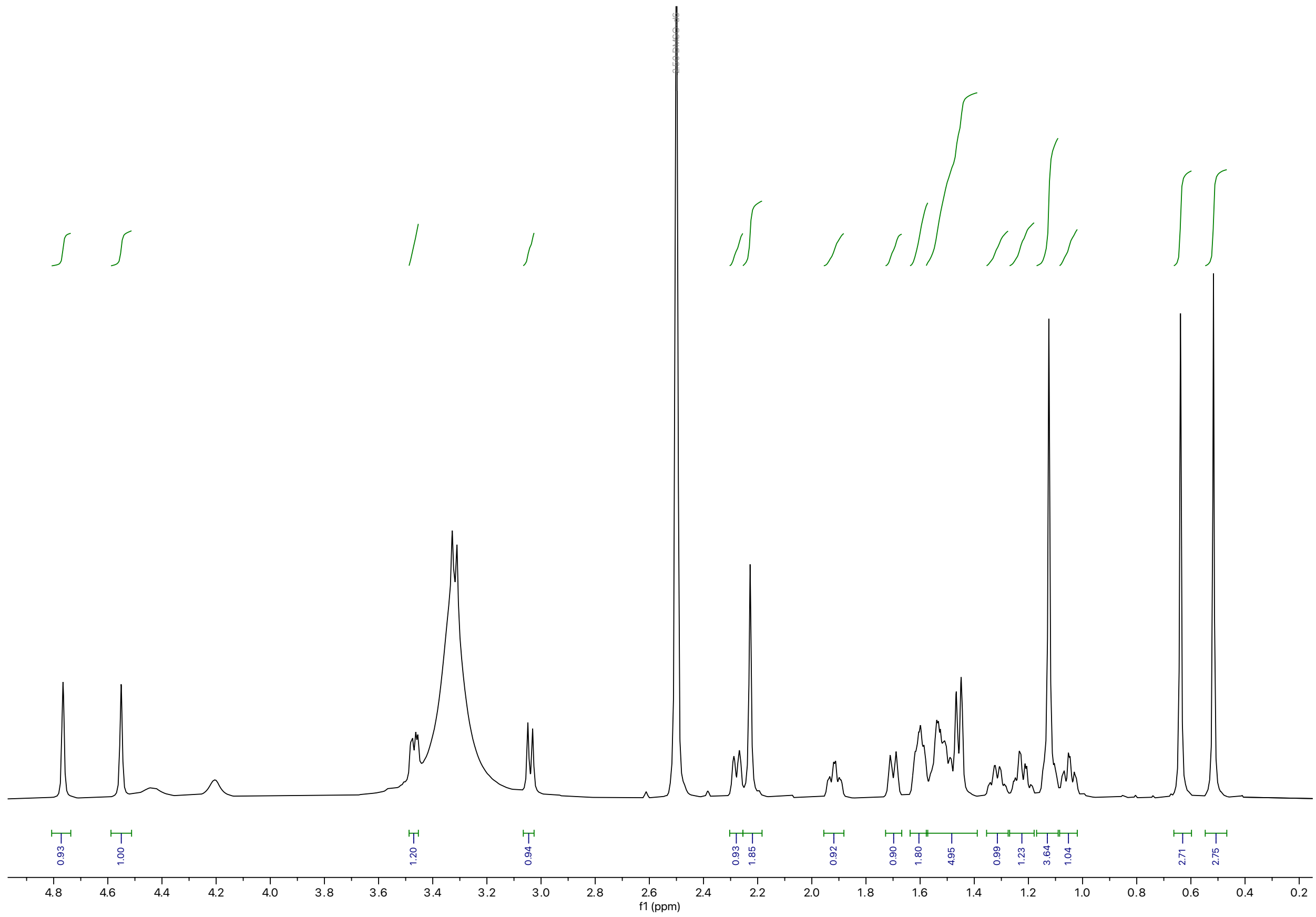
0.93
1.00
1.20
0.94
0.93
1.85
0.92
0.90
1.80
4.95
0.99
1.23
3.64
1.04
2.71
2.75
f1 (ppm)

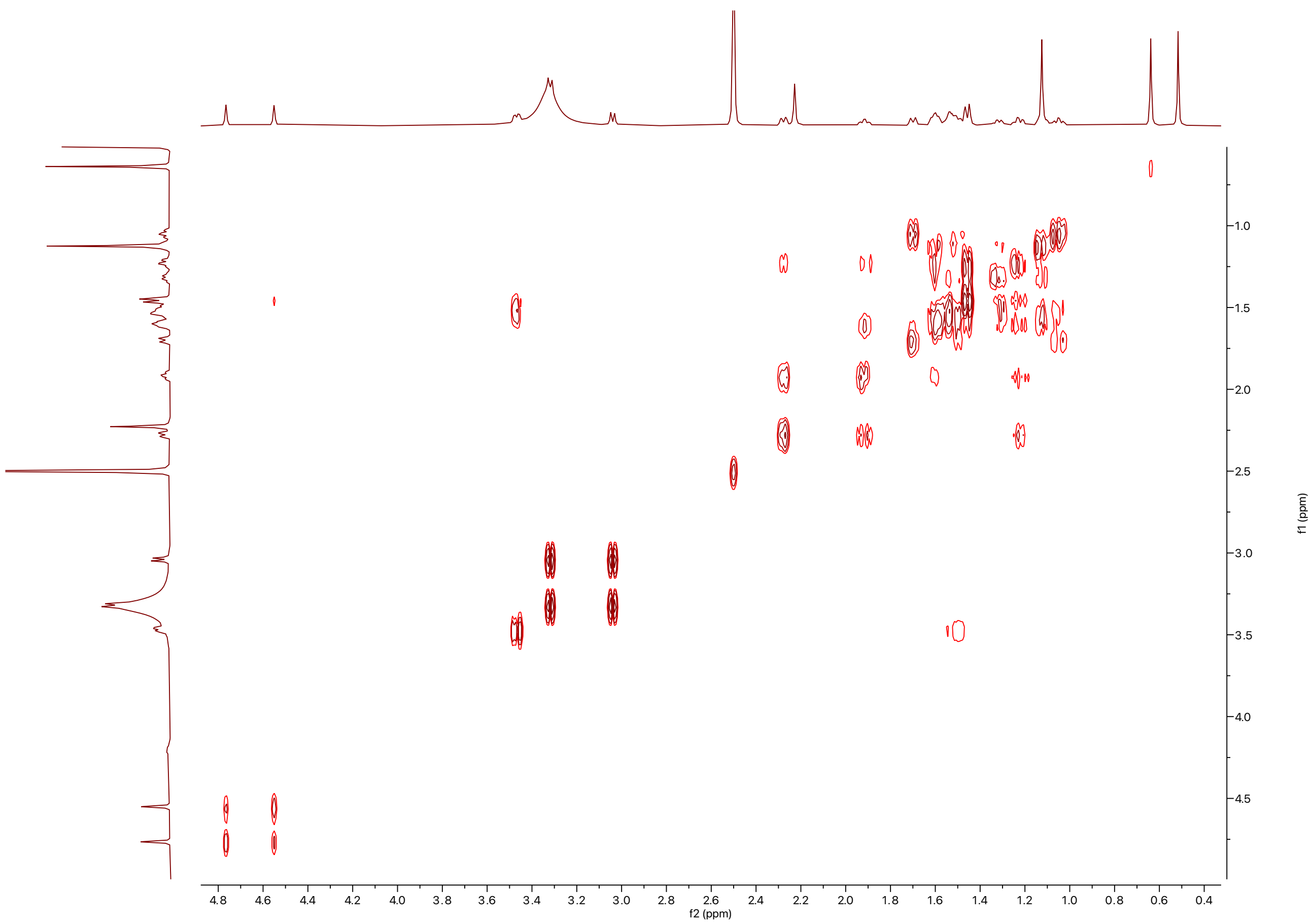
f2 (ppm)
f1 (ppm)

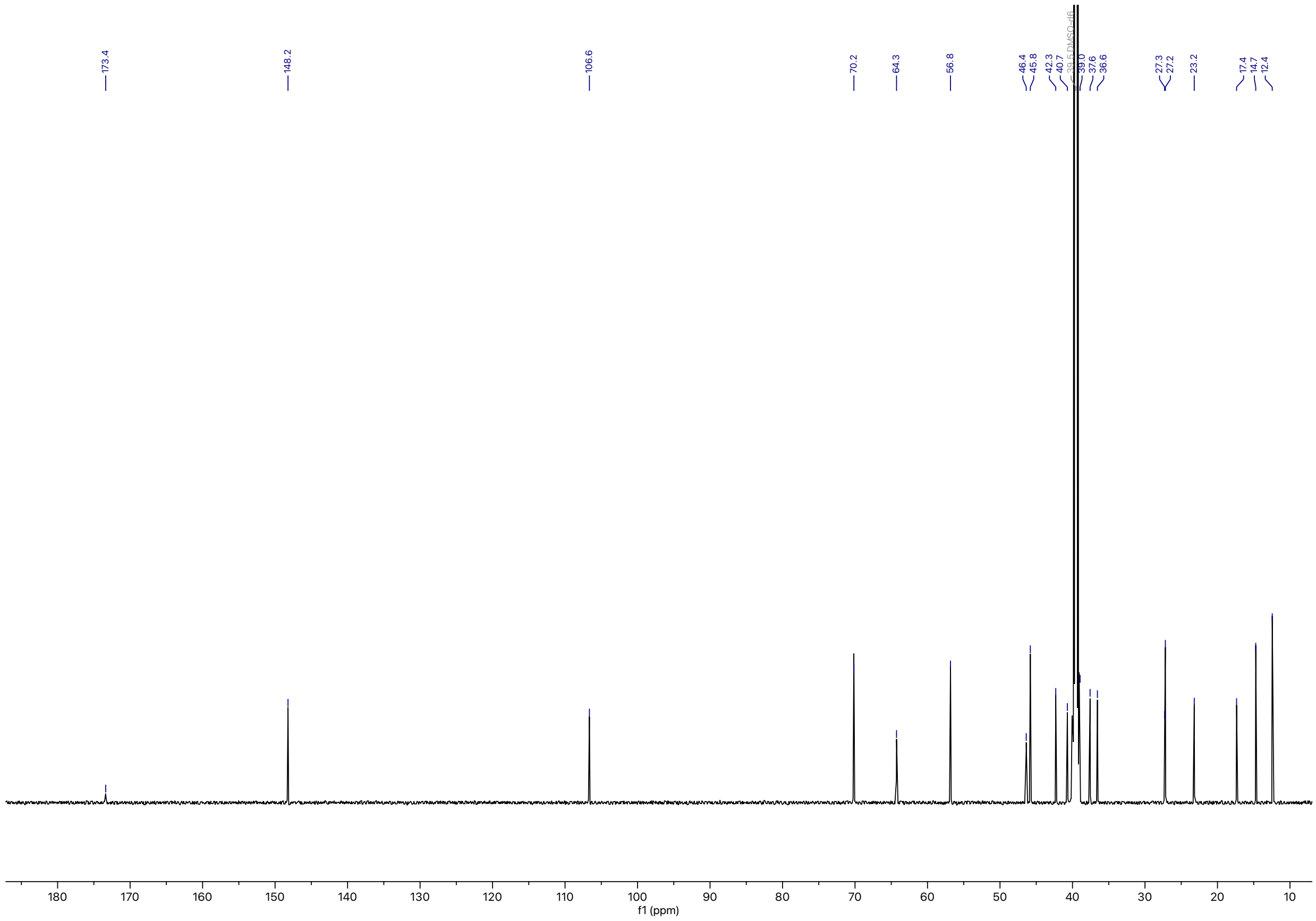
173.4
148.2
106.6
70.2
64.3
56.8
46.4
45.8
42.3
40.7
37.6
36.6
27.3
27.2
23.2
17.4
14.7
12.4
180
170
160
150
140
130
120
110
100
90
80
70
60
50
40
30
20
10
f1 (ppm)

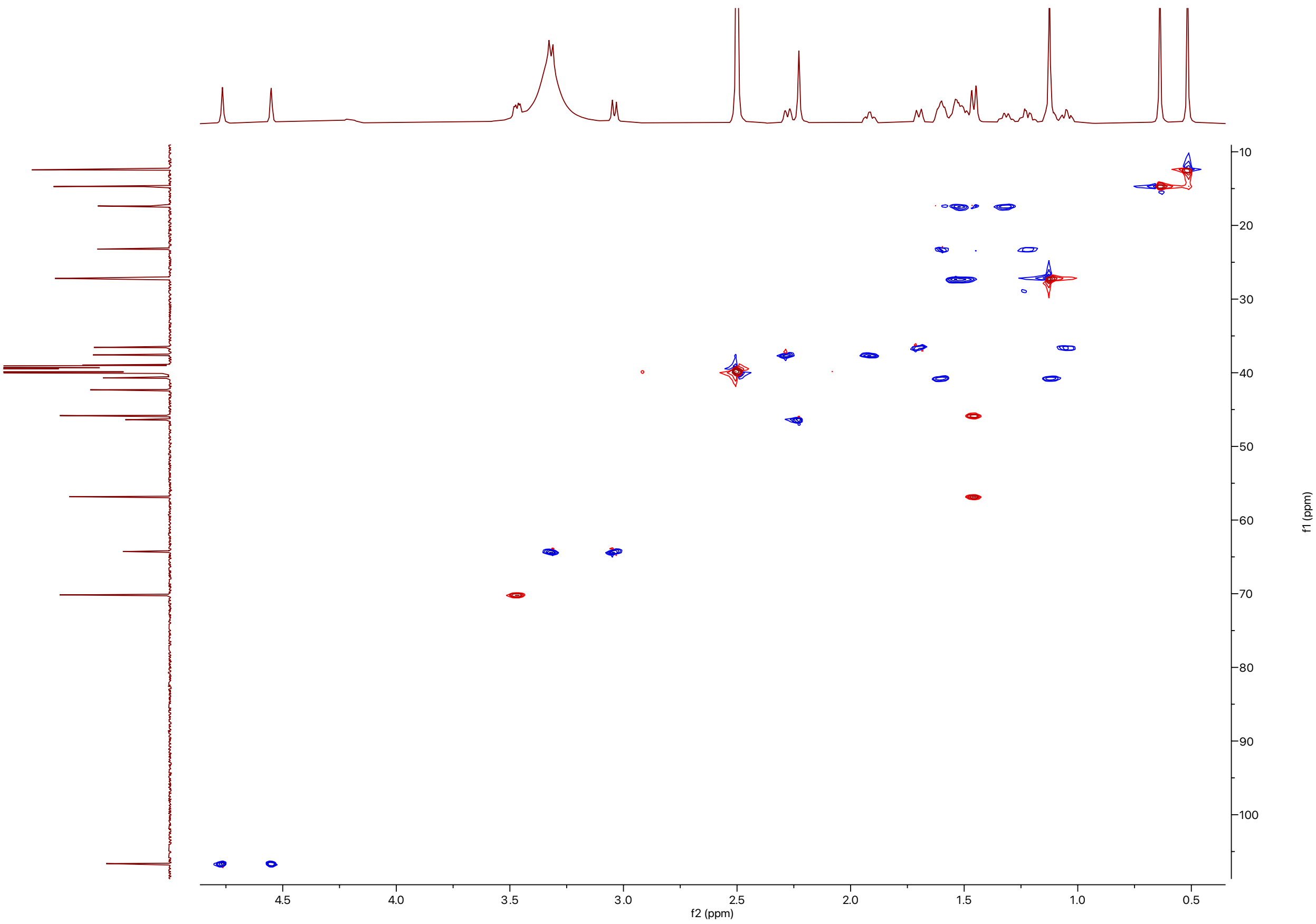
4.5
4.0
3.5
3.0
2.5
2.0
1.5
1.0
0.5
f2 (ppm)
10
20
30
40
50
60
70
80
90
100
f1 (ppm)

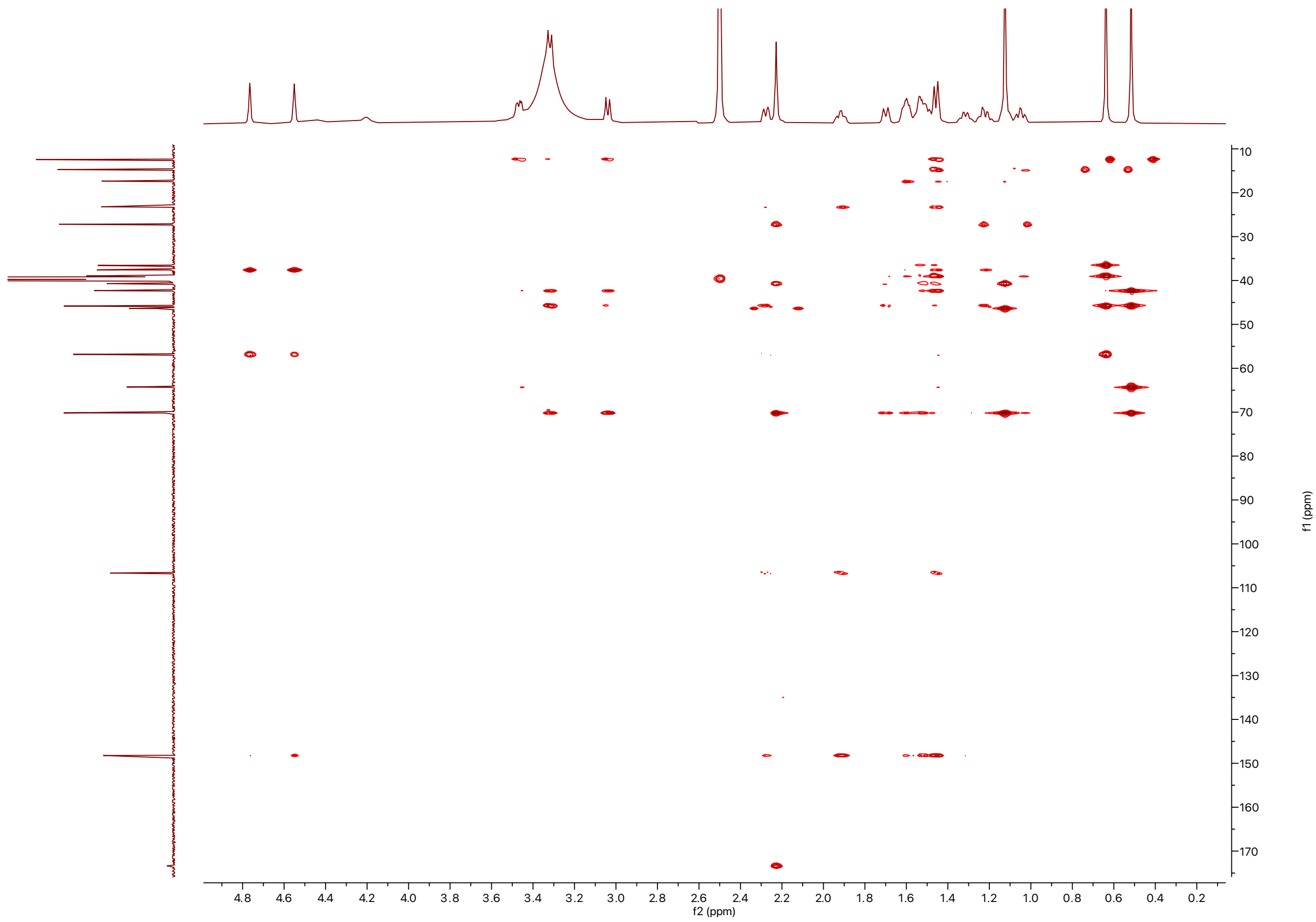
f2 (ppm)
f1 (ppm)

4.4.5 13-Hydroxy-*ent*-labd-8(17)-en-15,18-dioic acid **17**

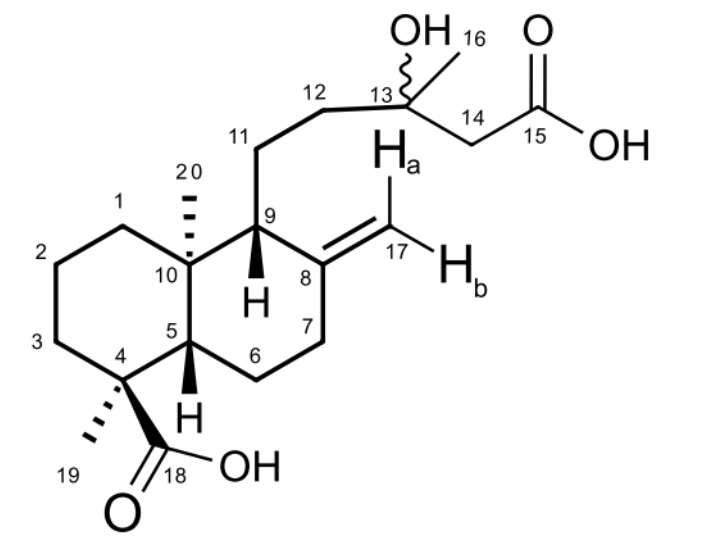

**Pos.**	Measured in DMSO-d6, 298K			
	δ_C, 151 MHz	δ_H (*J*/Hz), 600 MHz	COSY	HMBC (H to C)
**1**	37.8	a 1.06, overlapped	1b	-
		b 1.76, brd (13.0)	1a	3
**2**	18.1	1.53, overlapped	3b	-
**3**	36.6	a 1.74, brd (13.0)	-	5
		b 1.68, m	2	-
**4**	46.5	-	-	-
**5**	49.3	1.86, brd (12.1)	6b	4, 6, 7, 19, 20
**6**	26.3	a 1.24, m	6b	-
		b 1.36, m	5, 6a, 7b	-
**7**	37.4	a 1.92, ddd (13.2, 12.7, 5.0)	7b	8, 17
		b 2.29, brd (12.7)	6b, 7a	5, 8, 17
**8**	147.7	-	-	-
**9**	57.0	1.54, overlapped	17a	-
**10**	38.7	-	-	-
**11**	17.1	a 1.33, m	11b	8,9
		b 1.53, overlapped	11a	-
**12**	40.9	a 1.10, overlapped	12b	-
		b 1.56, overlapped	12a	-
**13**	69.9	-	-	-
**14**	46.9	2.13, m	-	15
**15**	174.2	-	-	-
**16**	27.3	1.09, s	-	12, 13, 14
**17**	107.2	a 4.58, brs	9, 17b	7, 9
		b 4.78, brs	17a	7, 9
**18**	179.6	-	-	-
**19**	16.6	1.02, s	-	3, 4, 5, 18
**20**	14.5	0.65, s	-	1, 5, 9, 10

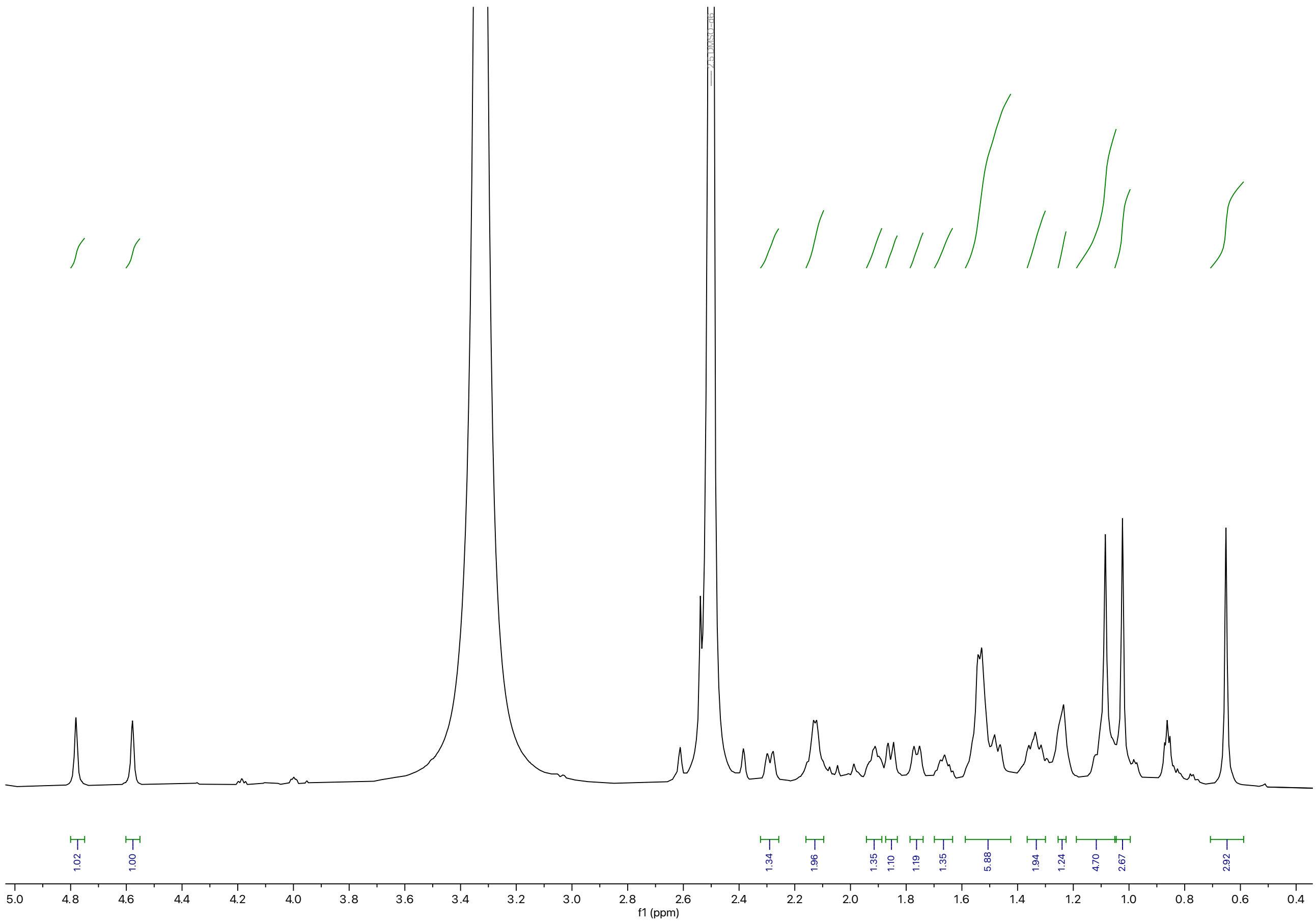
1.02
1.00
1.34
1.96
1.35
1.10
1.19
1.35
5.88
1.94
1.24
4.70
2.67
2.92
f1 (ppm)

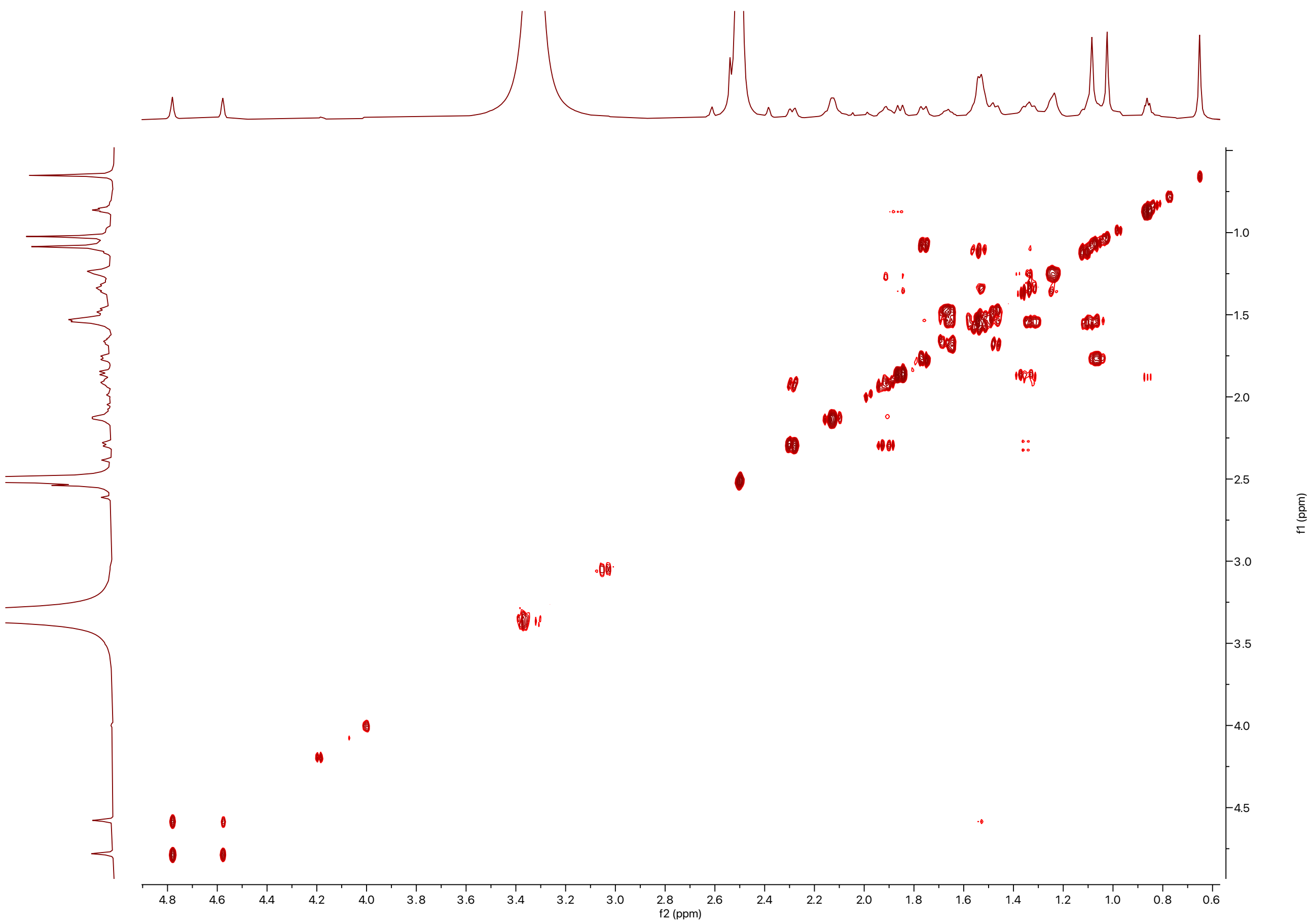
f2 (ppm)
f1 (ppm)

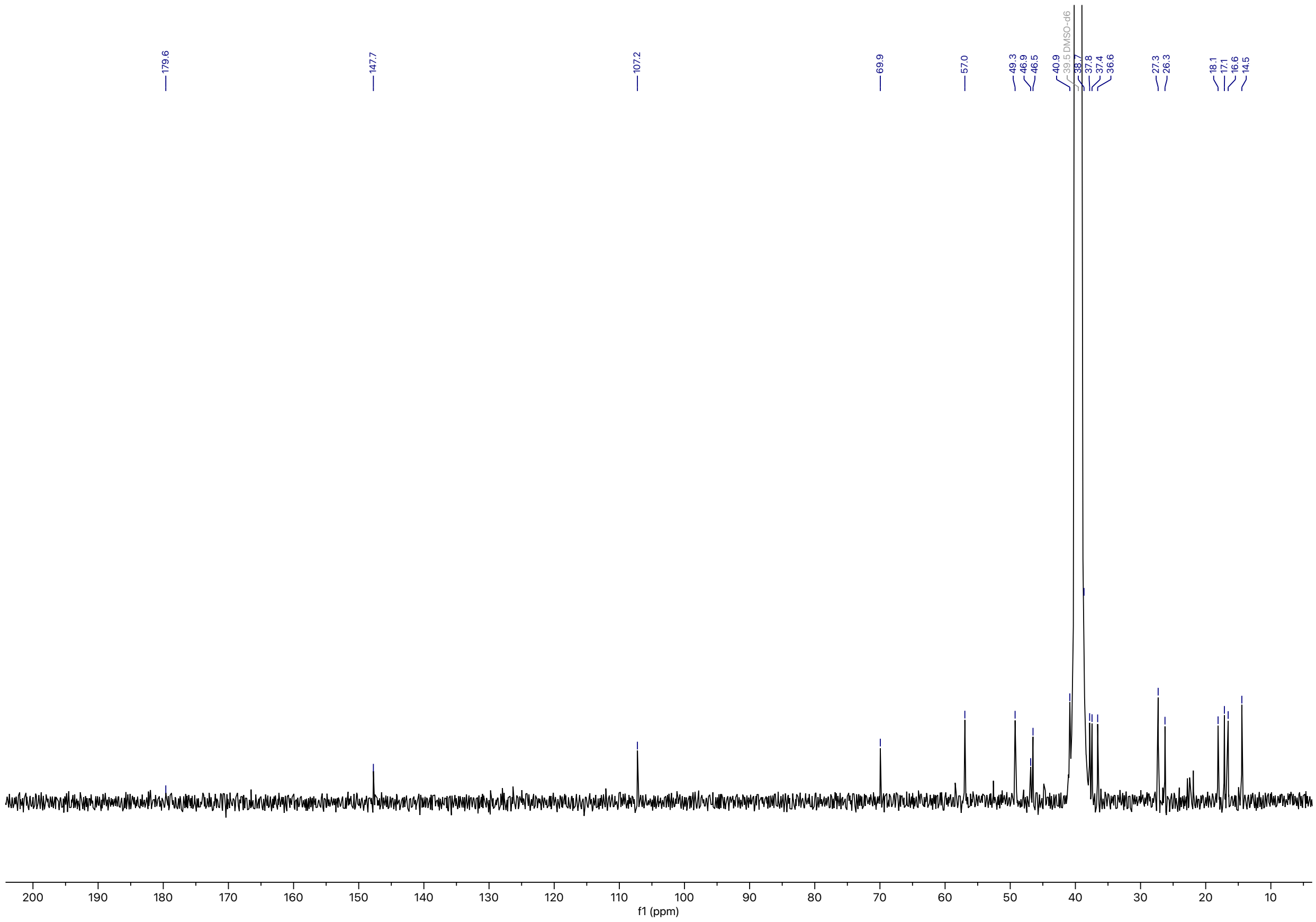
179.6
147.7
107.2
69.9
57.0
49.3
46.9
46.5
40.9
39.5 DMSO-d6
38.7
37.8
37.4
36.6
27.3
26.3
18.1
17.1
16.6
14.5
f1 (ppm)

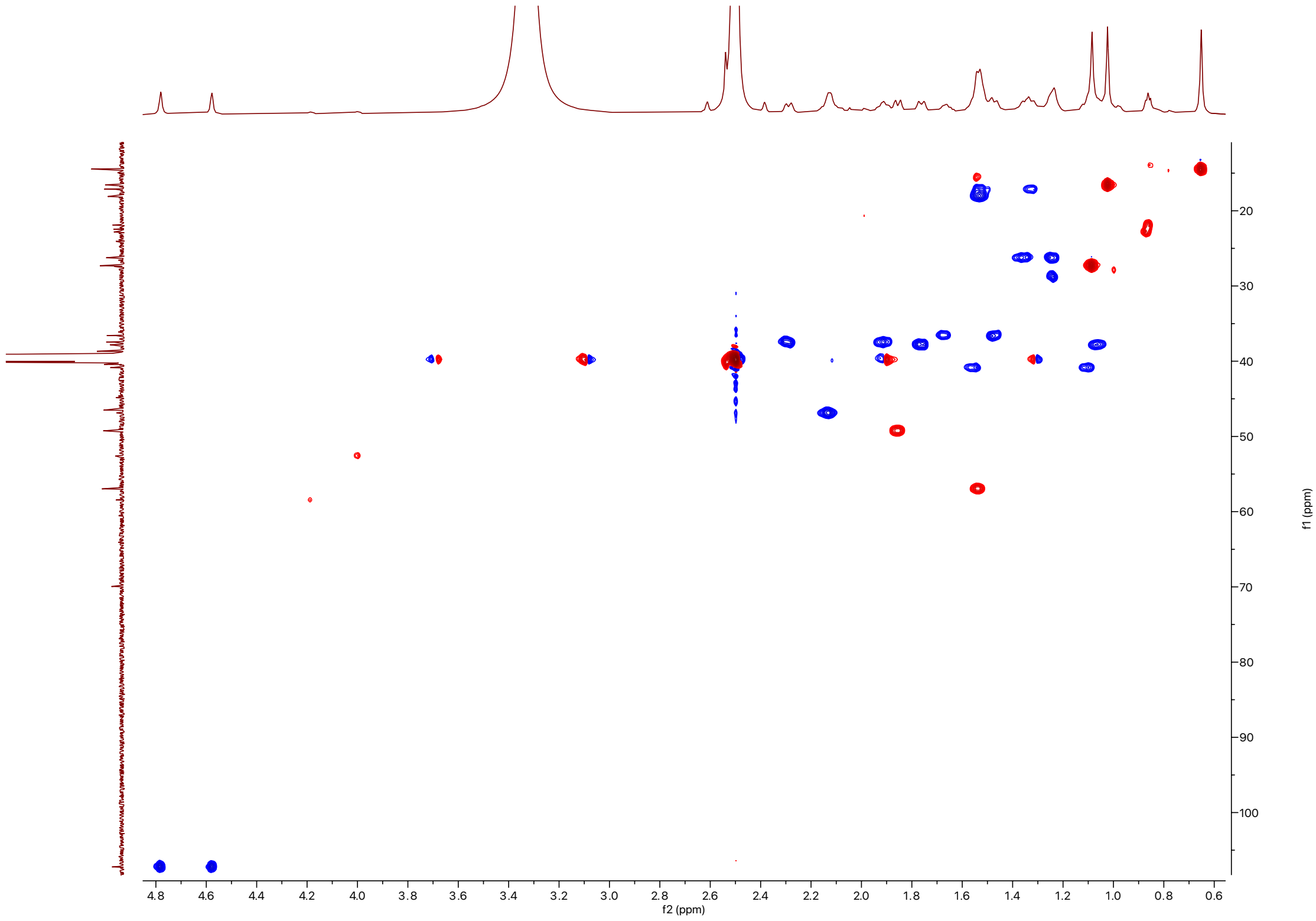
f2 (ppm)
f1 (ppm)

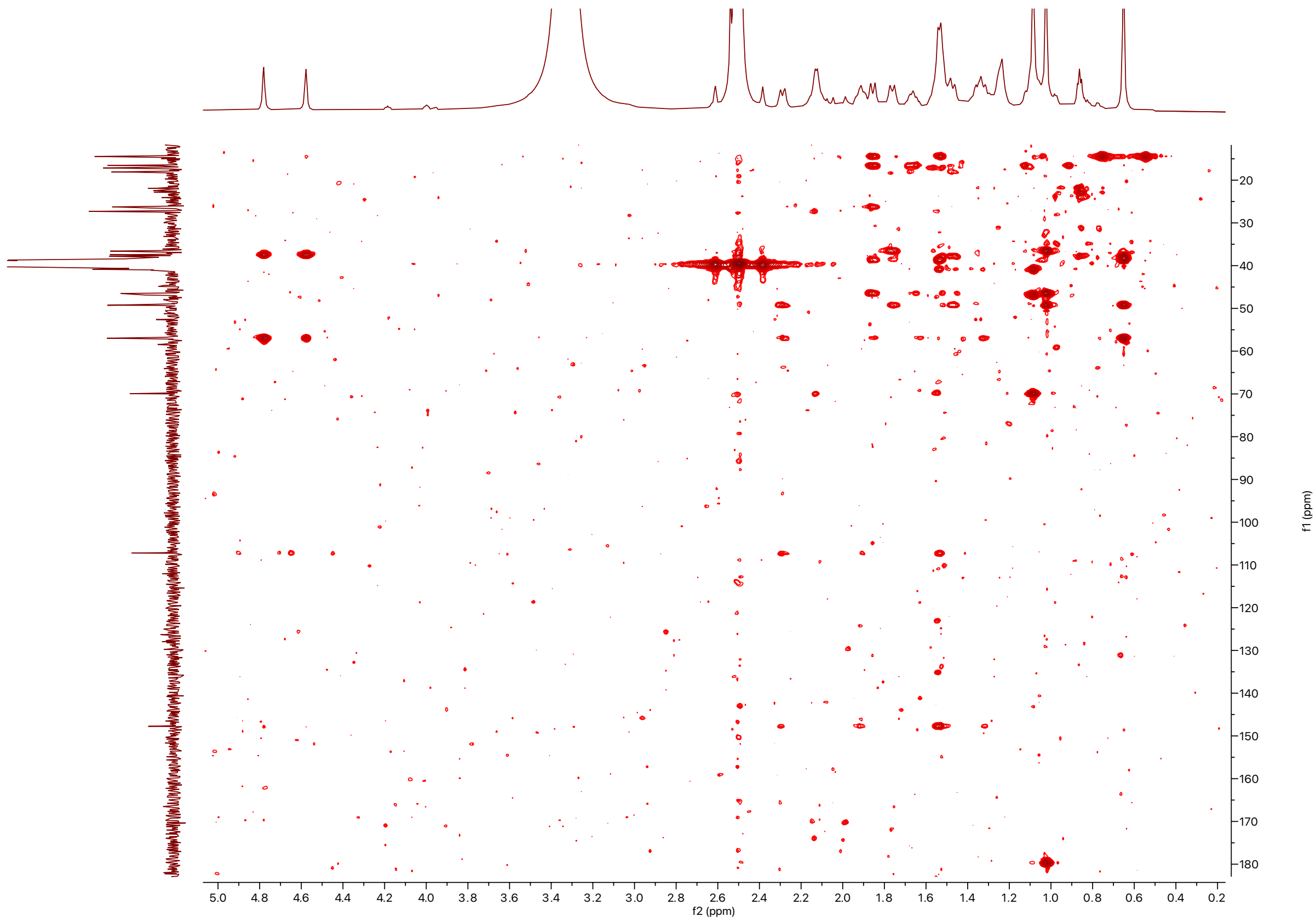
f2 (ppm)
f1 (ppm)

4.4.6 3α,13-Dihydroxy-*ent*-labd-8(17)-en-15-oic acid **18**

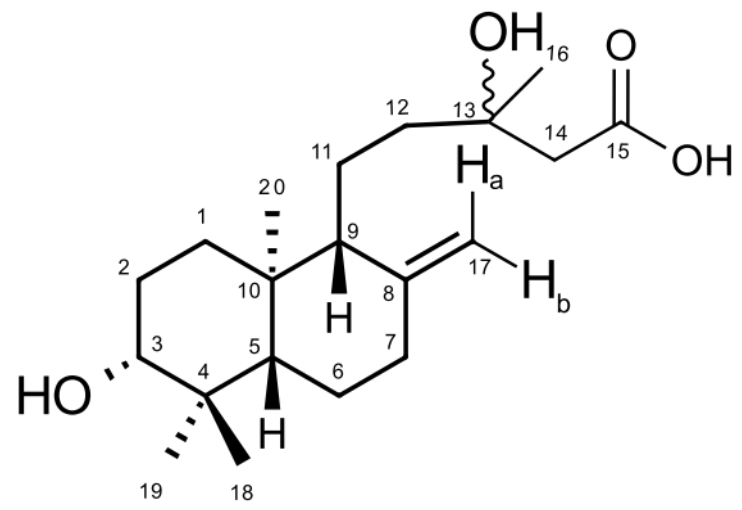

**Pos.**	**Measured in DMSO-d_6, 298K**				
	δ_C, 101 MHz	δ_H (*J*/Hz), 400 MHz	COSY	HMBC (H to C)	Selected NOESY correlations to H
**1**	37.2	a 1.10, overlapped	1b	-	1b
		b 1.73, ddd (13.2, 3.2, 3.2)	1a	-	1a
**2**	28.2	a 1.47, overlapped	3	-	-
		b 1.51, overlapped	3	-	-
**3**	77.2	3.03, dd (11.2, 4.7)	2a, 2b	4, 18, 19	18, 5, 2b, 1a
**4**	39.2	-	-	-	-
**5**	54.5	1.04, dd (12.6, 2.3)	6a, 6b	4, 6, 9, 18, 19, 20	9
**6**	24.1	a 1.29, overlapped	5, 7a, 7b	-	6b, 20
		b 1.68, overlapped	5, 7a, 7b	-	6a, 18
**7**	38.2	a 1.92, ddd (13.1, 12.7, 5.0)	6a, 6b, 7b, 17b	6, 8, 17	7b
		b 2.33, overlapped	7a, 6a, 6b	-	7a
**8**	148.4	-	-	-	-
**9**	57.1	1.46, overlapped	17a, 17b	-	5
**10**	39.6	-	-	-	-
**11**	17.8	a 1.32, overlapped	-	-	-
		b 1.54, overlapped	-	-	-
**12**	41.0	a 1.13, overlapped	-	-	12b
		b 1.63, overlapped	-	-	12a
**13**	70.7	-	-	-	-
**14**	46.6	a 2.28, d (14.2)	14b	12, 13, 15, 16	
		b 2.29, d, overlapped	14a	-	-
**15**	173.3	-	-	-	-
**16**	27.6	1.15, s	-	12, 13, 14	14b
**17**	107.3	a 4.57, brs	9, 17b	7, 8, 9	11a
		b 4.78, brs	7a, 9, 17a	7, 9	7b
**18**	28.8	0.90, s	19	3, 4, 5, 19	6a, 19
**19**	16.3	0.66, s	18	3, 4, 5, 18	18
**20**	14.8	0.62, s	-	1, 5, 9, 10	6a

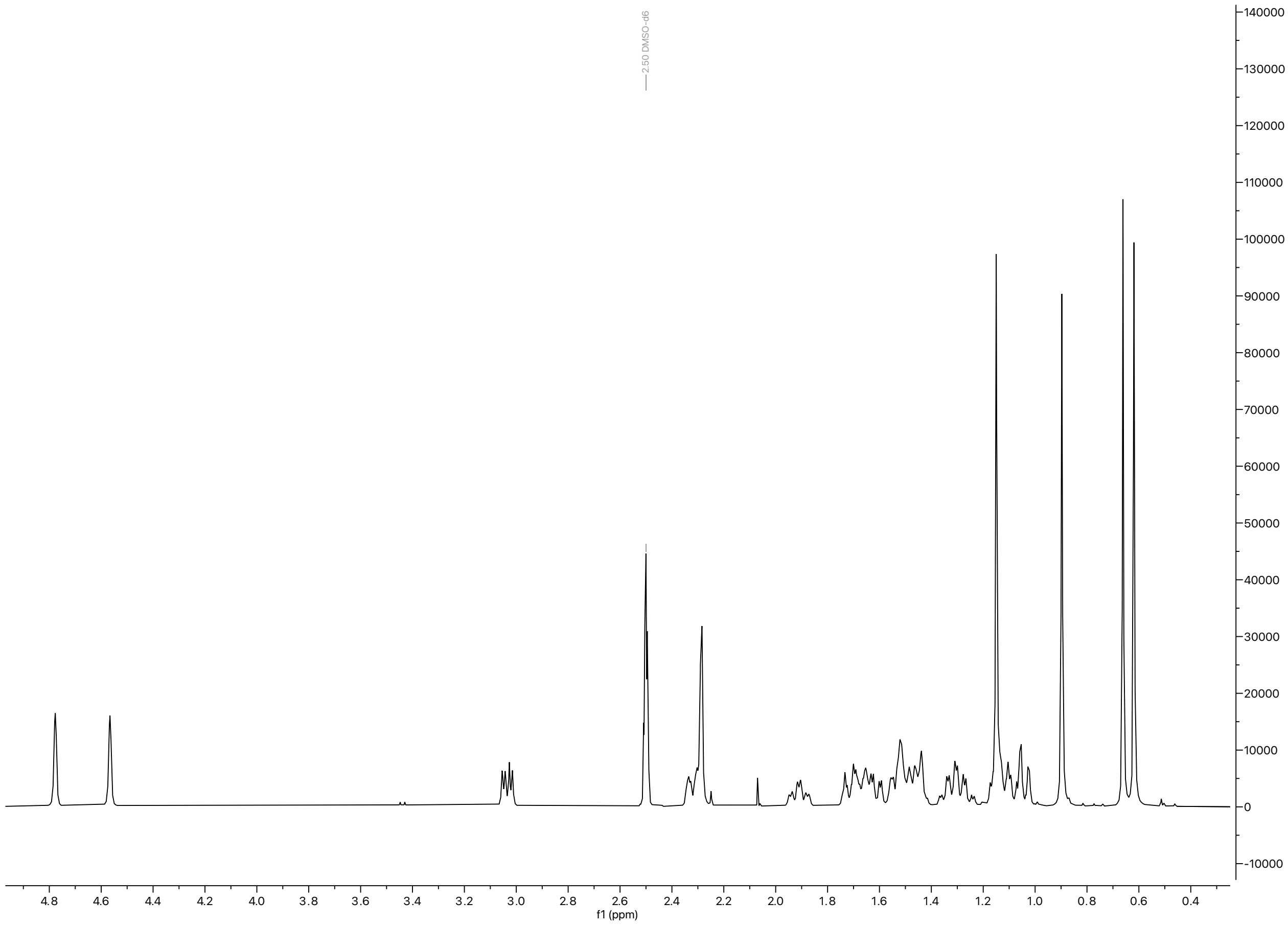
2.50 DMSO-d6
f1 (ppm)

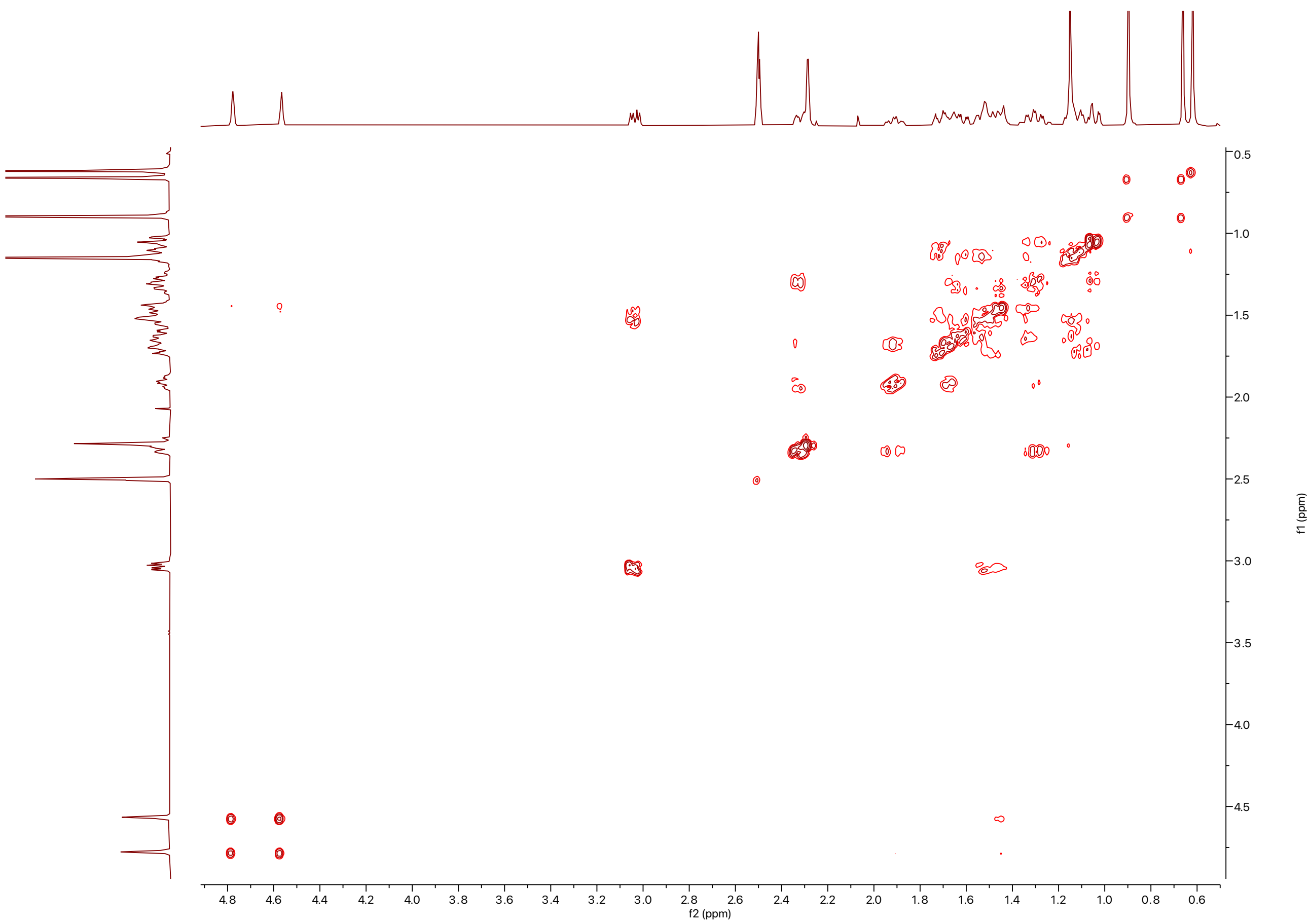
f2 (ppm)
f1 (ppm)

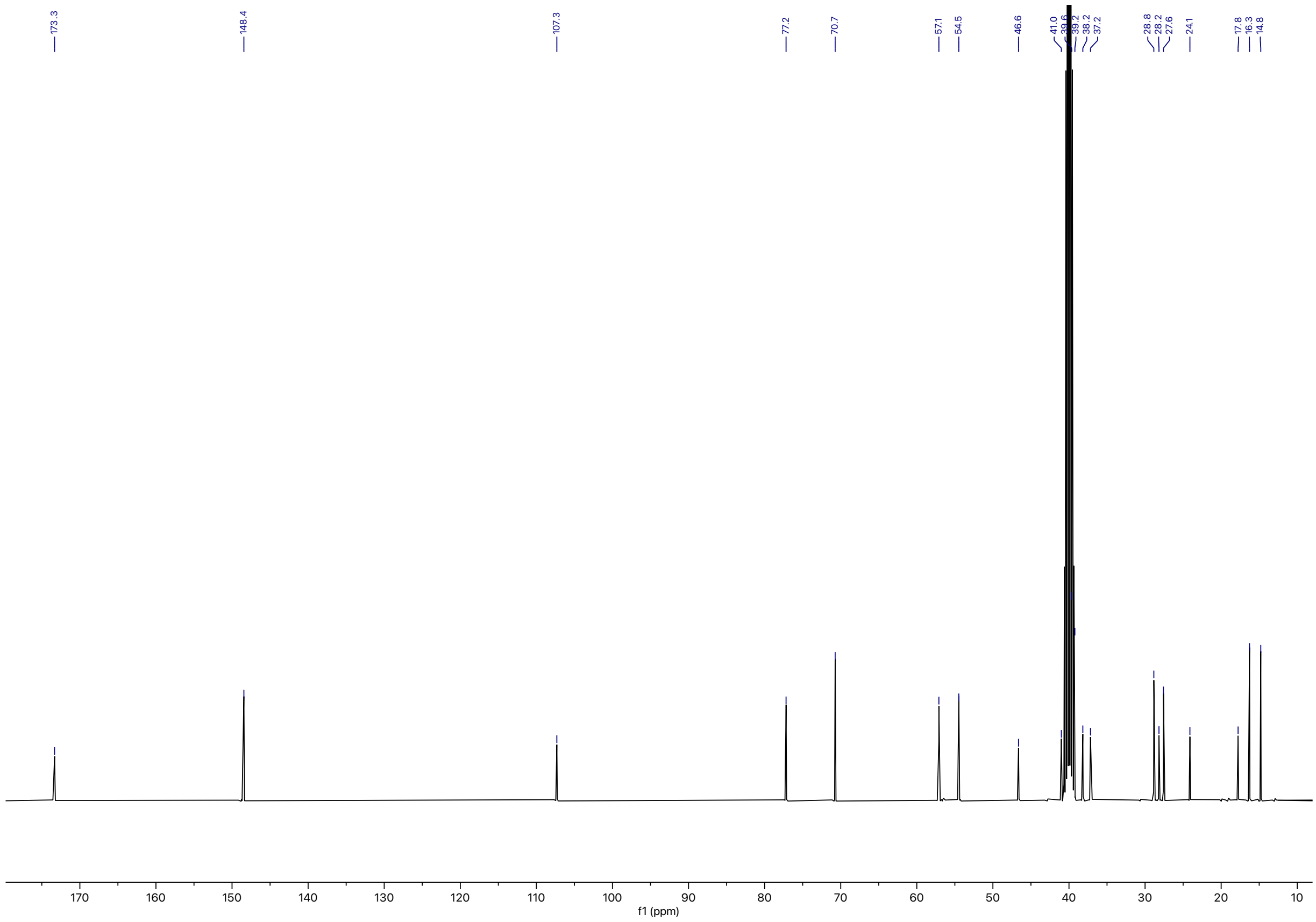
173.3
148.4
107.3
77.2
70.7
57.1
54.5
46.6
41.0
38.2
37.2
28.8
28.2
27.6
24.1
17.8
16.3
14.8
f1 (ppm)

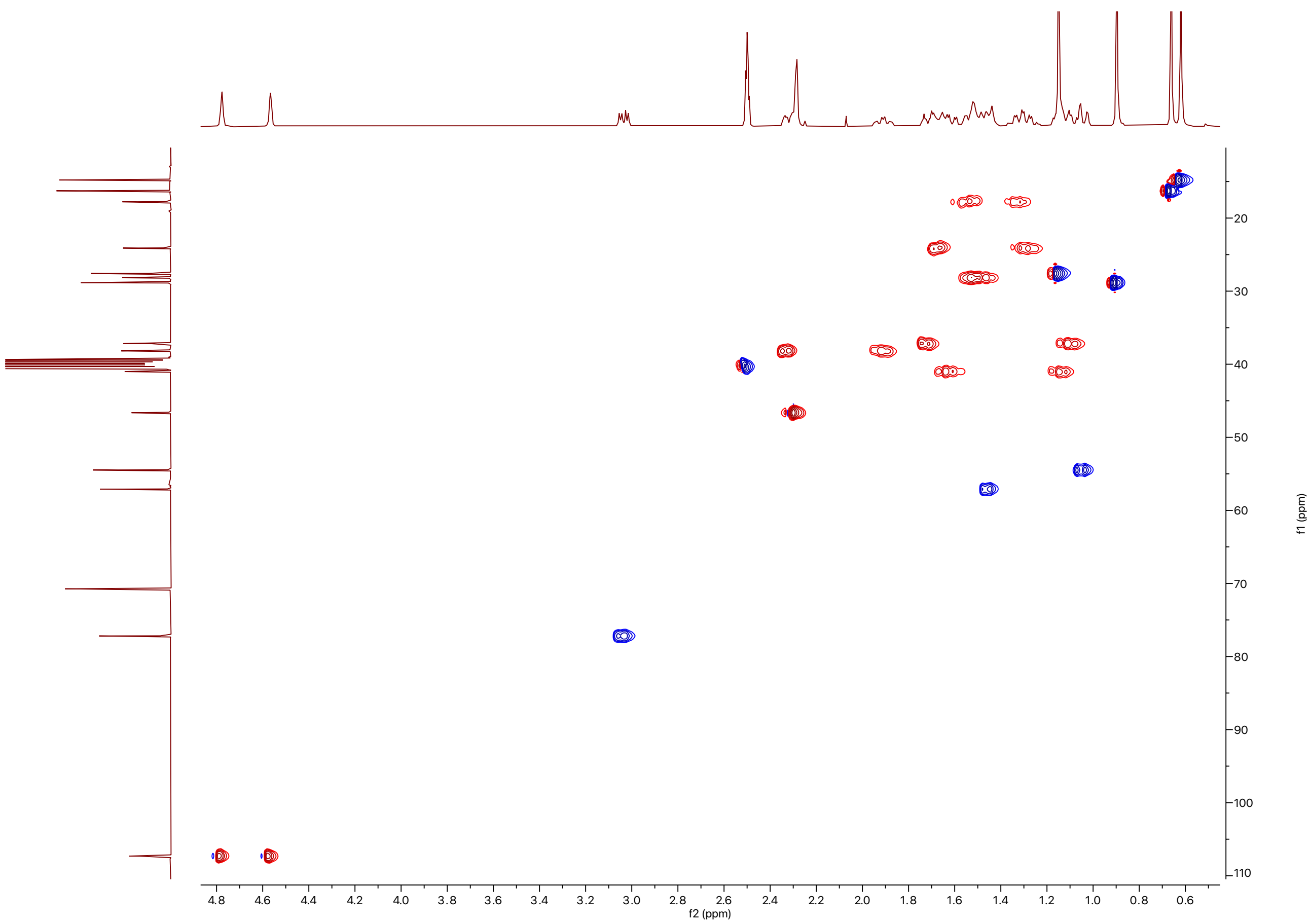
f2 (ppm)
f1 (ppm)

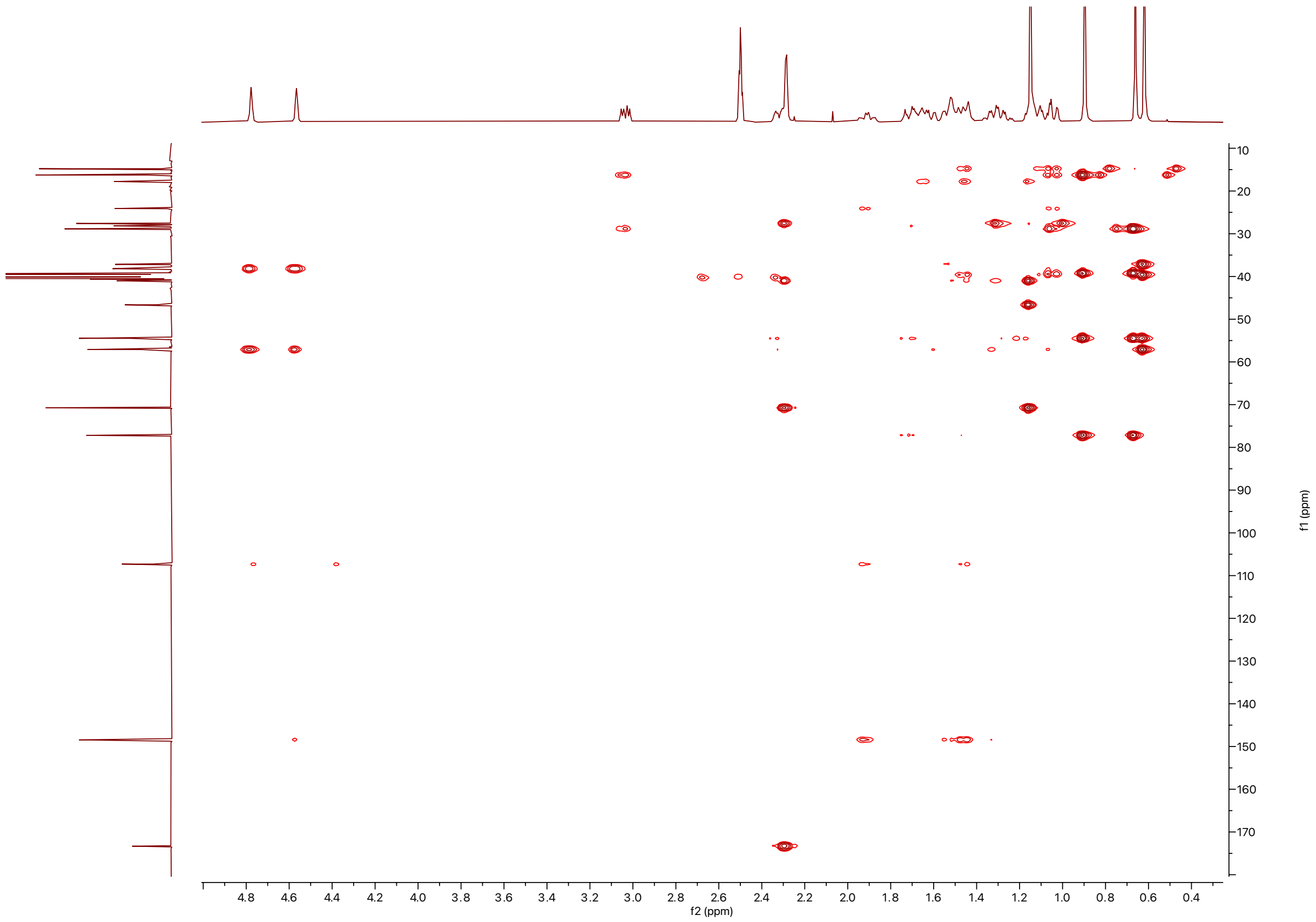
f2 (ppm)
f1 (ppm)

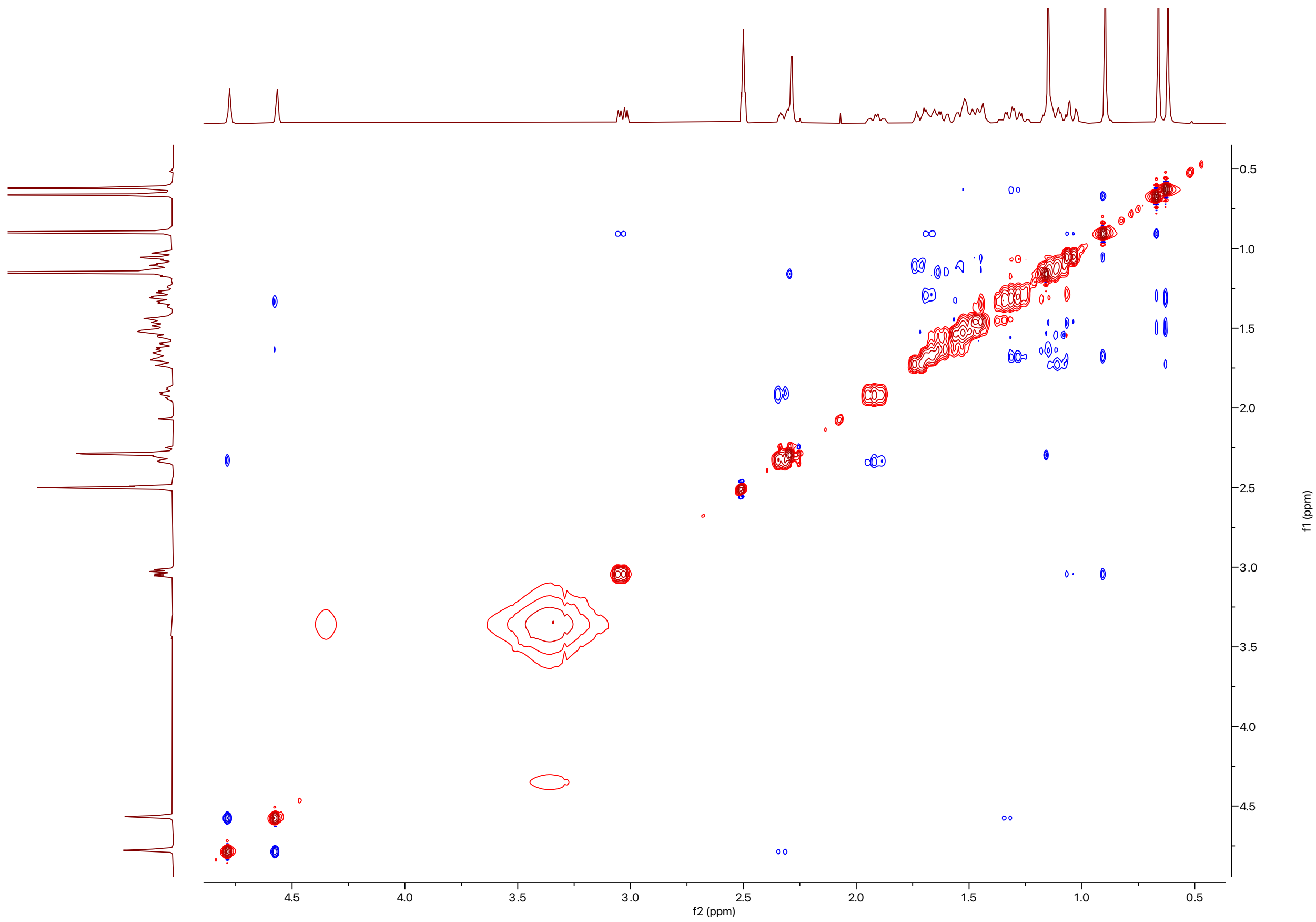
f2 (ppm)
f1 (ppm)

4.4.7 *ent*-13,18-Dihydroxy-*ent*-labd-8(17)-en-15-oic acid **19**

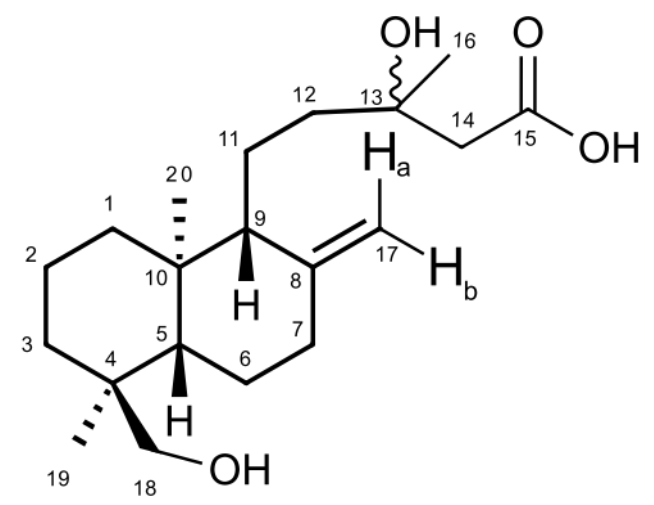

Pos.	Measured in MeOD, 298K*				
	δ_C, 126 MHz	δ_H (*J*/Hz), 600 MHz	COSY	HMBC (H to C)	Selected NOESY correlation to H
**1**	39.9	ax 1.07, ddd (13.1, 13.1, 3.5)	1eq, 2b	20	1eq, 2a
		eq 1.82, ddd (13.1, 3.2, 3.2)	1ax	3, 5	1ax, 20, 2a
**2**	19.9	a 1.55, m	2b	3	1eq
		b 1.65, overlapped	1ax, 2a	-	-
**3**	36.6	eq 1.24, ddd (13.2, 3.1, 3.1)	3ax	1, 2, 5	19
		ax 1.51, overlapped	3eq	-	-
**4**	39.0	-	-	-	-
**5**	49.4	1.50, overlapped	-	-	-
**6**	25.3	ax 1.32, m	7eq	5, 7	20
		eq 1.66, overlapped	7eq	-	-
**7**	39.2	ax 2.03, ddd (13.2, 12.7, 5.0)	6ax, 6eq, 7eq	6, 8, 17	6ax, 6eq, 7eq
		eq 2.36, ddd (12.7, 4.3, 2.4)	6ax, 6eq, 7ax	5, 8, 9, 17	7ax
**8**	149.8	-	-	-	-
**9**	58.9	1.62, brd (11.3)	11a, 17a	8, 10, 11, 12, 17, 20	1ax, 7ax
**10**	40.8	-	-	-	-
**11**	18.9	a 1.43, m	9, 11b	8, 12	20
		b 1.65, overlapped	11a	-	-
**12**	42.0	a 1.28, overlapped	12b	-	-
		b 1.79, ddd (13.4, 12.8, 4.4)	12a	11	-
**13**	72.6	-	-	-	-
**14**	46.4	a 2.43, d (14.6)	14b	12, 13, 15, 16	-
		b 2.44, d (14.6)	14a	12, 13, 15, 16	-
**15**	176.0	-	-	-	-
**16**	27.2	1.27, s	-	12, 13, 14	14a, 12b
**17**	107.2	a 4.59, dd (2.6, 1.6)	9, 17b	7, 9	11a, 12b, 17b
		b 4.82, dd (2.8, 1.6)	17a	7, 8, 9	7eq, 17a
**18**	72.0	a 2.99, d (11.1)	18b	3, 4, 5, 19	19, 5, 6eq, 3eq, 18b
		b 3.35, d (11.1)	18a	3, 4, 5, 19	19, 5, 6eq, 18a
**19**	18.1	0.73, s	-	3, 4, 5, 18	6ax, 3eq, 18a, 18b
**20**	15.5	0.76, s	-	1, 5, 9, 10	11b, 6ax

* note that ^{1}H and ^{13}C spectra were obtained on different instruments.

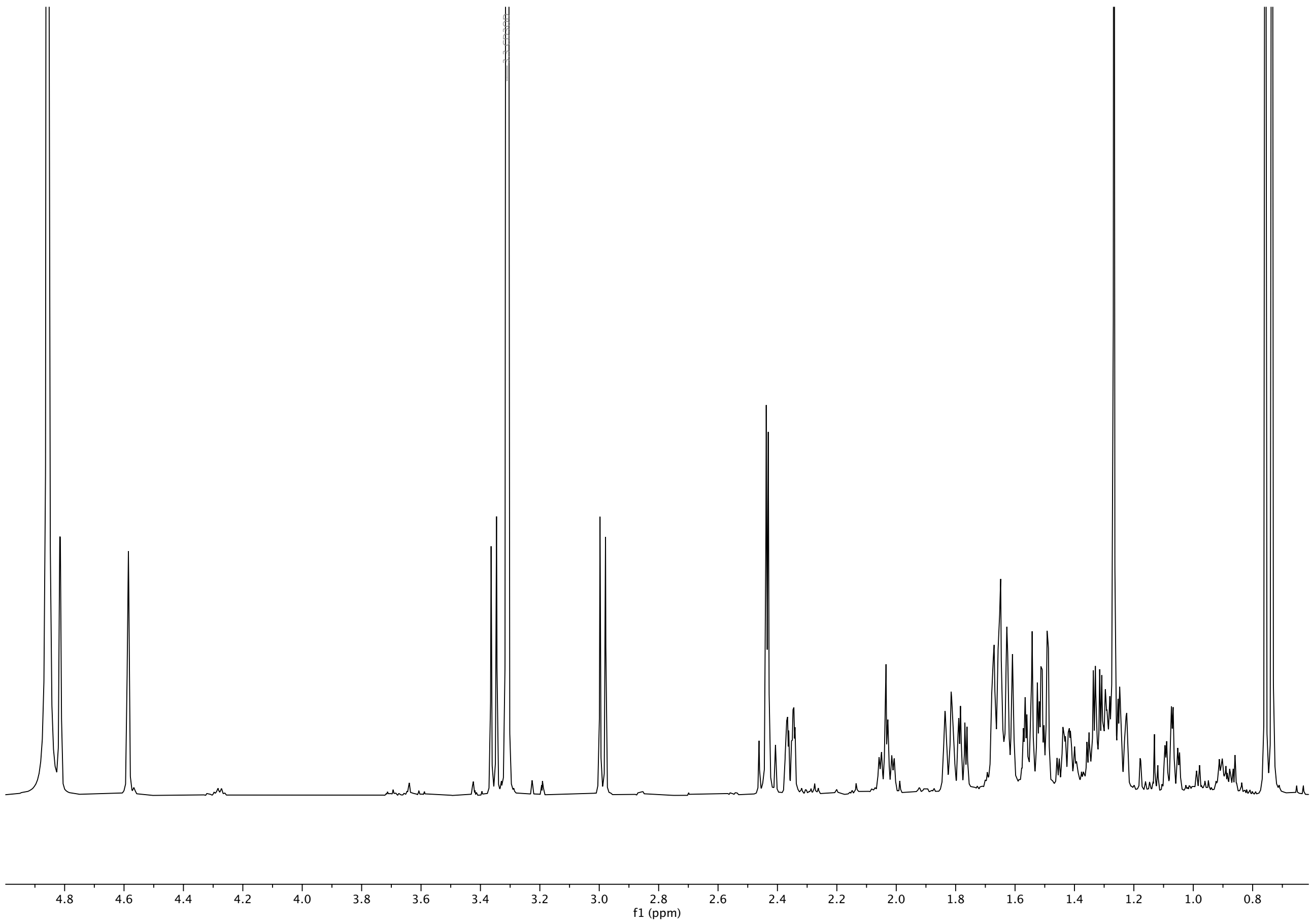
4.8 4.6 4.4 4.2 4.0 3.8 3.6 3.4 3.2 3.0 2.8 2.6 2.4 2.2 2.0 1.8 1.6 1.4 1.2 1.0 0.8
f1 (ppm)

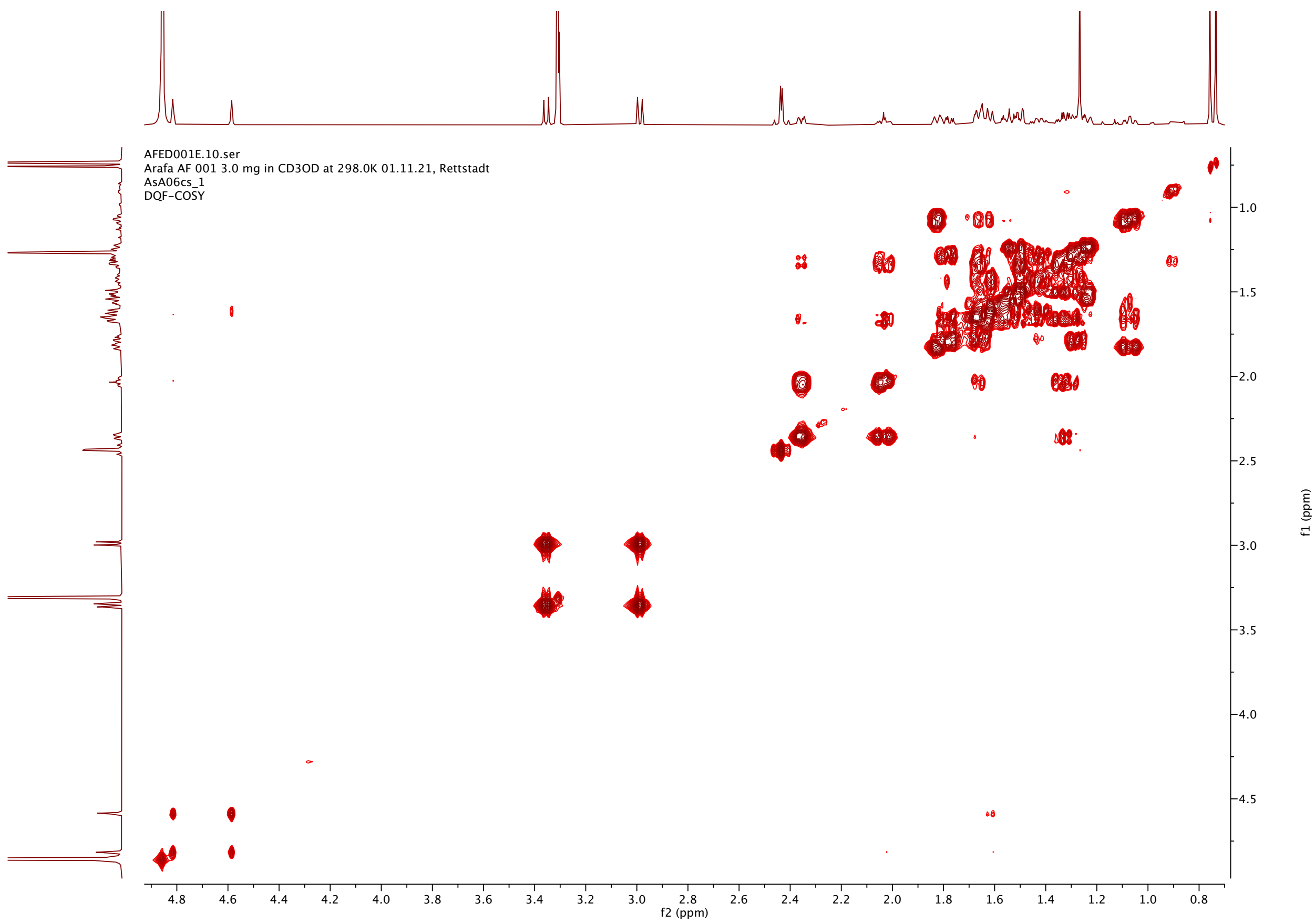
AFED001E.10.ser
Arafa AF 001 3.0 mg in CD3OD at 298.0K 01.11.21, Rettstadt
AsA06cs_1
DQF-COSY
4.8 4.6 4.4 4.2 4.0 3.8 3.6 3.4 3.2 3.0 2.8 2.6 2.4 2.2 2.0 1.8 1.6 1.4 1.2 1.0 0.8
f2 (ppm)
1.0 1.5 2.0 2.5 3.0 3.5 4.0 4.5
f1 (ppm)

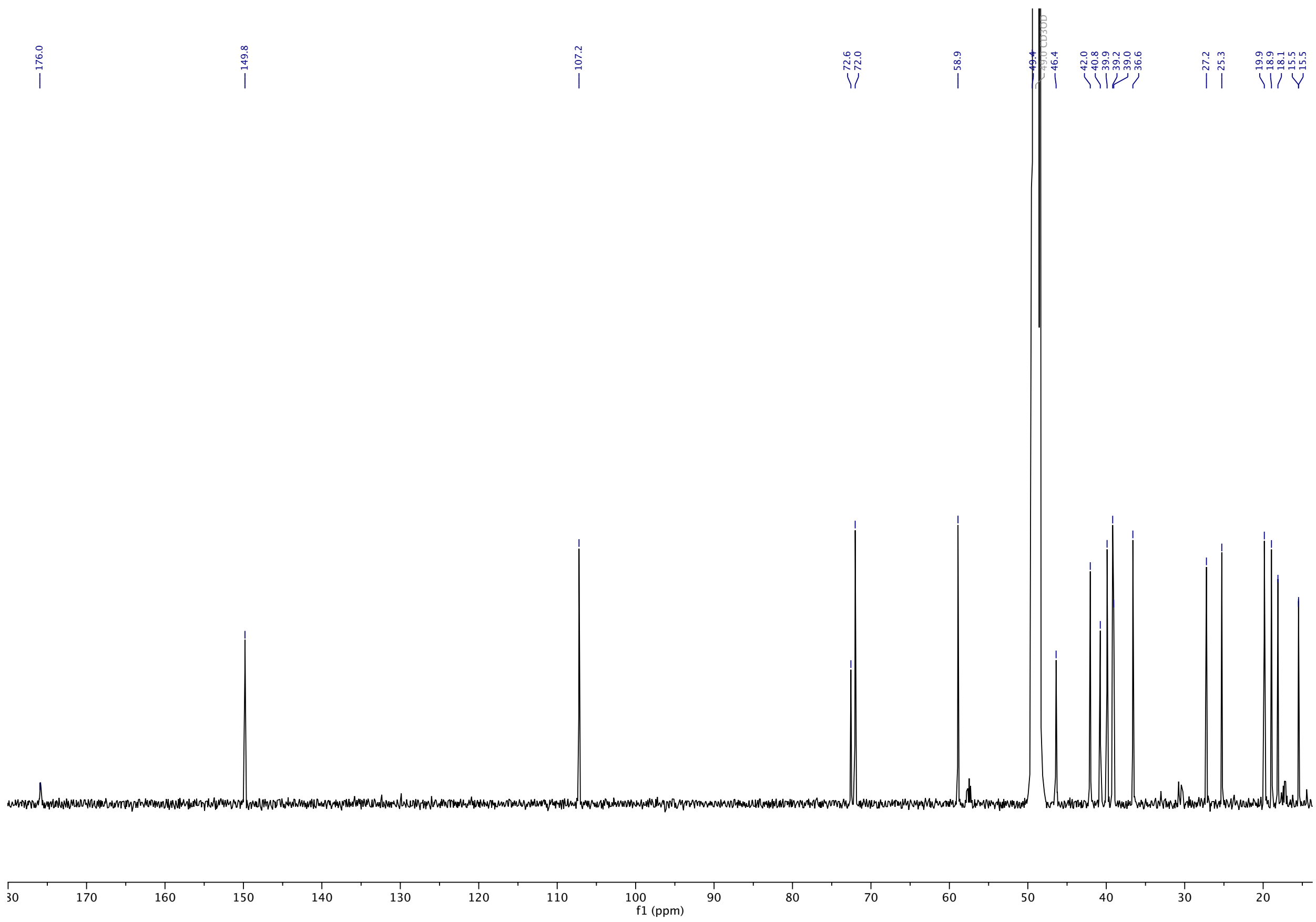

176.0
149.8
107.2
72.6
72.0
58.9
49.4
49.0 CD3OD
46.4
42.0
40.8
39.9
39.2
39.0
36.6
27.2
25.3
19.9
18.9
18.1
15.5
15.5
f1 (ppm)

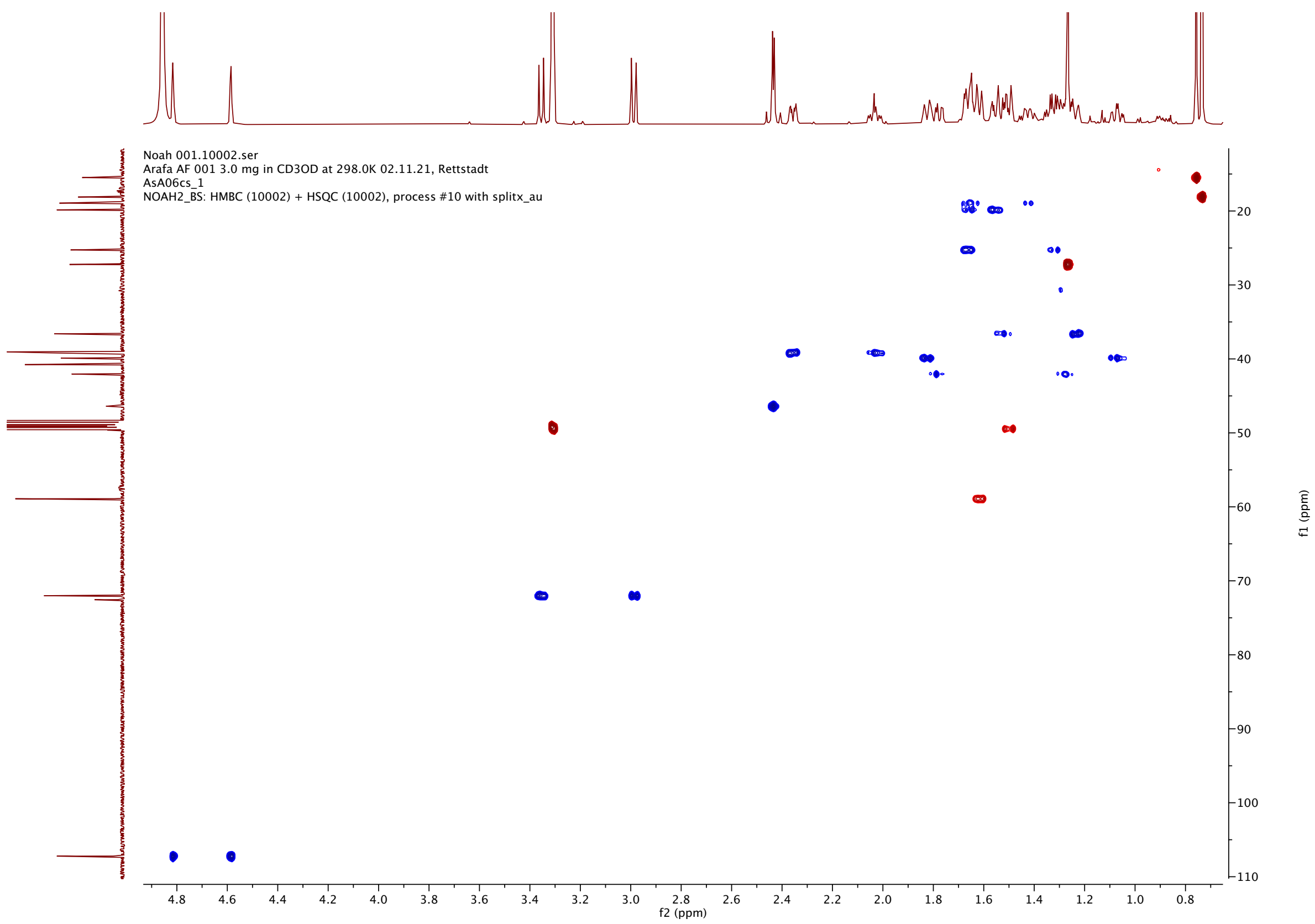

Noah 001.10002.ser
Arafa AF 001 3.0 mg in CD3OD at 298.0K 02.11.21, Rettstadt
AsA06cs_1
NOAH2_BS: HMBC (10002) + HSQC (10002), process #10 with splitx_au
f2 (ppm)
f1 (ppm)

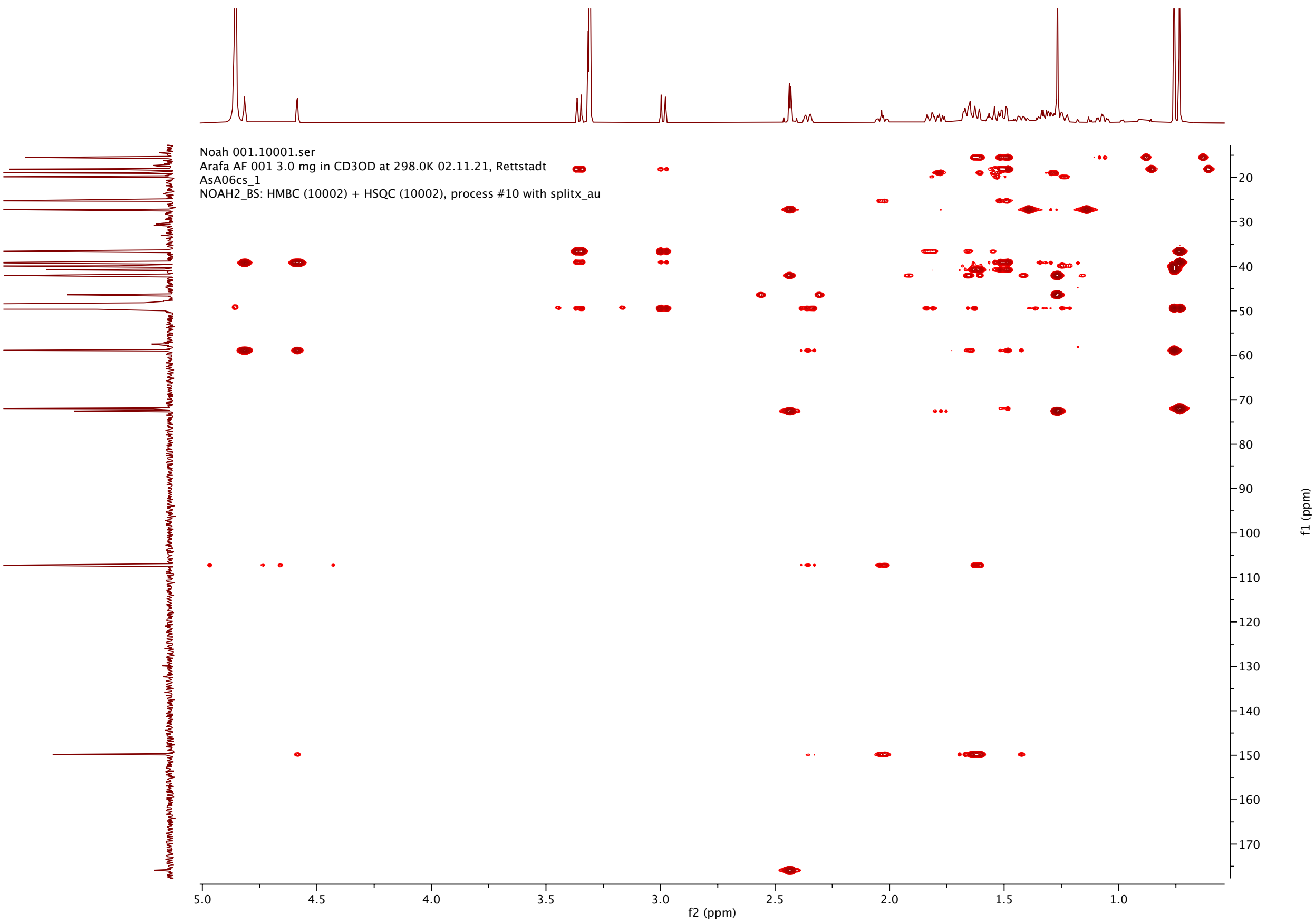
Noah 001.10001.ser
Arafa AF 001 3.0 mg in CD3OD at 298.0K 02.11.21, Rettstadt
AsA06cs_1
NOAH2_BS: HMBC (10002) + HSQC (10002), process #10 with splitx_au
f2 (ppm)
f1 (ppm)

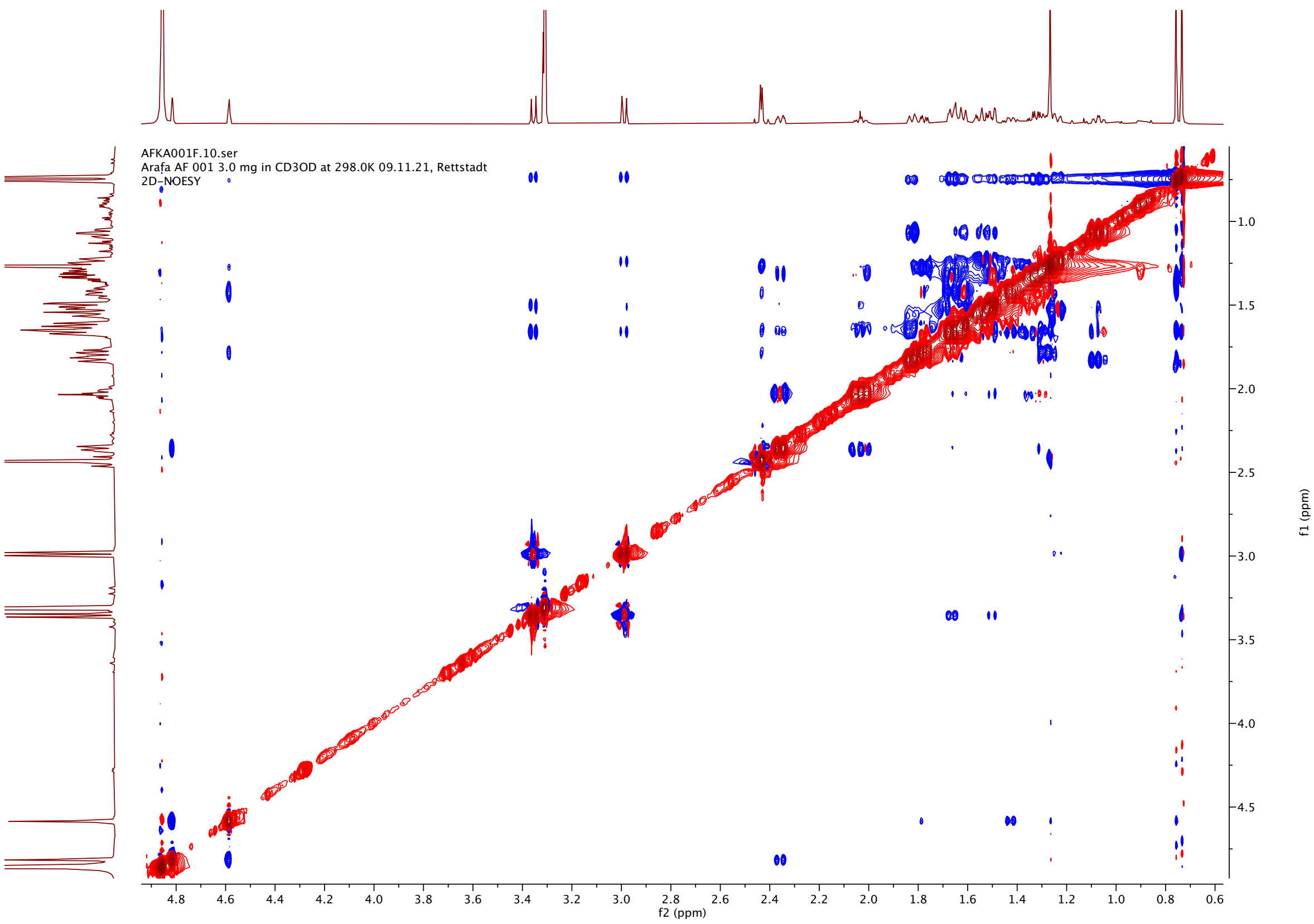
AFKA001F.10.ser
Arafa AF 001 3.0 mg in CD3OD at 298.0K 09.11.21, Rettstadt
2D-NOESY
f2 (ppm)
f1 (ppm)

4.4.8 *ent*-Labd-8(17),13-dien-15,18-dioic acid **20**

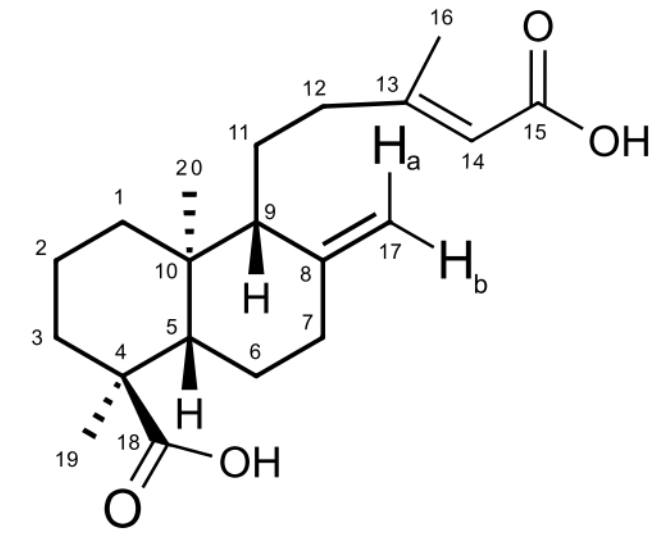

**Pos.**	**Measured in DMSO-d$_6$, 298K**			
	δ$_C$, 101 MHz	δ$_H$ (J/Hz), 400 MHz	COSY	HMBC (H to C)
**1**	37.6	a 1.05, overlapped	1b	-
		b 1.74, brd (12.7)	1a	3
**2**	18.1	1.53, overlapped	-	-
**3**	36.6	a 1.48, overlapped	-	-
		b 1.66, overlapped	-	-
**4**	46.5	-	-	-
**5**	49.2	1.86, dd (12.3, 2.9)	6a, 6b	4, 6, 10, 19, 20,
**6**	26.3	a 1.26, m	5	-
		b 1.36, m	5, 7b	-
**7**	37.4	a 1.92, overlapped	7b, 17a, 17b	-
		b 2.32, ddd (12.7, 4.3, 2.3)	6b, 7a	5
**8**	147.4	-	-	-
**9**	55.6	1.63, overlapped	17a, 17b	-
**10**	38.6	-	-	-
**11**	21.0	a 1.45, overlapped	12b	-
		b 1.60, overlapped	12b	-
**12**	39.0	a 1.94, overlapped	12b,	-
		b 2.19, ddd (14.0, 10.2, 4.5)	11a, 11b, 12a	16
**13**	159.0	-	-	-
**14**	116.1	5.54, brs	16	12, 13, 14, 15
**15**	167.5	-	-	-
**16**	18.3	2.07, d (1.3)	14	12, 13, 14
**17**	107.0	a 4.52, brs	7a, 9, 17b	7, 8, 9
		b 4.85, brs	7a, 9, 17a	7, 8, 9
**18**	179.6	-	-	-
**19**	16.5	1.02, s	-	3, 4, 5, 18
**20**	14.5	0.66, s	-	1, 5, 9, 10

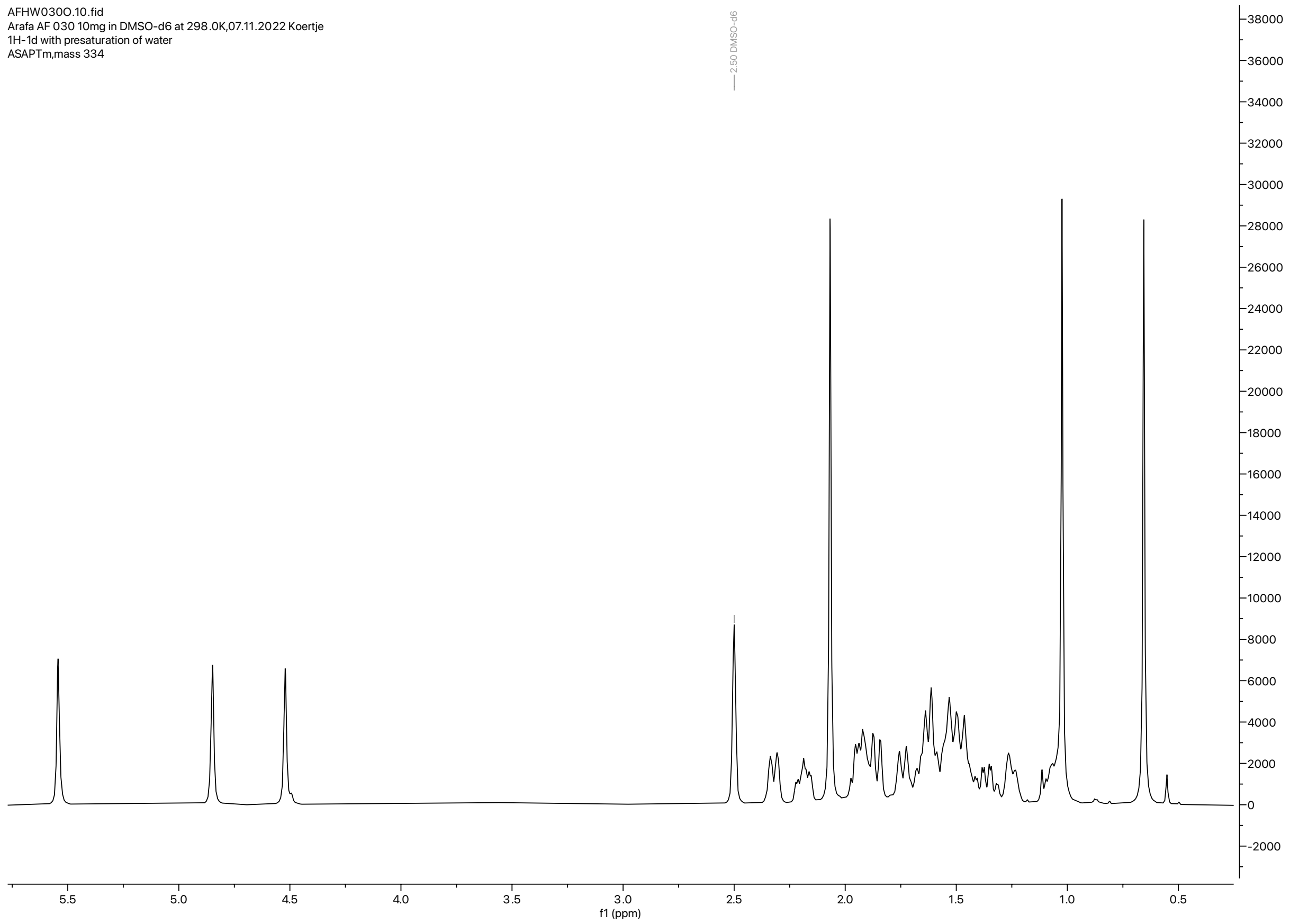
AFHW030O.10.fid
Arafa AF 030 10mg in DMSO-d6 at 298.0K,07.11.2022 Koertje
1H-1d with presaturation of water
ASAPTm,mass 334
2.50 DMSO-d6
f1 (ppm)

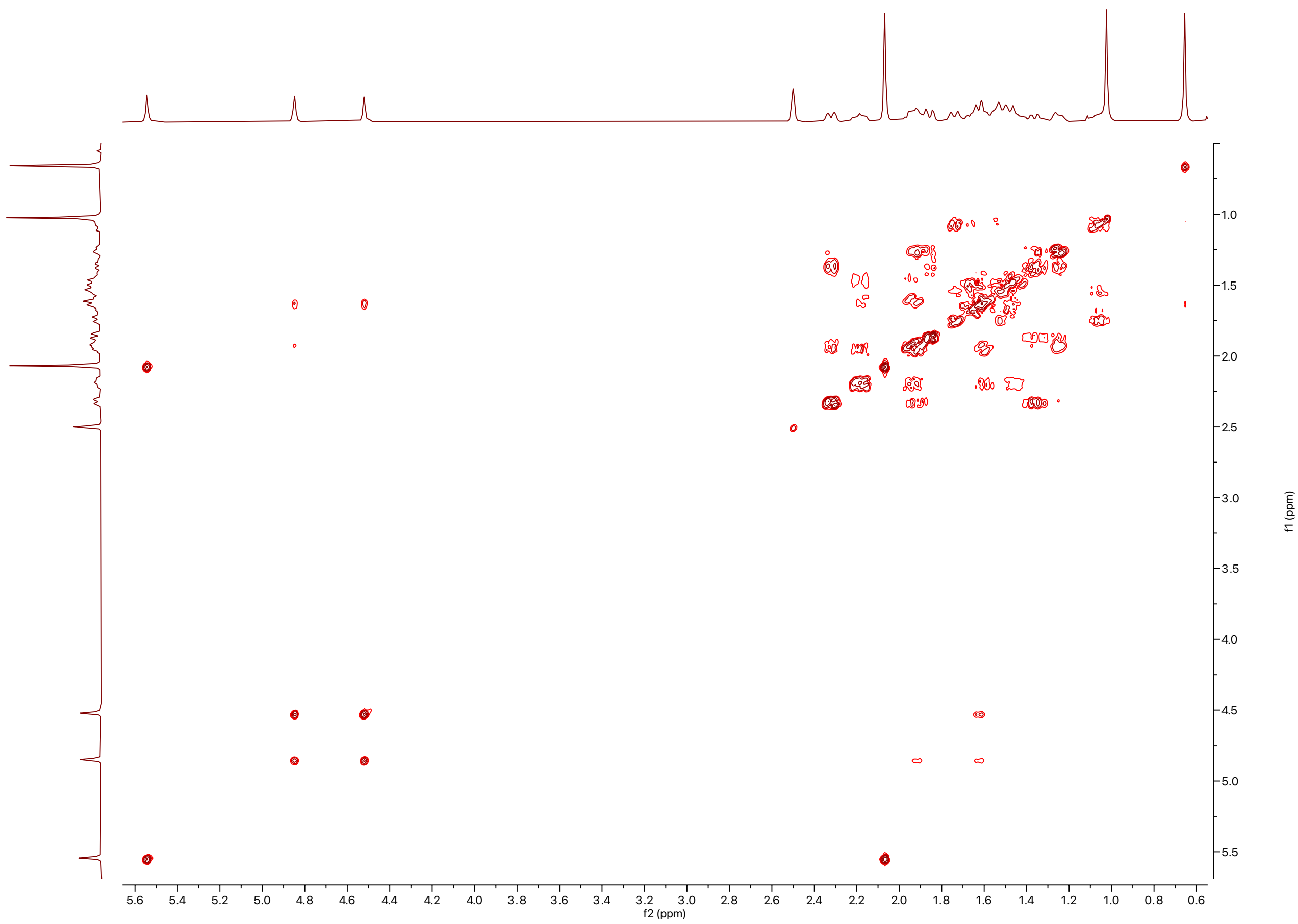
f2 (ppm)
f1 (ppm)

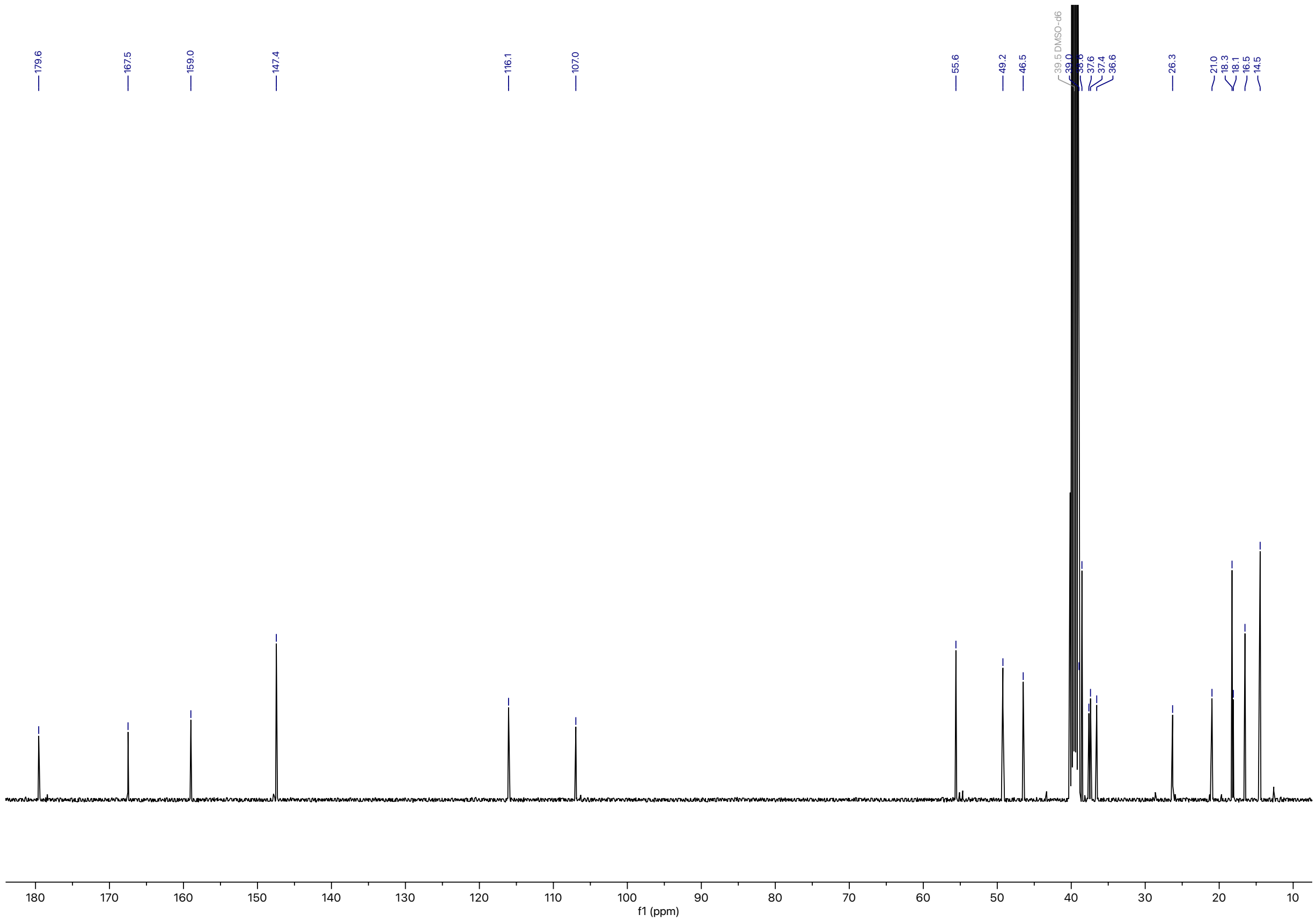
179.6
167.5
159.0
147.4
116.1
107.0
55.6
49.2
46.5
39.5 DMSO-d6
39.0
38.6
37.6
37.4
36.6
26.3
21.0
18.3
18.1
16.5
14.5
f1 (ppm)

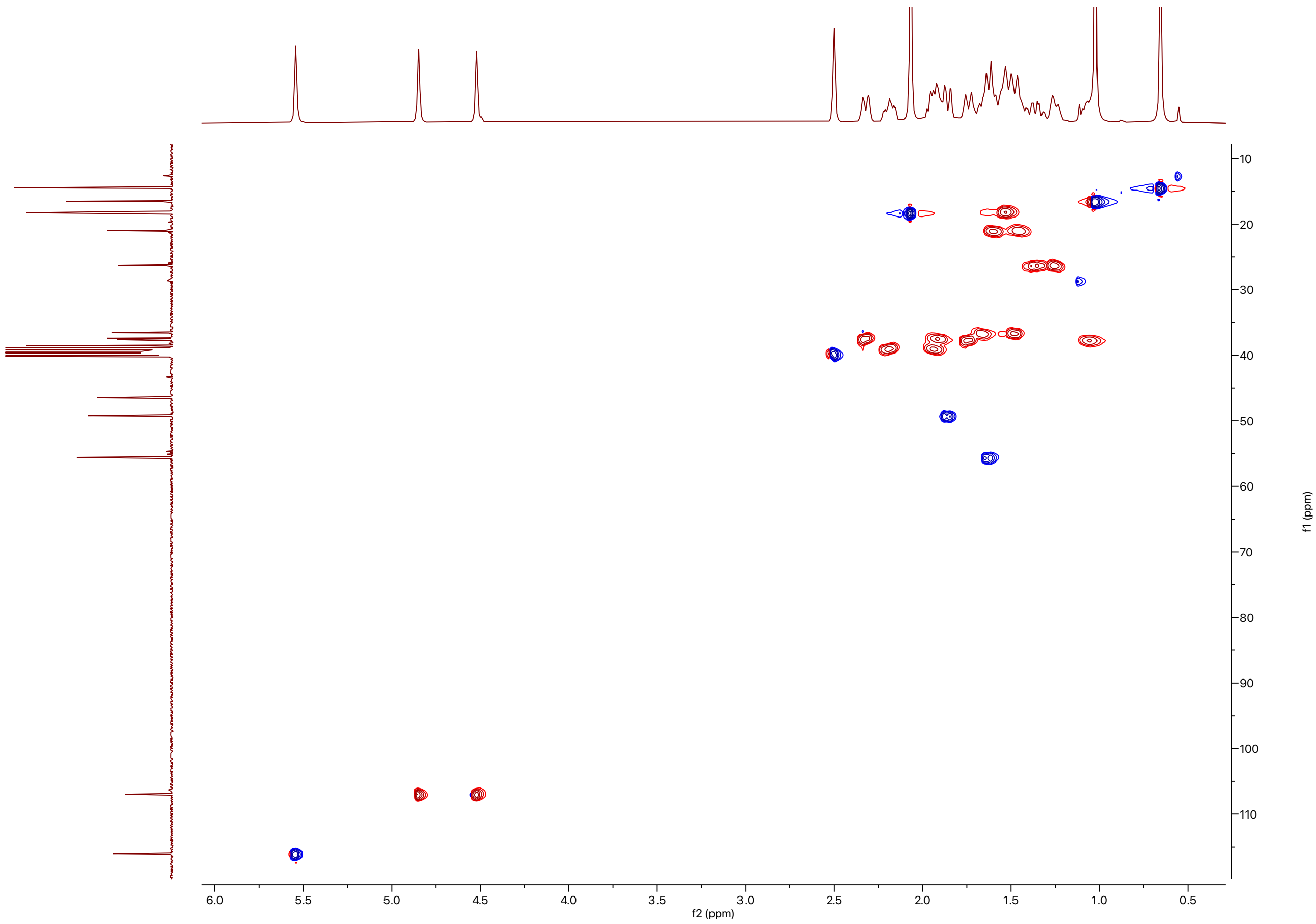
f2 (ppm)
f1 (ppm)

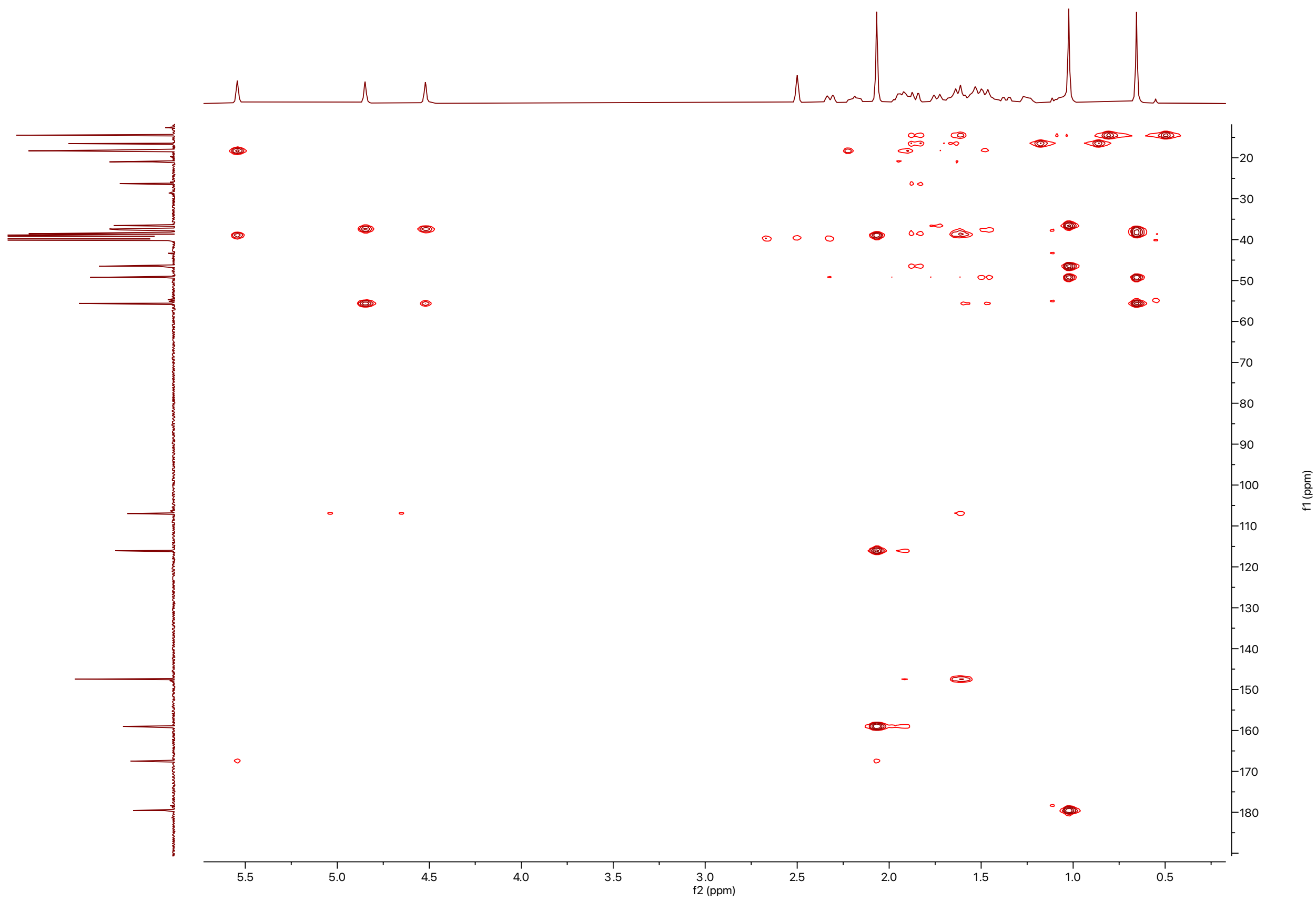

f1 (ppm)
f2 (ppm)

4.4.9 *ent*-Labd-8(17)-en-15,18-dioic acid **21**

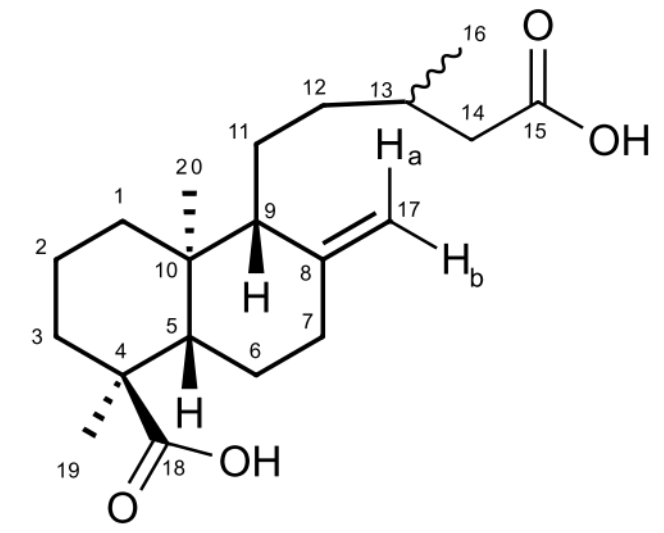

**Pos.**	Measured in DMSO-d_6, 298K				
	δ_C, 101 MHz	δ_H (*J*/Hz), 400 MHz	COSY	HMBC (H to C)	Selected NOESY Correlations to H
**1**	37.7	ax 1.05, overlapped	H-2, H-1eq	-	H-1eq
		eq 1.75, brd (13.3)	H-1ax, H-2	3	H-1ax, CH_3-20
**2**	18.1	ax 1.52, overlapped	-	-	
		eq 1.52, overlapped	-	-	
**3**	36.6	a 1.47, overlapped	H-3b	-	
		b 1.66, m	H-3a	2, 18, 19	
**4**	46.5	-	-	-	-
**5**	49.2	1.86, dd (12.3, 2.9)	H-6a, H-6b	4, 6, 10, 18, 19, 20	H-9
**6**	26.3	a 1.24, overlapped	-	-	
		b 1.35, overlapped	-	-	
**7**	37.4	ax 1.91, overlapped	-	-	H-7eq
		eq 2.30, ddd (12.7, 4.3, 2.3)	H-6a, H-6b, H-7ax	5, 6, 8, 9, 17,	H-7ax, H-17b
**8**	147.8	-	-	-	-
**9**	56.7	1.57, brd (10.8)	H-11b, H-17a, H17b	7, 11, 17, 18, 20	H-5
**10**	38.6	-	-	-	-
**11**	20.3	a 1.25, overlapped	-	-	
		b 1.45, overlapped	-	-	
**12**	35.3	a 0.93, m	11b	-	
		b 1.41, overlapped	9	-	
**13**	30.4	1.79, overlapped	-	-	CH_3-16
**14**	41.2	a 1.98, dd (14.9, 7.9)	12a, 13, 14b	12, 13, 15, 16	H-14b, CH_3-16
		b 2.19, dd (14.9, 6.0)	13, 14a	12, 13, 15, 16	H-14a, H-13
**15**	174.0	-	-	-	-
**16**	19.9	0.88, d (6.6)	13	12, 13, 14	H-14a, H-13
**17**	106.9	a 4.49, brs	H-9, H-17b	7, 8, 9	H-17b
		b 4.80, brs	H-7ax, H-9, H-17a	7, 9	H-7b
**18**	179.6	-	-	-	-
**19**	16.5	1.02, s	-	3, 4, 5, 18	H-20, H-2, H-6b
**20**	14.4	0.64, s	-	1, 5, 9, 10	H-1eq, H-2ax, H-6b, CH_3-19

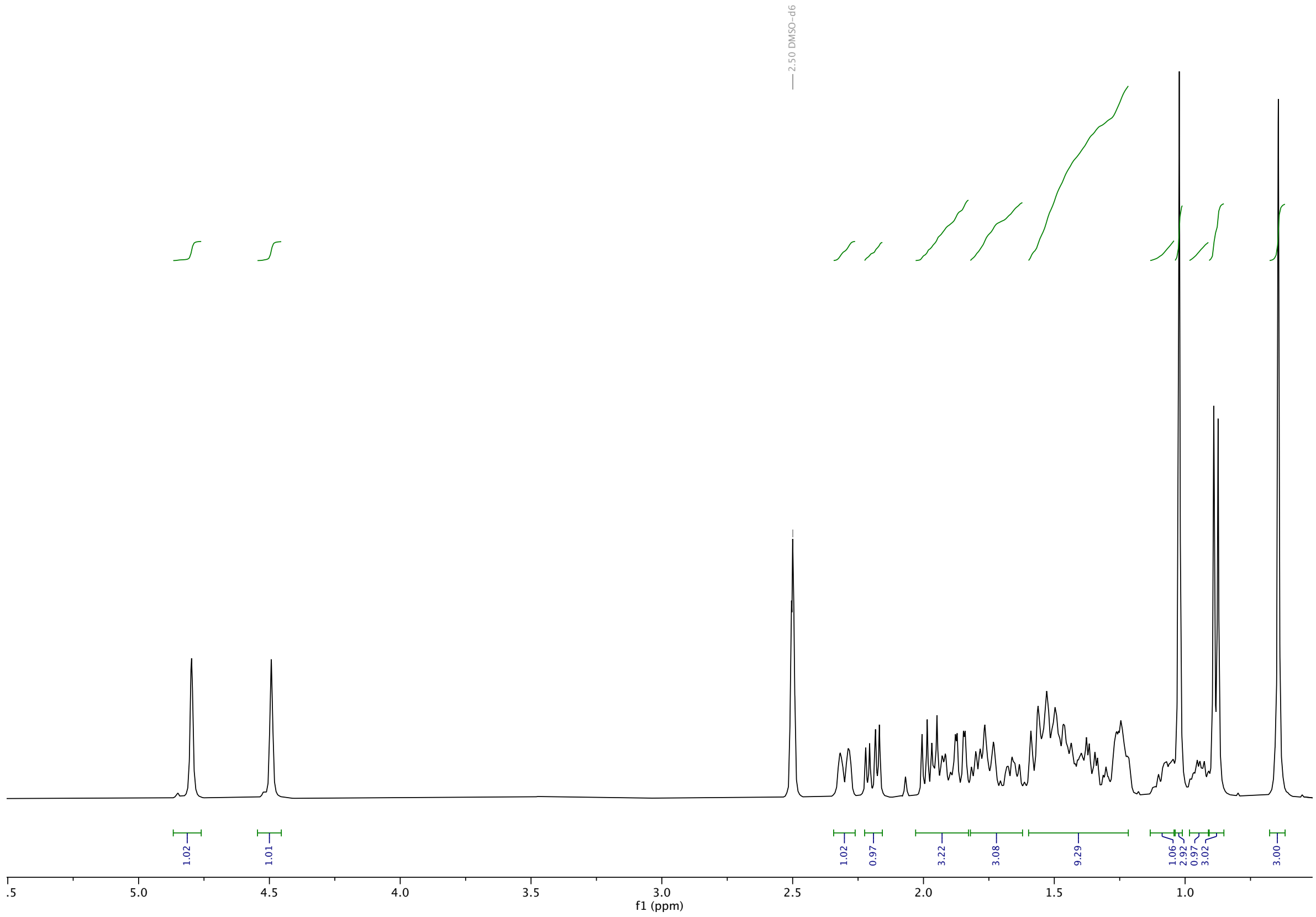

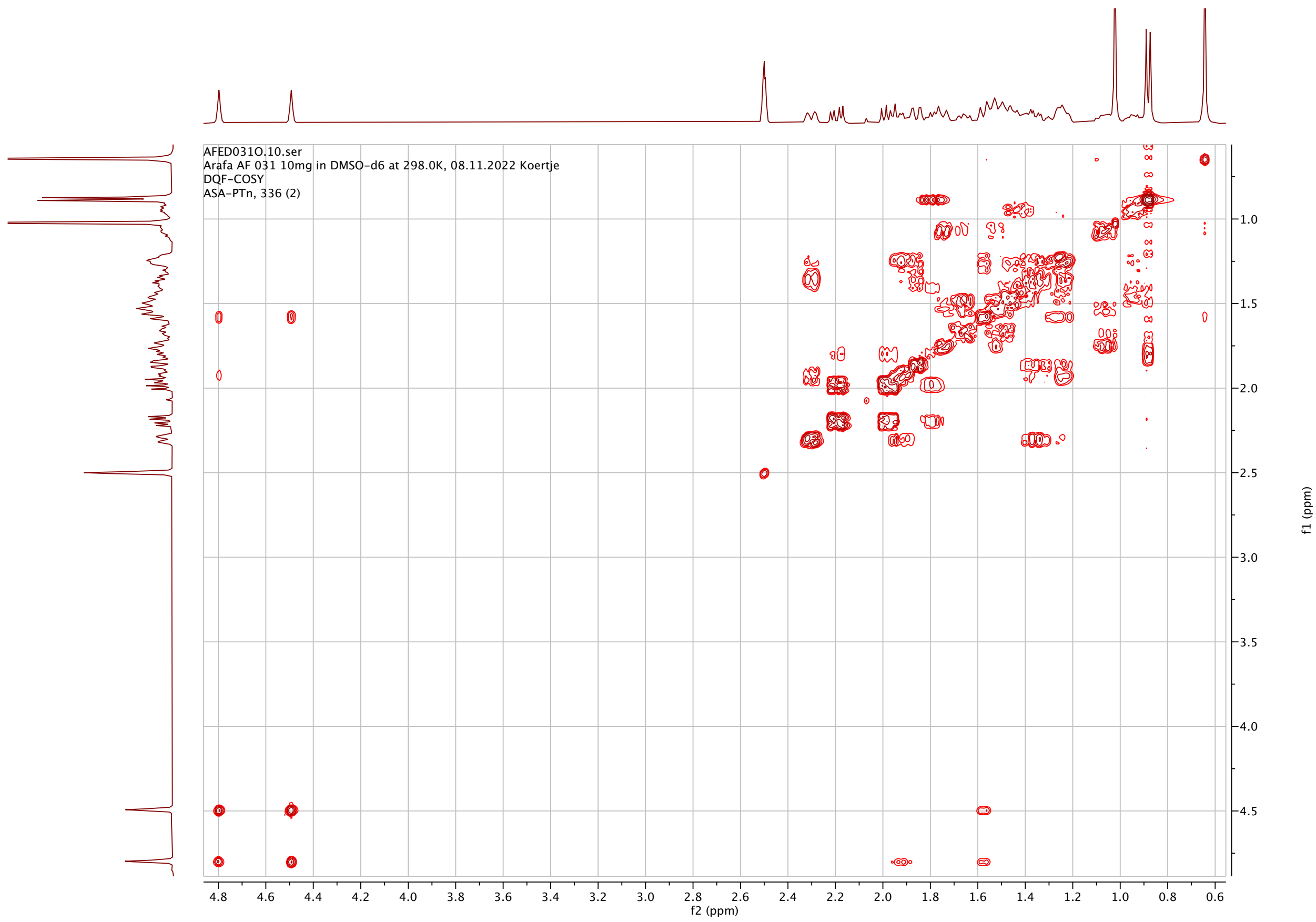
AFED031O.10.ser
Arafa AF 031 10mg in DMSO-d6 at 298.0K, 08.11.2022 Koertje
DQF-COSY
ASA-PTn, 336 (2)
f2 (ppm)
f1 (ppm)

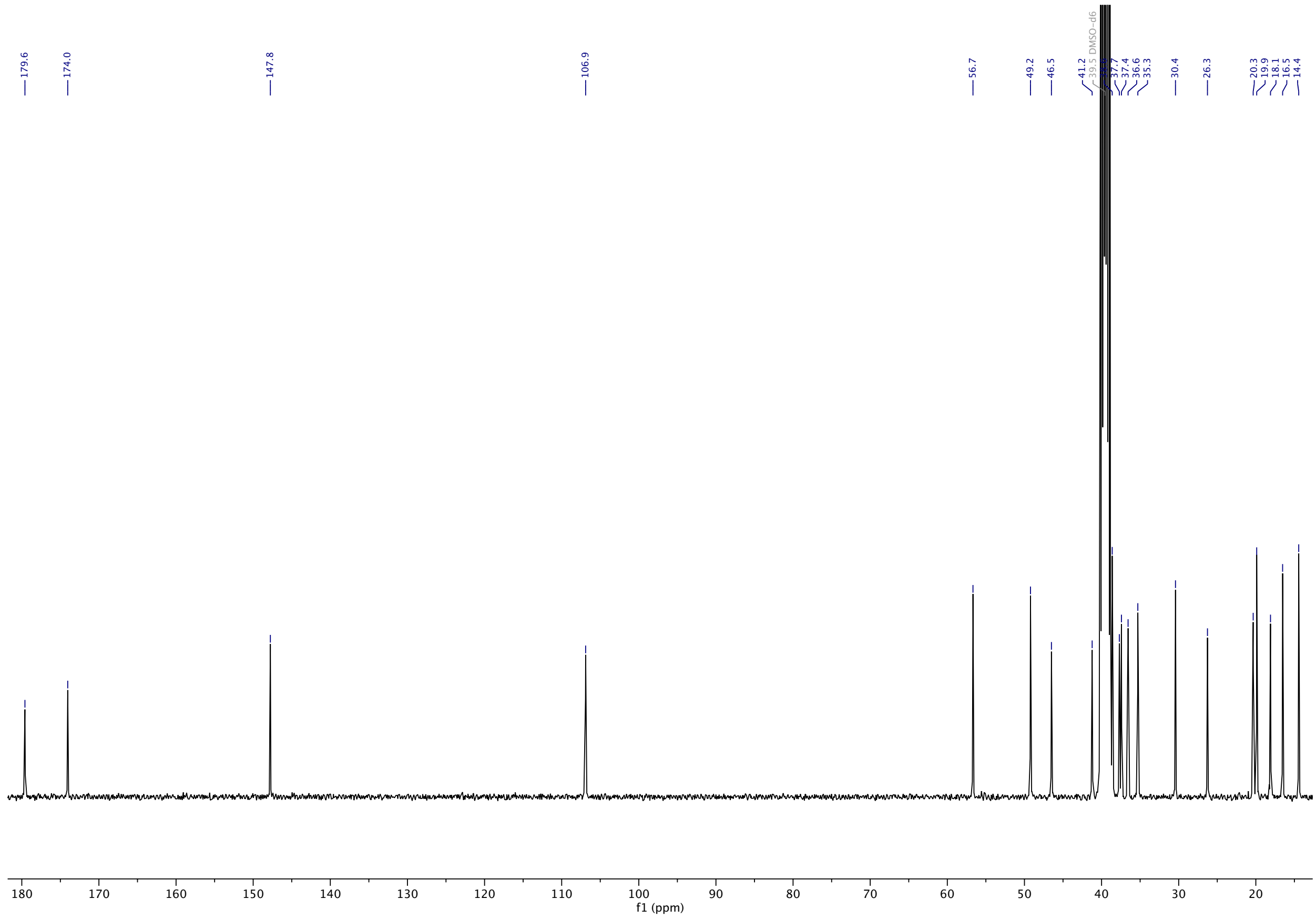
179.6
174.0
147.8
106.9
56.7
49.2
46.5
41.2
39.5 DMSO-d6
37.7
37.4
36.6
35.3
30.4
26.3
20.3
19.9
18.1
16.5
14.4
f1 (ppm)

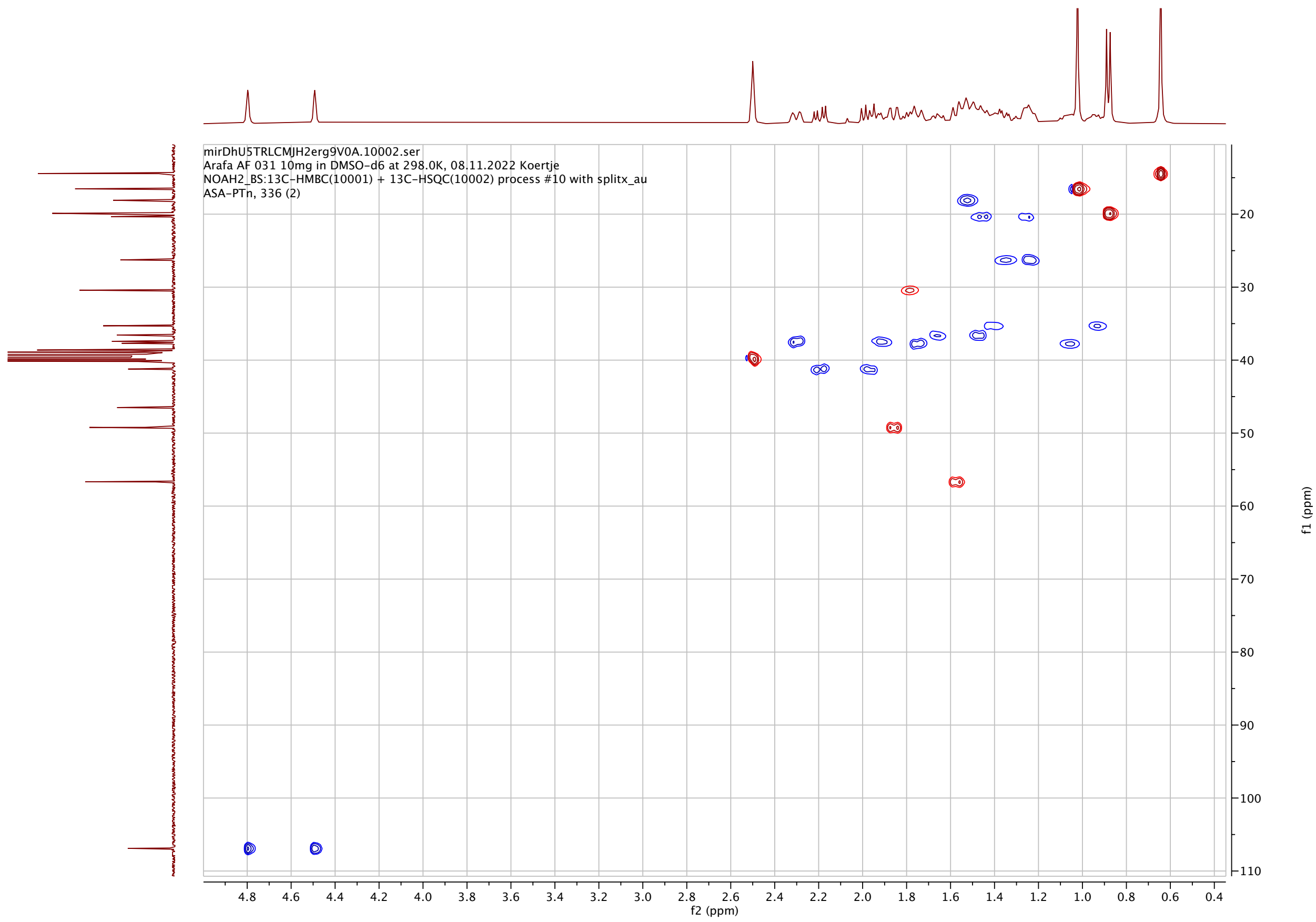
mirDhU5TRLCMJH2erg9V0A.10002.ser
Arafa AF 031 10mg in DMSO-d6 at 298.0K, 08.11.2022 Koertje
NOAH2_BS:13C-HMBC(10001) + 13C-HSQC(10002) process #10 with splitx_au
ASA-PTn, 336 (2)
f2 (ppm)
f1 (ppm)

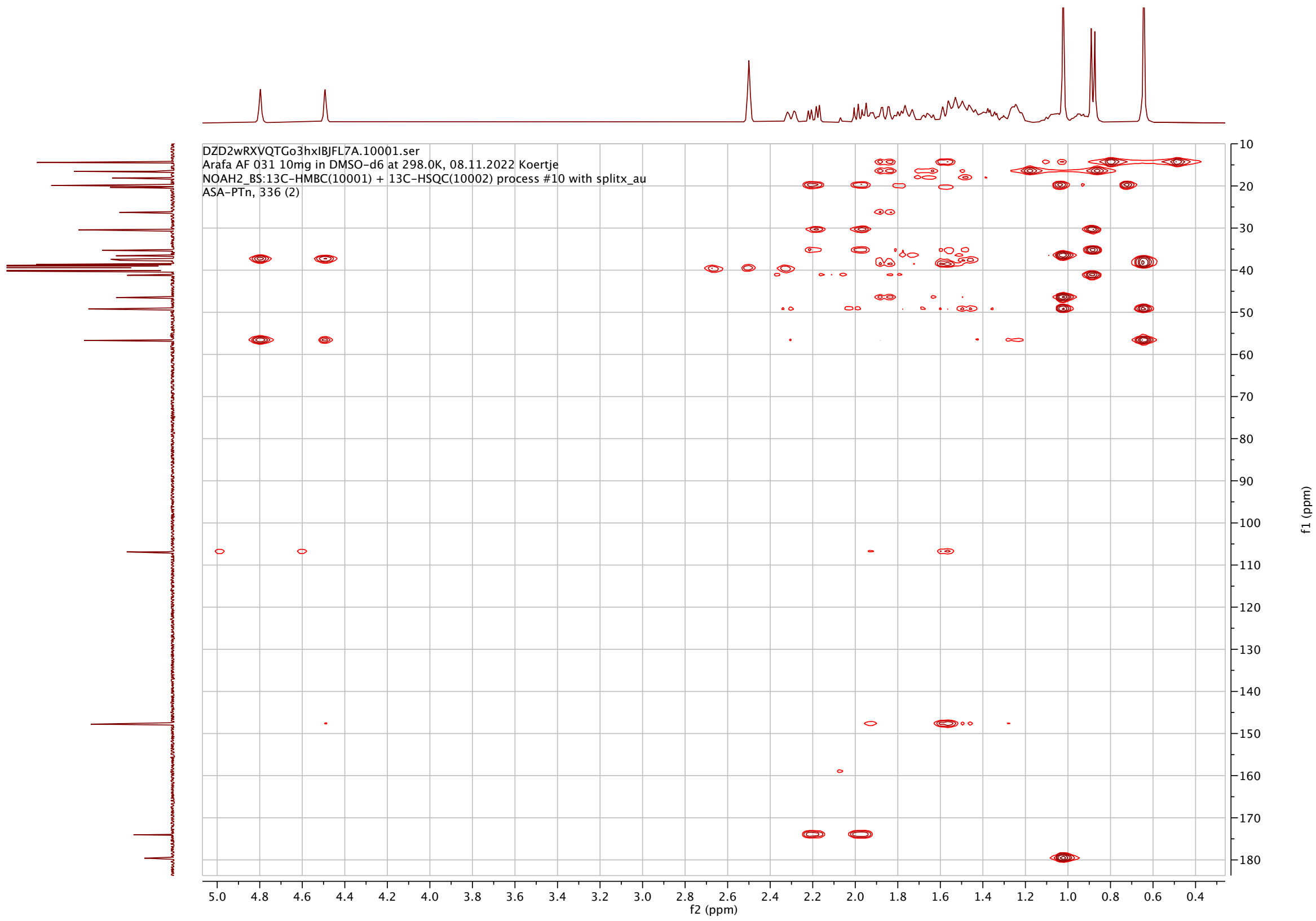
DZD2wRXVQTGo3hxIBJFL7A.10001.ser
Arafa AF 031 10mg in DMSO-d6 at 298.0K, 08.11.2022 Koertje
NOAH2_BS:13C-HMBC(10001) + 13C-HSQC(10002) process #10 with splitx_au
ASA-PTn, 336 (2)
f2 (ppm)
f1 (ppm)

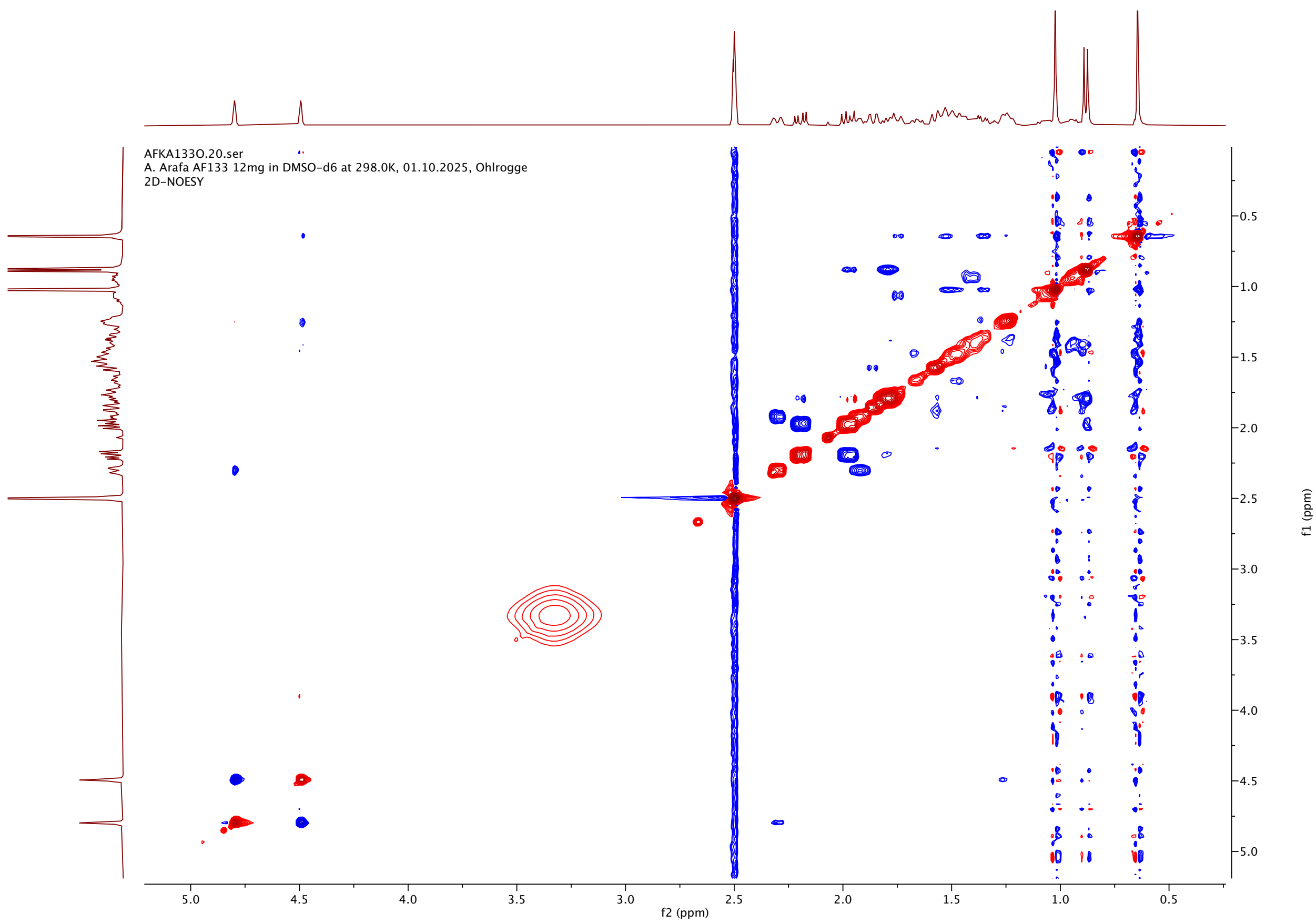
AFKA133O.20.ser
A. Arafa AF133 12mg in DMSO-d6 at 298.0K, 01.10.2025, Ohlrogge
2D-NOESY
f2 (ppm)
f1 (ppm)

4.4.10 3α-Hydroxy-*ent*-labd-8(17)-en-15-oic acid **22**

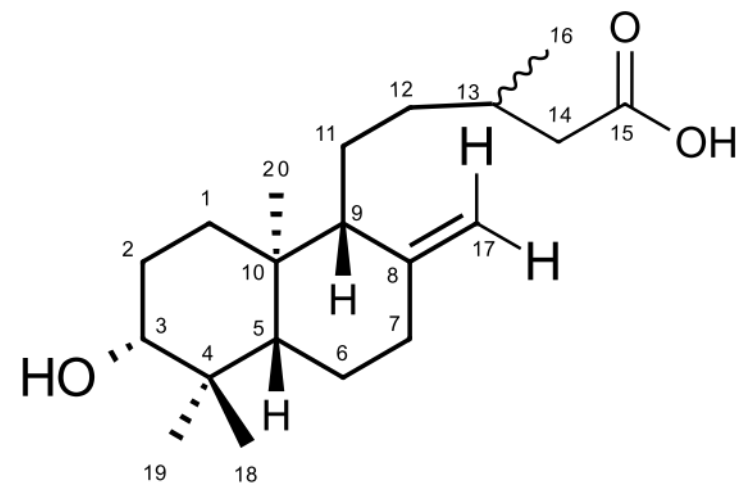

Pos.	Measured in $CDCl_3$, 298K			
	δ_C, 151 MHz	δ_H (J/Hz), 600 MHz	COSY	HMBC (H to C)
**1**	37.1	a 1.17, m, overlapped	-	-
		b 1.78, ddd, (3.7, 7.3, 13.1)	1a, 2a, 2b	3, 5
**2**	27.9	a 1.58, m	1b, 2b	3
		b 1.70, m, overlapped	-	-
**3**	78.7	3.24, dd, (4.3, 11.8)	2a, 2b	4, 18, 19
**4**	39.1	-	-	-
**5**	54.6	1.07, dd, (2.8, 12.5)	6a, 6b	4, 6, 7, 9, 10, 19, 20
**6**	23.9	a 1.37, m, overlapped	-	-
		b 1.72, m, overlapped	-	-
**7**	38.2	a 1.96, m	7b, 6a, 6b, 17b	6, 8, 17
		b 2.39, ddd, (2.4, 4.2, 12.8)	7a, 6a, 6b	5, 8, 9, 17
**8**	148.0	-	-	-
**9**	56.6	1.53, brd, 10.3	11a, 17a, 17b	8, 10, 11, 12, 17, 20
**10**	39.4	-	-	-
**11**	21.0	a 1.41, m, overlapped	-	-
		b 1.44, m, overlapped	-	-
**12**	35.6	a 1.14, m, overlapped	-	-
		b 1.35, m, overlapped	-	-
**13**	30.8	1.91, m.	16	12, 14, 15, 16
**14**	41.2	a 2.16, dd, (8.1, 15.0)	14b, 13	12, 13, 15, 16
		b 2.31, dd, (6.0, 15.0)	14a, 13	12, 13, 15, 16
**15**	176.2	-	-	-
**16**	19.6	0.97, d, (6.6)	13	12, 13, 14
**17**	106.5	a 4.47, brs	17b, 9	7, 8, 9
		b 4.81, brs	17a, 9, 7a	7, 9
**18**	28.3	0.99, s	-	3, 4, 5, 19
**19**	15.4	0.76, s	-	3, 4, 5, 18
**20**	14.4	0.67, s	-	1, 5, 9, 10

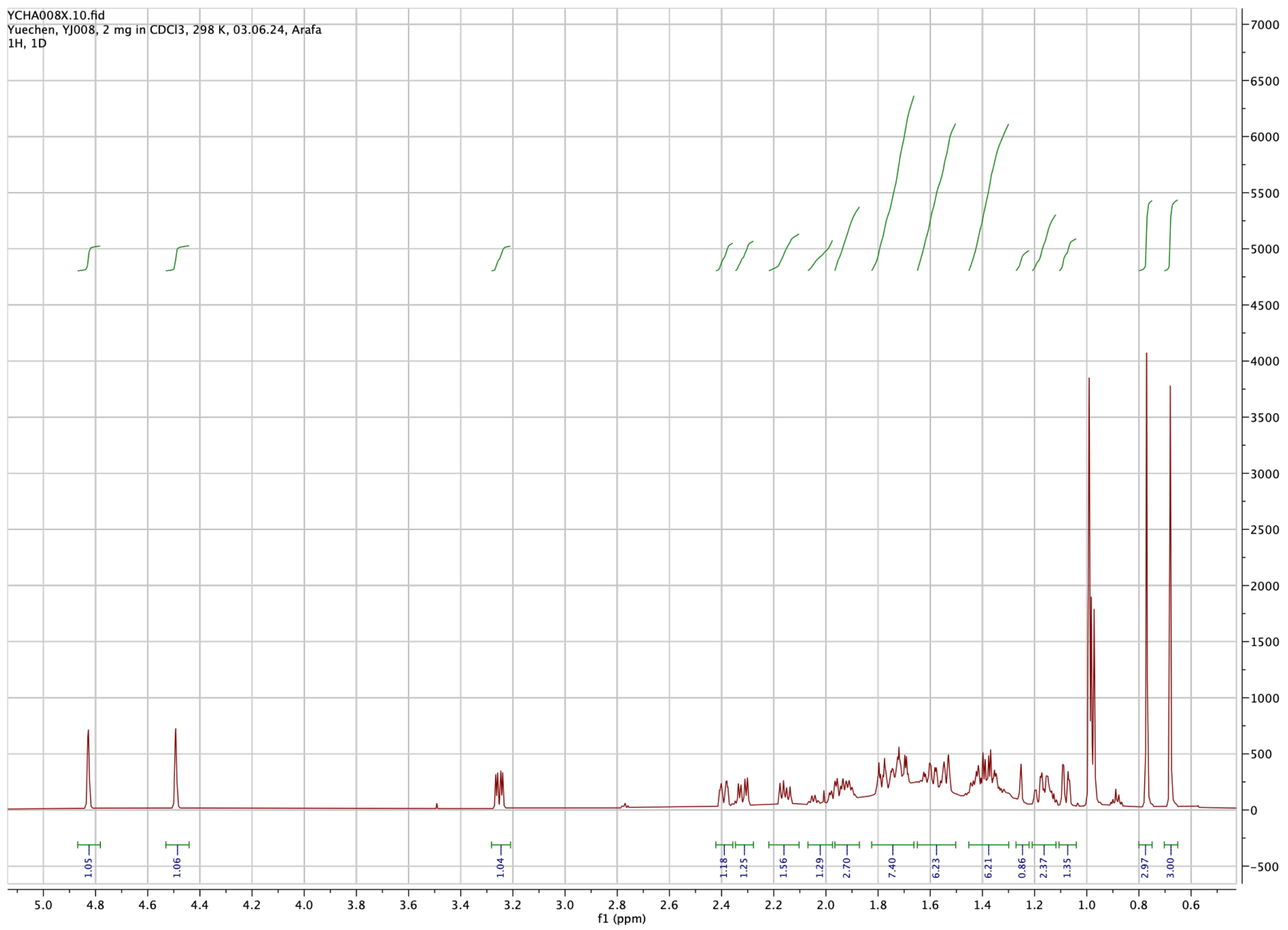
YCHA008X.10.fid
Yuechen, YJ008, 2 mg in CDCl3, 298 K, 03.06.24, Arafa
1H, 1D
f1 (ppm)

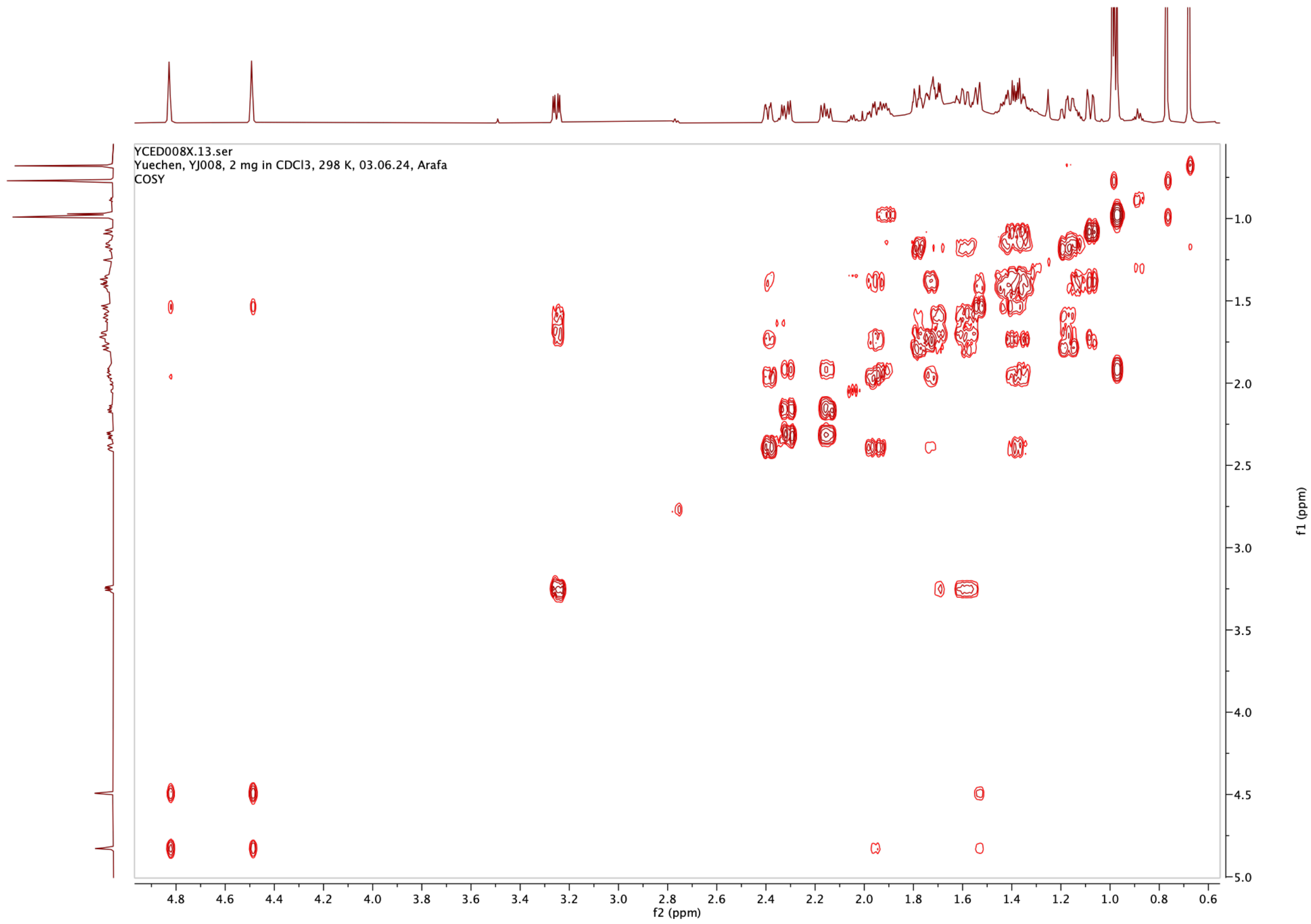
YCED008X.13.ser
Yuechen, YJ008, 2 mg in CDCl3, 298 K, 03.06.24, Arafa
COSY
f2 (ppm)
f1 (ppm)

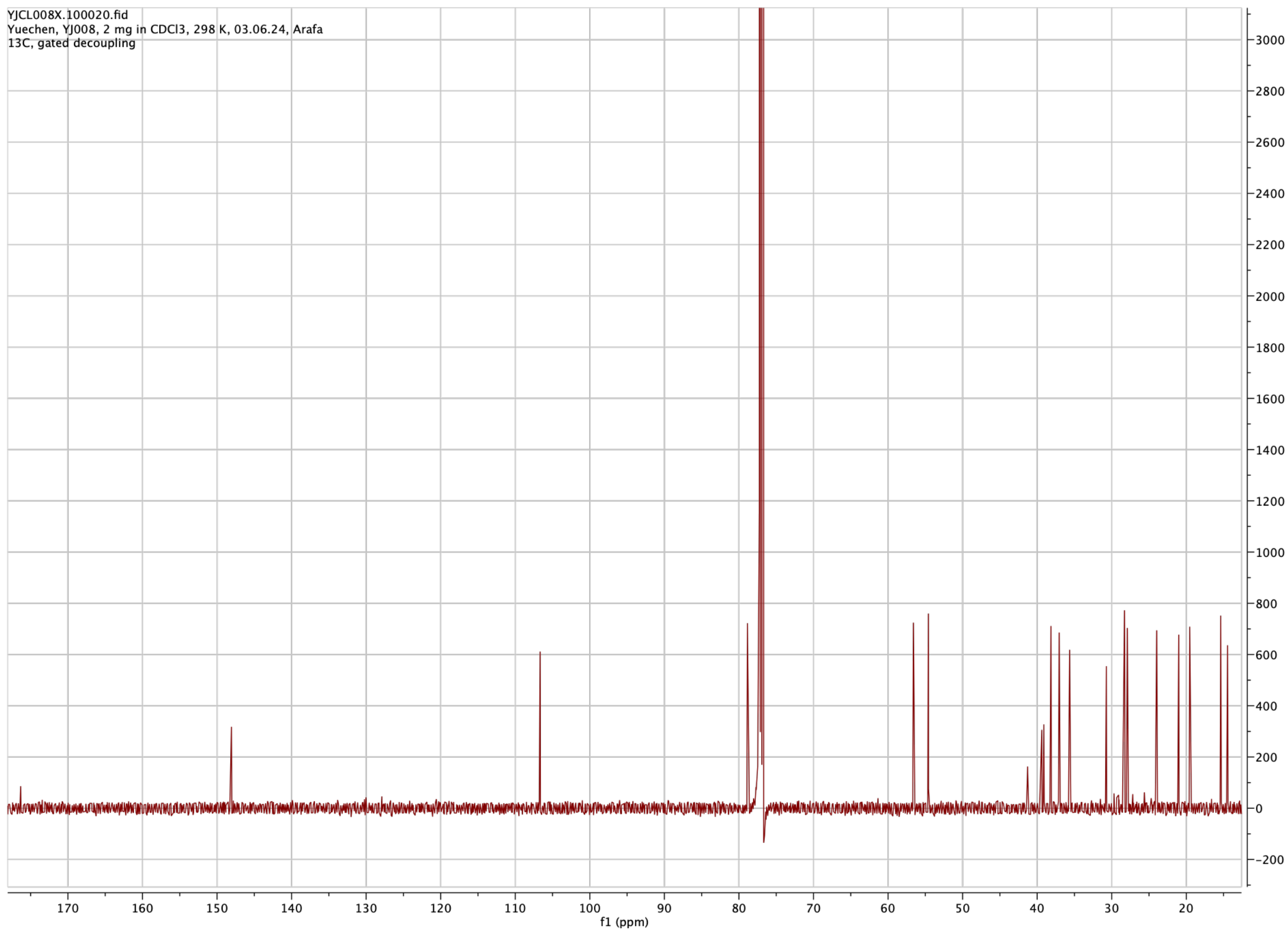
YJCL008X.100020.fid
Yuechen, YJ008, 2 mg in CDCl3, 298 K, 03.06.24, Arafa
13C, gated decoupling
f1 (ppm)

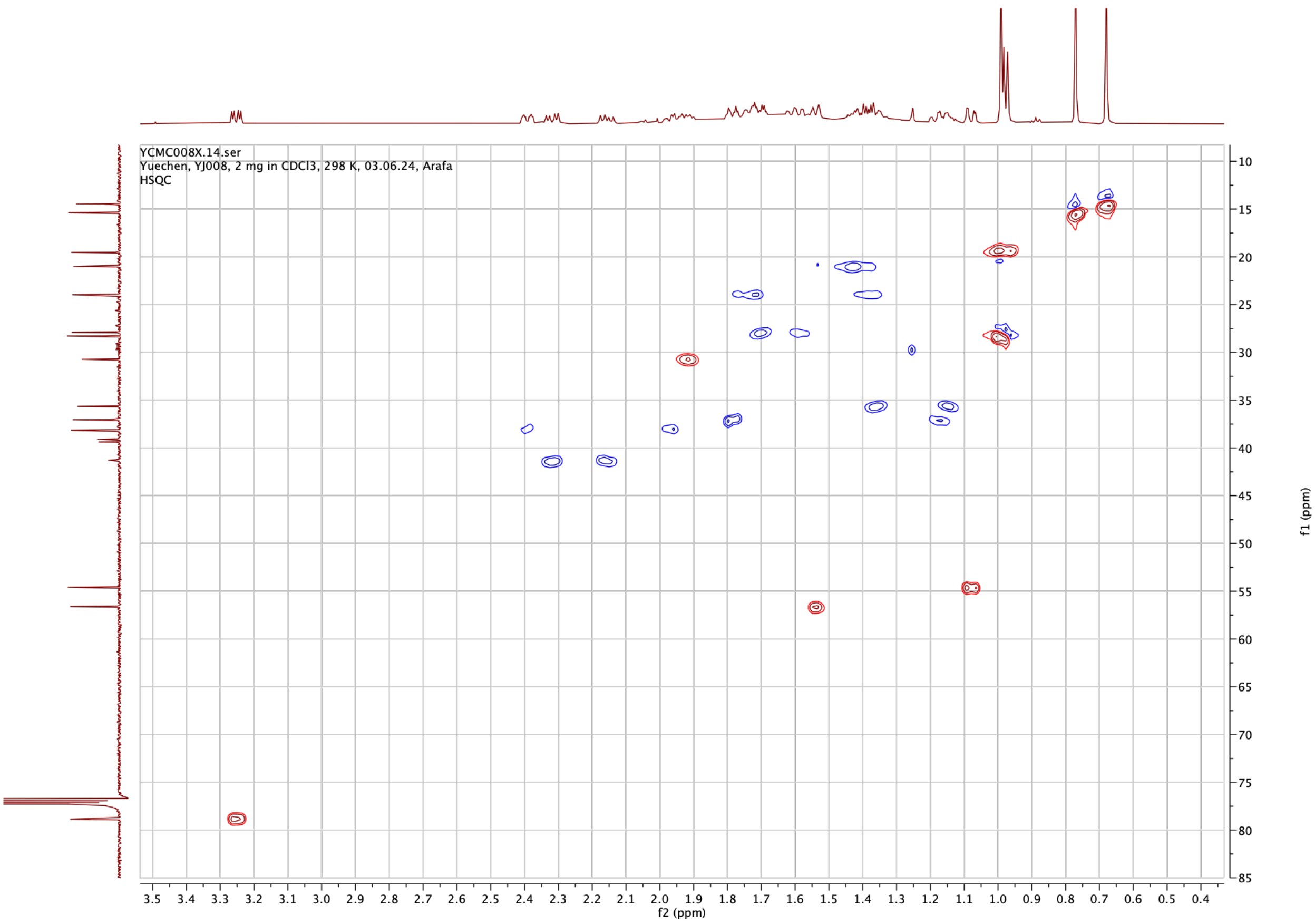
YCMC008X.14.ser
Yuechen, YJ008, 2 mg in CDCl3, 298 K, 03.06.24, Arafa
HSQC
f2 (ppm)
f1 (ppm)

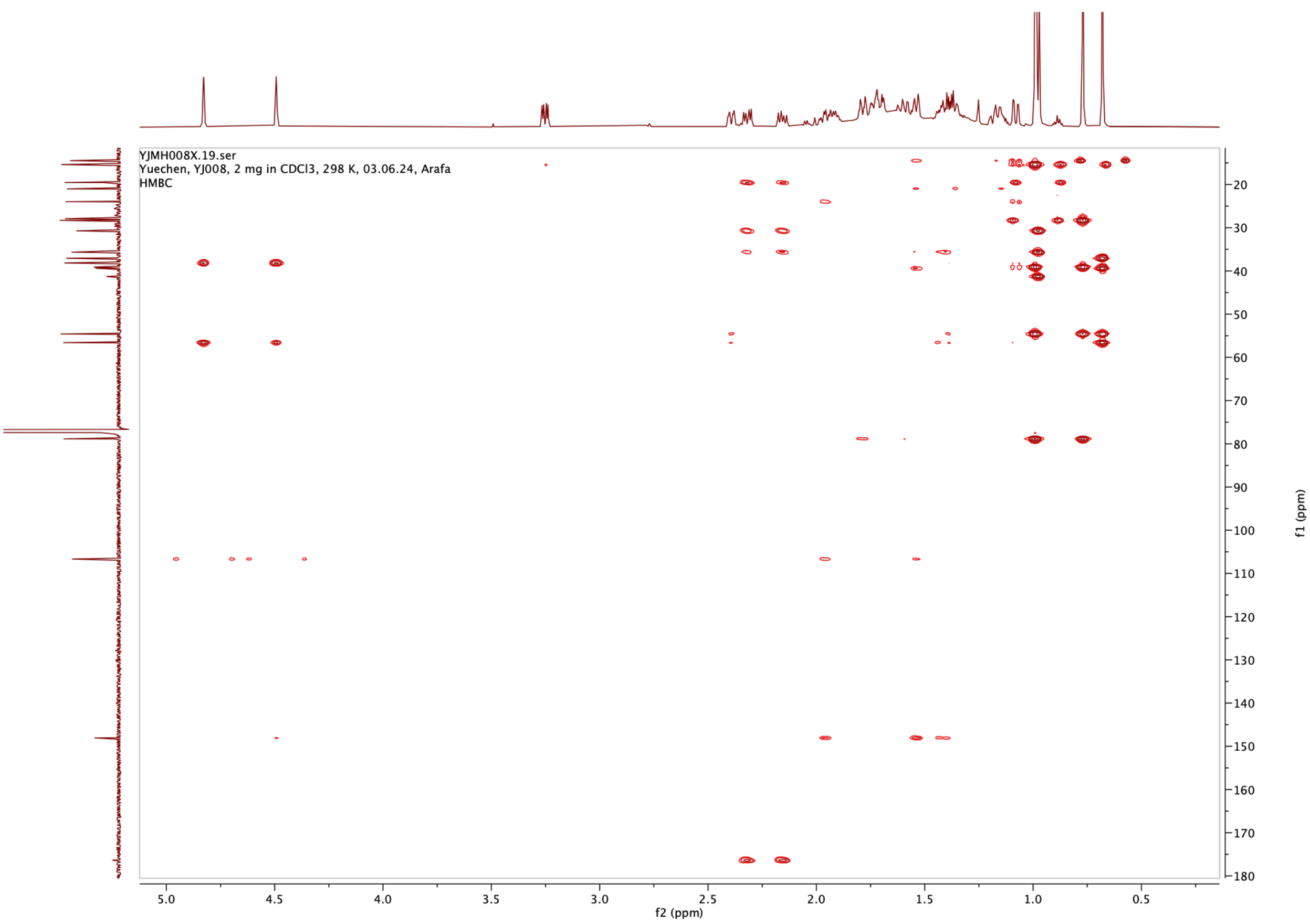
YJMH008X.19.ser
Yuechen, YJ008, 2 mg in CDCl3, 298 K, 03.06.24, Arafa
HMBC
f2 (ppm)
f1 (ppm)

4.5 HRMS data of Isolated Compounds

Name	Structure	Calc. HRMS	Meas. HRMS
6-*E*-3,12-Dihydroxy-3,7,11-trimethyltetradec-6-enedioic acid **13**	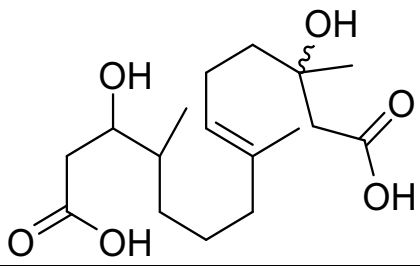	Calculated for $C_{17}H_{29}O_6^-$ 329.1970	Found 329.1956
4-*E*,8-*E*-12-Hydroxy-4,8,12-trimethyltetradeca-4,8-dienedioic acid **14**	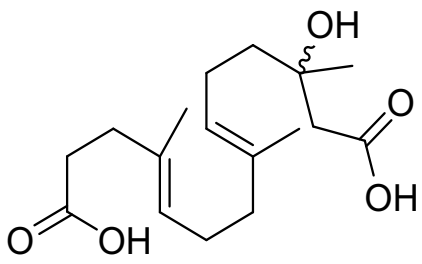	Calculated for $C_{17}H_{27}O_5^-$ 311.1864	Found 311.1859
6-*E*-10-Hydroxy-2,6,10-trimethyldodec-6-enedioic acid **15**	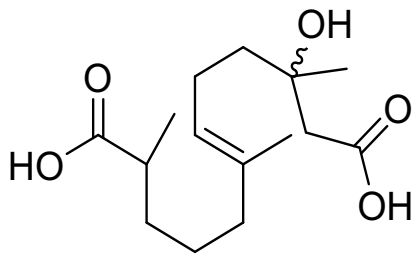	Calculated for $C_{15}H_{25}O_5^-$ 285.1707	Found 285.1696

Name	Structure	Calc. HRMS	Meas. HRMS
3α,13,18-Trihydroxy-*ent*-labd-8(17)-en-15-oic acid **16**	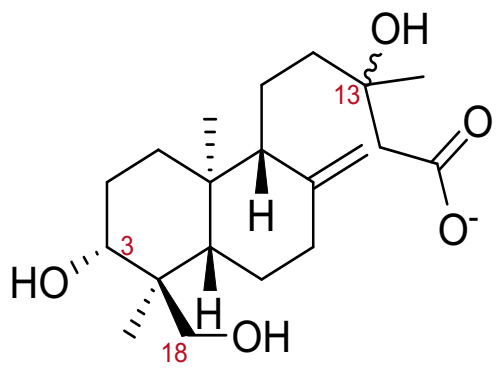	Calculated for $C_{20}H_{33}O_5^-$ 353.2333	Found 353.2333
ent-13 Hydroxy-labd-8(17)-en-15,18-dioic acid **17**	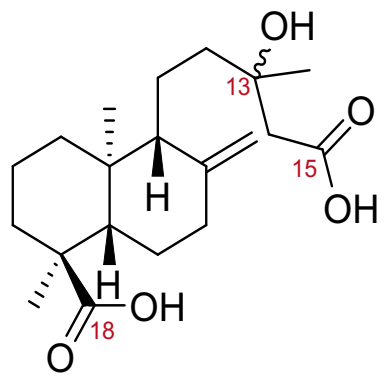	Calculated for $C_{20}H_{31}O_5^-$: 351.2177	Found 351.2144
3α,13-Dihydroxy-*ent*-labd-8(17)-en-15-oic acid **18**	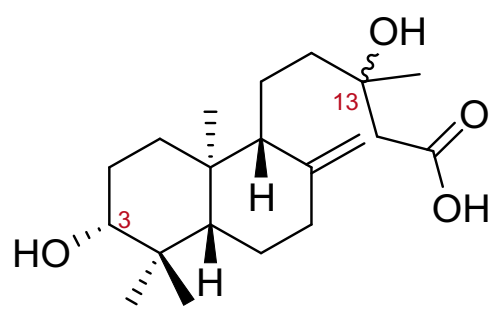	Calculated for $C_{20}H_{33}O_4^-$: 337.2384	Found 337.2367
13,18-Dihydroxy-*ent*-labd-8(17)-en-15-oic acid **19**	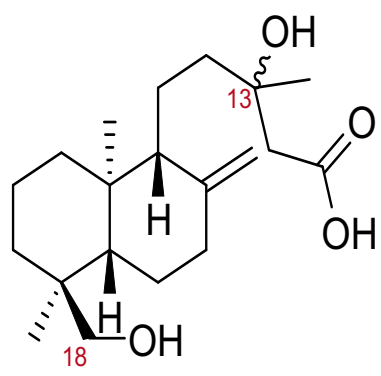	Calculated for $C_{20}H_{33}O_4^-$ 337.2384	Found 337.2359
ent-Labd-8(17), 13-dien-15,18-dioic acid **20**	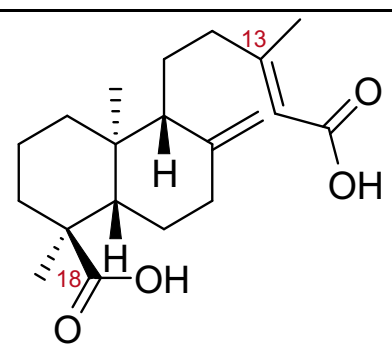	Calculated for $C_{20}H_{29}O_4^-$ 333.2071	Found 333.2048
ent-labd-8(17)-en-15,18-dioic acid **21**	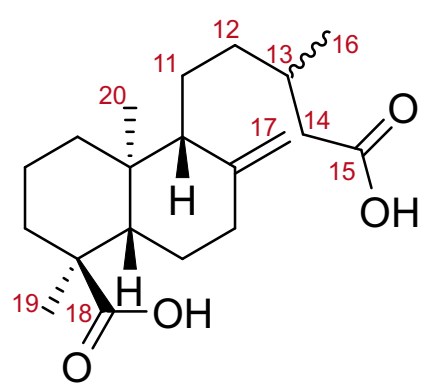	Calculated for $C_{20}H_{31}O_4^-$ 335.2228	Found 335.2228

3α-Hydroxy-*ent*-labd-8(17)-en-15-oic acid **22**	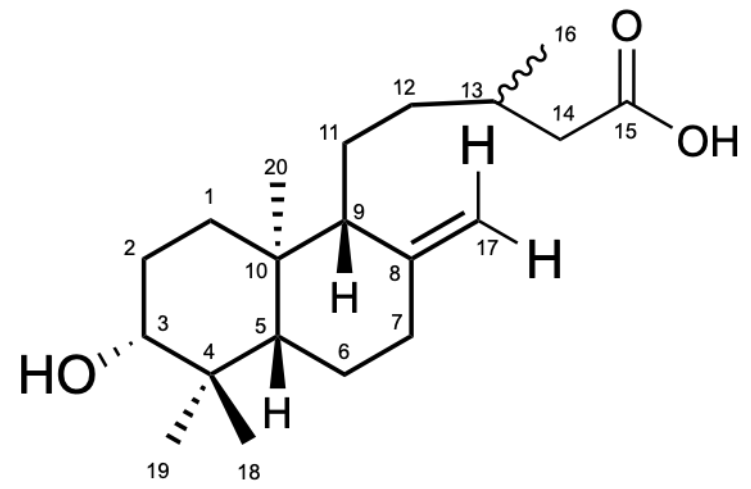	Calculated for $C_{20}H_{33}O_3^-$ 321.2435	Found 321.2443
